# Exploring the insulin secretory properties of the PGD_2_-GPR44/DP2 axis *in vitro* and in a randomized phase-1 trial of type 2 diabetes patients

**DOI:** 10.1371/journal.pone.0208998

**Published:** 2018-12-17

**Authors:** Stanko Skrtic, Björn Tyrberg, Malin Broberg, Hans Ericsson, Volker Schnecke, Magnus Kjaer, Marcus Hompesch, Eva-Marie Andersson, Erik Ryberg, Alexander Aivazidis, Charlotte Wennberg Huldt, Lars Löfgren, Linda Morrow, Joanna Parkinson, Tina Rydén-Bergsten, Elaine Watkins, Maria Sörhede Winzell

**Affiliations:** 1 Cardiovascular, Renal and Metabolism, IMED Biotech Unit, AstraZeneca Gothenburg, Mölndal, Sweden; 2 Department of Endocrinology, Sahlgrenska University Hospital and Institute of Medicine, Sahlgrenska Academy, University of Gothenburg, Gothenburg, Sweden; 3 ProSciento Inc, Chula Vista, CA, United States of America; University of Illinois College of Medicine, UNITED STATES

## Abstract

**Aims/Hypothesis:**

GPR44 (*DP2*, *PTGDR2*, *CRTh2*) is the receptor for the pro-inflammatory mediator prostaglandin D_2_ (PGD_2_) and it is enriched in human islets. In rodent islets, PGD_2_ is produced in response to glucose, suggesting that the PGD_2_-GPR44/DP2 axis may play a role in human islet function during hyperglycemia. Consequently, the aim of this work was to elucidate the insulinotropic role of GPR44 antagonism *in vitro* in human beta-cells and in type 2 diabetes (T2DM) patients.

**Methods:**

We determined the drive on PGD_2_ secretion by glucose and IL-1beta, as well as, the impact on insulin secretion by pharmacological GPR44/DP2 antagonism (AZD1981) in human islets and beta-cells *in vitro*. To test if metabolic control would be improved by antagonizing a hyperglycemia-driven increased PGD_2_ tone, we performed a proof-of-mechanism study in 20 T2DM patients (average 54 years, HbA1c 9.4%, BMI 31.6 kg/m^2^). The randomized, double-blind, placebo-controlled cross-over study consisted of two three-day treatment periods (AZD1981 or placebo) separated by a three-day wash-out period. Mixed meal tolerance test (MMTT) and intravenous graded glucose infusion (GGI) was performed at start and end of each treatment period. Assessment of AZD1981 pharmacokinetics, glucose, insulin, C-peptide, glucagon, GLP-1, and PGD_2_ pathway biomarkers were performed.

**Results:**

*W*e found (1) that PGD_2_ is produced in human islet in response to high glucose or IL-1beta, but likely by stellate cells rather than endocrine cells; (2) that PGD_2_ suppresses both glucose and GLP-1 induced insulin secretion *in vitro;* and (3) that the GPR44/DP2 antagonist (AZD1981) in human beta-cells normalizes insulin secretion. However, AZD1981 had no impact on neither glucose nor incretin dependent insulin secretion in humans (GGI AUC _C-peptide 1-2h_ and MMTT AUC _Glucose 0-4h_ LS mean ratios vs placebo of 0.94 (80% CI of 0.90–0.98, p = 0.12) and 0.99 (90% CI of 0.94–1.05, p = 0.45), despite reaching the expected antagonist exposure.

**Conclusion/Interpretation:**

Pharmacological inhibition of the PGD_2_-GPR44/DP2 axis has no major impact on the modulation of acute insulin secretion in T2DM patients.

**Trial registration:**

ClinicalTrials.gov NCT02367066.

## Introduction

Type 2 diabetes mellitus (T2DM) is characterized by a relative loss of functional beta-cell mass leading to insufficient insulin secretion during insulin resistance. It is believed that beta-cell insufficiency is caused by a combination of beta-cell death (apoptosis) and loss-of-function. Beta-cell functional loss may to some degree be explained by dedifferentiation to a cell without the capacity to produce or secrete insulin [1]. Therefore, disease modification in T2DM requires functional beta-cell mass restoration [2] through mechanisms preventing apoptosis or restoring loss-of-function. Although the beta-cell insulin secretory machinery is well understood and pharmacological treatments are available that increase insulin secretion, such as sulphonylureas and GLP-1 receptor agonists, there is a lack of drugs on the market that retain efficiency over time and truly modulate disease mechanisms coupled to insulin insufficiency. Prostaglandin D_2_ (PGD_2_) is a pro-inflammatory mediator where one of its receptors, GPR44 (DP2, *PTGDR2*, *CRTh2*) so far has been of particular interest to respiratory medicine [3]. Several selective GPR44/DP2 receptor antagonists were taken forward into clinical development as potential anti-asthmatic therapeutics, including AZD1981 developed by AstraZeneca [4–6]. Interestingly, in the human pancreas GPR44/DP2 is enriched in the islets versus the exocrine tissue [7, 8]. In fact, it is among the more highly expressed islet GPCRs according to a recent GPCRome survey [9]. Nevertheless, the function of this receptor in the endocrine pancreas is completely unknown. GPR44/DP2 is a G_αi/o_-coupled GPCR with a negative impact on cAMP production [10]. Rodent islets produce prostaglandins (including PGD_2_) locally [11] that have a negative impact on beta-cell function, including insulin secretion [11–13]. Consequently, locally produced PGD_2_ via GPR44/DP2 may act as a cAMP dependent break on insulin secretion mediated by glucose metabolic stimulation or other GPCRs, such as the GLP-1 receptor. Therefore, antagonizing the GPR44/DP2 pathway in T2DM to restore insulin secretion via a potentially disease driving mechanism may be an opportunity to develop a disease modifying drug in diabetes.

Here we present the first translational study to investigate the potential gluco-metabolic role of the PGD_2_-GPR44/DP2 pathway in T2DM, spanning from in vitro studies in human islets and beta-cells to a clinical proof-of-mechanism study in T2DM subjects.

## Material and methods

### Compound and in vitro pharmacology

The GPR44/DP2 antagonist, AZD1981, 4-(acetylamino)-3-[(4-chlorophenyl)thio]-2-methyl-1H-indole-1-acetic acid [6], previously a clinical candidate for treatment of asthma [5, 14] was investigated for treatment of T2DM. The biochemical and pharmacological properties of AZD1981 was recently described [14]. Here, the *in vitro* pharmacological properties of AZD1981 was further investigated in the human beta-cell line EndoC-betaH1, which has high expression of GPR44/DP2 (confirmed by qPCR, Fig Ba in S1 File). Briefly, EndoC-betaH1 cells [15] were plated in fibronectin- and extracellular matrix-coated 96-well plates for the *in vitro* pharmacology analyses and functional assays. Experimental details of the methods are described in the supporting information S1 File.

### Insulin and PGD_2_ secretion *in vitro* and gene expression analyses

The EC_50_ for PGD_2_ inhibition of glucose stimulated insulin secretion (GSIS) was determined using EndoC-betaH1 cells. The cells were incubated with varying concentrations of the stable PGD_2_ analogue 15(R)-15-methyl PGD_2_ (Cayman Chemical, USA) at 11.1 mM glucose and insulin secretion was determined. The EC_50_ value for PGD_2_ was calculated from the dose response curve. Since EndoC-betaH1 cells has no expression of L-PGDS (confirmed by qPCR, data not shown) and thus cannot produce PGD_2_, the stable PGD_2_ analogue was added at EC_80_ concentration and the effect of the specific GPR44/DP2 antagonist AZD1981 was determined at 11.1 mM glucose or in the presence of a GLP-1 receptor agonist (100 nM exendin-4; Bachem AG, Switzerland).

Human islets (Prodo Laboratories Inc. USA) were used to study PGD_2_ secretion, GSIS and for qPCR analysis of the gene expression of enzymes in the PGD_2_ synthesis pathway. The acute effect of PGD_2_ on GSIS was measured by incubating human islets for 1h in low (2.8mM) or high (11.1mM) glucose, with or without addition of 1 nM 15(R)-15-methyl PGD_2_. In other experiments, islets were incubated in high glucose (22.2 mM) with and without addition of 20 ng/ml interleukin 1-beta (IL-1beta) and PGD_2_ secretion into the culture media was determined after 24 h (PGD_2_ EIA, Cayman Chemical). The expression of genes in the PGD_2_ synthesis pathway and GPR44/DP2 were further studied by reanalyzing published human islet single cell sequencing data (MTAB-5061, EBI accession number) [16]. Experimental details are available in the supporting information S1 File.

### Clinical study design

This study (http://clinicaltrials.gov NCT02367066) was a phase 1, randomised, double-blind, placebo-controlled, multiple-dose, cross-over study conducted at a single centre. The aim was to explore the acute effects of GPR44/DP2 antagonism on both glucose and incretin dependent insulin secretion in T2DM. Hence a cross-over design was chosen with three-day treatment periods upon which steady state of the oral GPR44/DP2 antagonist AZD1981 was to be reached. The study consisted of a run-in, two three-day treatment periods separated by a wash-out period, and follow-up. During each treatment period, the pharmacokinetic (PK) and pharmacodynamic (PD) effects of AZD1981 or placebo were characterized in a mixed meal tolerance test (MMTT) [17] or by a graded glucose infusion (GGI) [18], respectively. A paracetamol test was conducted simultaneously with the MMTT to determine the effects of AZD1981 on gastric emptying.

The primary objectives were defined as change from baseline (day -1 to day 3) for endpoints; a) mixed meal tolerance test (MMTT) area under curve (AUC) (0-4h) for plasma glucose, b) MMTT Cmax and c) graded glucose infusion AUC (1-2h) for plasma C-peptide. The study was powered to detect therapeutically relevant changes for these primary variables. A number of secondary and exploratory variables all linked to insulin secretion or PK/PD were also assessed.

**Fig 1:**
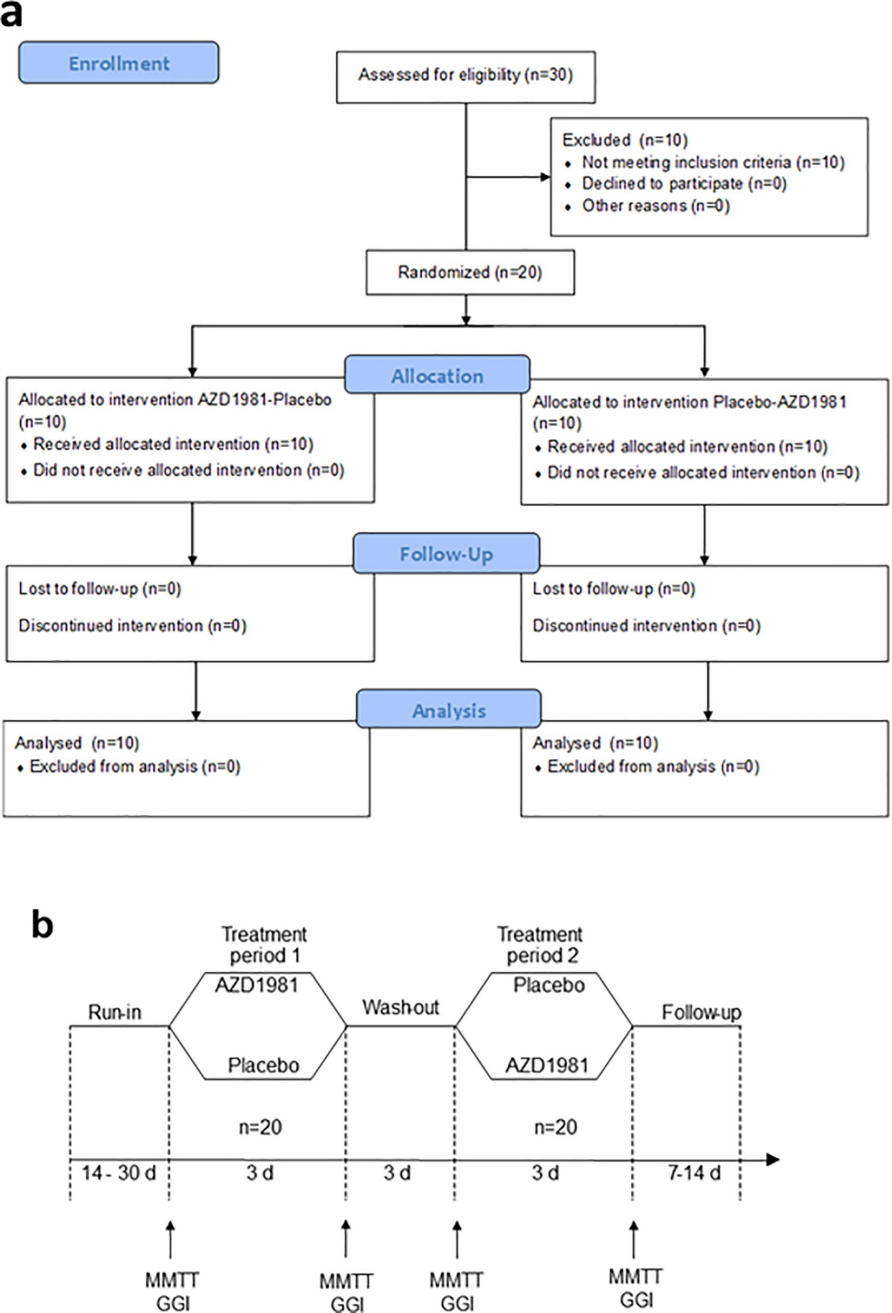
Study flow chart according to CONSORT 2010 guidelines. The design of a human Proof of Mechanism study to test the Mechanism of Action hypothesis. The phase 1 study was designed as a randomised, double-blind, placebo-controlled, multiple-dose, cross-over study, consisting of a run-in, two treatment periods separated by a wash-out period and a follow-up for each subject. The subjects were T2DM patients on metformin (n = 20).

The study was conducted at ProSciento Inc in Chula Vista, CA, USA and the study protocol was approved by the Institutional Review Board Schulman IRB in Research Triangle Park, NC, USA. All subjects gave their written voluntary informed consent before participation. Patients were enrolled between March 2015 and April 2015. See Fig 1 for study flow chart. More study details are available in the supporting information S1 File.

The enrolled patients were diagnosed with T2DM and had inadequate glycaemic control on a metformin regimen. Patients were adult males or females, had glycosylated haemoglobin (HbA1c) levels between ≥58.5mmol/mol (7.5%) and ≤97 mmol/mol (11%) at enrolment, were treated with metformin alone for at least the last three months, and had a fasting glucose level from 3–14 mmol/l (54–252 mg/dl). See Table 1 for subject demographics at screening.

**Table 1:** Demographic characteristics. Data are presented as mean ± SD.

	Screening
Gender (female/male)	7/13
Age (y)	54 ± 7.9
Body weight (kg)	87.4 ± 19.1
BMI (kg/m^2^)	31.6 ± 4.5
T2DM duration (y)	9.5 ± 4.6
Metformin daily dose (mg)	1830 ± 428
HbA1_c_ (mmol/mol, DCCT %)	79 ± 9.2 (9.4 ± 1.1)
fGlucose (mg/dl)	167.4± 39.4
fInsulin (IU/ml)	15.9 ±7.7
FFA (mEq/l)	0.49 ± 0.2
Total Cholesterol (mg/dl)	182.3 ± 35.2
Triglycerides (mg/dl)	125.7 ± 69
hsCRP (mg/l)	3.2 ± 2.6
eGFR (ml/min/1.73)	104.5 ± 11.7
SBP (mm/Hg)	128.4 ± 10.3
DBP (mm/Hg)	80.9 ± 6
Pulse (beats/min)	69 ± 11
BMR (kcal/d)	1679 ± 332

Abbreviations: BMI = Body Mass Index, BMR = Basal Metabolic Rate (Harris Benedict equation), DBP = Diastolic Blood Pressure, eGFR = estimated Glomerular Filtration Rate, f = fasting, FFA = Free Fatty Acids, HbA_1c_ = Haemoglobin A_1c_, hsCRP = high sensitive C-Reactive Protein, SBP = Systolic Blood Pressure, T2DM = Type 2 Diabetes Mellitus.

### Pharmacokinetic and pharmacodynamics sampling in clinical study

Patients were fasting overnight and all oral medications including metformin were dispensed 75 min prior to the first PD assessment (MMTT). During the MMTT (Ensure Plus drink, 473 ml, 100g carbohydrates), blood sampling was conducted at baseline, 15, 30, 60, 90 and 120 min post the mixed meal intake. During the GGI, by 20% glucose intravenous infusion over 2h with a constant rate GLP-1 infusion the last hour, blood sampling was conducted at 10 min intervals. Blood samples were obtained for assessments of AZD1981 PK on days 2, 3, 8, and 9 concurrent with the MMTT and GGI, and for paracetamol PK on days 3 and 9 during the MMTT. More details are available in the supporting information S1 File.

### Analytical techniques in clinical study

Bedside plasma glucose during GGI was measured by an YSI-2300 analyzer (YSI Life Science, USA). For MMTT & GGI plasma insulin, C-peptide, glucagon and total GLP-1 were analyzed by the respective ELISA (Mercodia AB, Sweden) and glucose by a colorimetric method. Plasma paracetamol and AZD1981 concentrations were measured by LC-MS (Covance Bioanalytical services, IN, USA). EDTA plasma and urine was collected on day -1, 3, 6 and 9 for analysis of exploratory PGD_2_ biomarkers performed at AstraZeneca. More details are available in the supporting information S1 File.

### Sample size calculations & statistical analysis

The clinical study was powered to detect therapeutically relevant changes for the primary variables. The sample size for the MMTT was based on internal studies that estimated the intra-individual standard deviation (SD) for ln(MMTT AUC_Glc 0-4h_) to be 0.1. With this estimated SD and an alpha of 0.1, one-sided test and at least 8% difference versus control in MMTT AUC_Glc 0-4h_, a sample size of 20 evaluable patients was determined to provide at least 80% power. The 8% difference was chosen based on the rational that it is a clinically relevant magnitude as this is the effect observed by DPPIV inhibitors [19]. Similarly, the planned sample size for the GGI was determined based on an estimated SD of ln(GGI AUC_C-pep 1-2h_) as 0.17. Based on similar power calculations, a sample size of 20 evaluable patients was determined to provide at least 80% power to detect at least a 20% difference versus control for GGI AUC_C-pep 1-2h_.

Summary statistics are provided for all of the PK and PD variables assessed. The PK evaluable population was used to assess the PK endpoints. PK parameters were derived using actual sampling times and standard non-compartmental methods, and summarized descriptively for both AZD1981 and paracetamol. The three primary PD variables were the MMTT AUC_Glc 0-4h_, MMTT_Glc-Cmax_, and the GGI AUC_C-pep 1-2h_ (PD evaluable population). The difference in primary variables between AZD1981 and placebo were analyzed using a mixed-effects model that includes treatment (2 levels), treatment sequence (2 levels), and period (2 levels) as fixed effects, baseline values of the dependent variable as a covariate, and subject-within-sequence as a random effect, were used to analyze the difference in MMTT AUC_Glc 0-4h_, MMTT_Glc-Cmax_, and the GGI AUC_C-pep 1-2h_ between AZD1981 and placebo. We assumed that there was no carryover effect in the crossover study as the 3 day wash-out period should have appropriately removed any impact from carryover between treatment periods. The primary variables were log-transformed prior to analysis and then transformed back to linear scale. Significance was established 1-sided at 10%. Treatment estimates were provided as estimated least square mean ratios with a 80% 2-sided CI. All secondary endpoints of the PD parameters and the pre-hepatic ISR (fasting β-cell responsiveness) was analyzed similarly as those described for the primary PD endpoints. No tests for multiplicity testing were performed for the secondary endpoints. The SAS MIXED procedure was used for the statistical analysis.

Pre-hepatic insulin secretion rate (ISR) over time was calculated using deconvolution of peripheral C-peptide concentrations during MMTT using the method of Hovorka et al [20].

## Results

### Expression of GPR44/DP2 and PGD_2_ synthesis pathway genes

**Fig 2:**
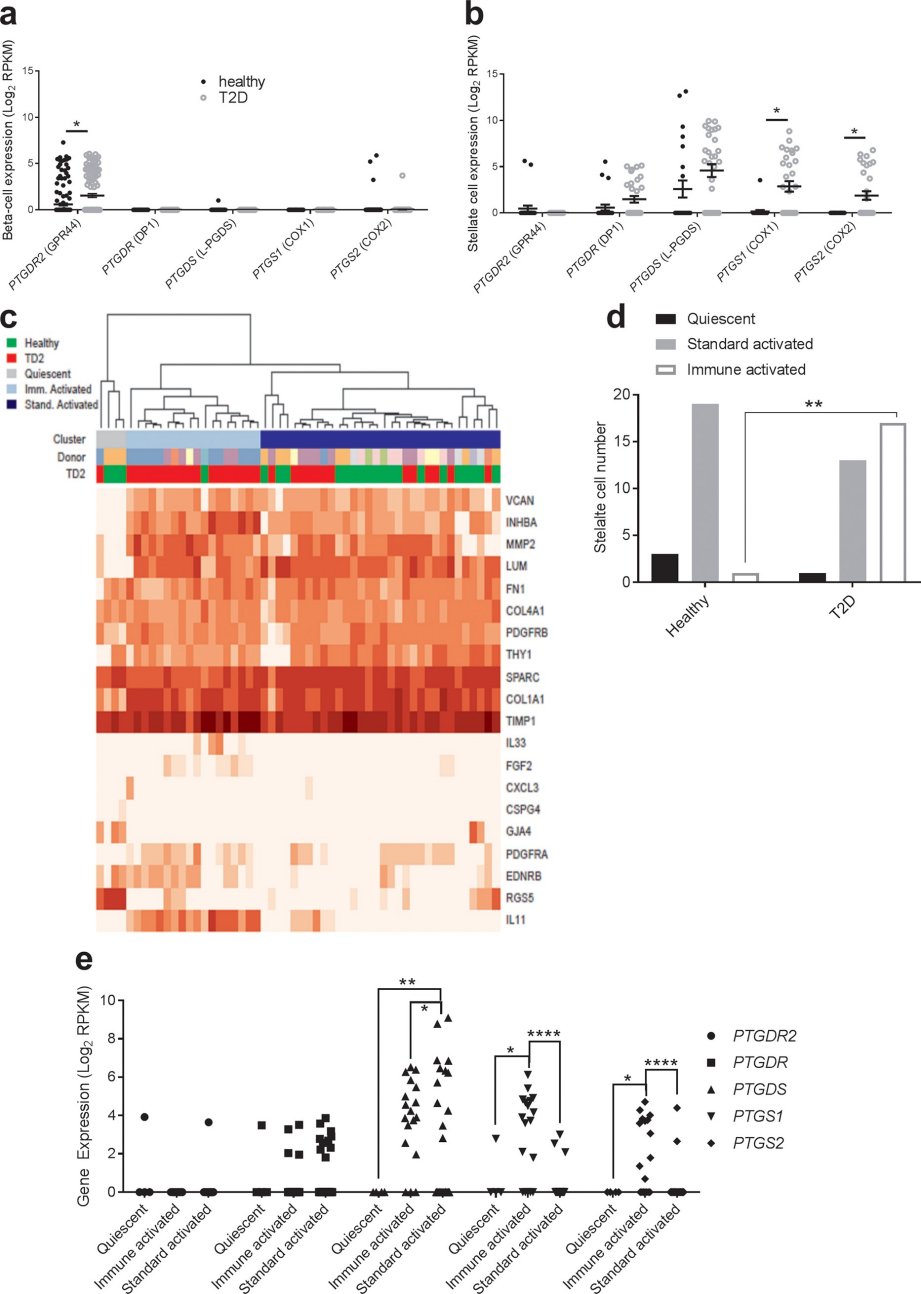
Tissue expression of GPR44/DP2 and PGD_2_ related genes and characterization of pancreatic stellate cells. *PTGDR2* (GPR44/DP2) mRNA expression in human beta-cells (Healthy, n = 197; T2DM, n = 112) (a) and pancreatic stellate cells (Healthy, n = 23; T2DM, n = 31) (b). Heat map of stellate cell signature gene expression sub-classified as quiescent, immune activated (imm) and standard activated (stand) stellate cells according to the description in the main text (c). Quantification of stellate cell subpopulations in healthy and T2D donor human islets (d). Expression of GPR44/DP2 and PGD_2_ related genes in subclasses of stellate cells (e). Data are presented as mean ± SEM (a-b), total number of cells (d), and individual cells (e). *p<0.05, **p<0.01, ***p<0.001, ****p<0.0001.

Since the expression of GPR44/DP2 (*PTGDR2*) in the pancreas is restricted to the islets with similar protein expression in both healthy and T2DM [8] we decided to further characterize the expression of GPR44/DP2 and genes related to PGD_2_ synthesis in all islet cell types by mining a single cell sequencing data set [16]. We found that GPR44/DP2 is prominently expressed in beta-cells (Fig 2A), whereas the related receptor DP1 (*PTGDR1*) and the genes in the PGD_2_ synthesis pathway mainly are found in pancreatic stellate cells from both healthy and T2DM donors (Fig 2B). GPR44/DP2 mRNA was marginally upregulated in beta-cells from T2DM donors, but several genes in the PGD_2_ synthesis pathway were quite substantially upregulated in the stellate cells (Fig 2A and 2B). Interestingly, the total number of immune-activated stellate cells (according to classification by Baron *et al* [21]) was higher in T2DM donors and the expression of the PGD_2_-related genes were particularly prominent in this subpopulation of stellate cells (Fig 2C–2E). Other endocrine cells types do not express these genes, except GPR44/DP2 that is expressed also in alpha-, delta-, and PP-cells (Fig A in S1 File). Together, the gene expression pattern suggests that PGD_2_ is produced in the islet by activated stellate cells, particularly in T2DM, and may impact beta-cell function through activation of GPR44/DP2.

### PGD_2_ secretion by human islets

**Fig 3:**
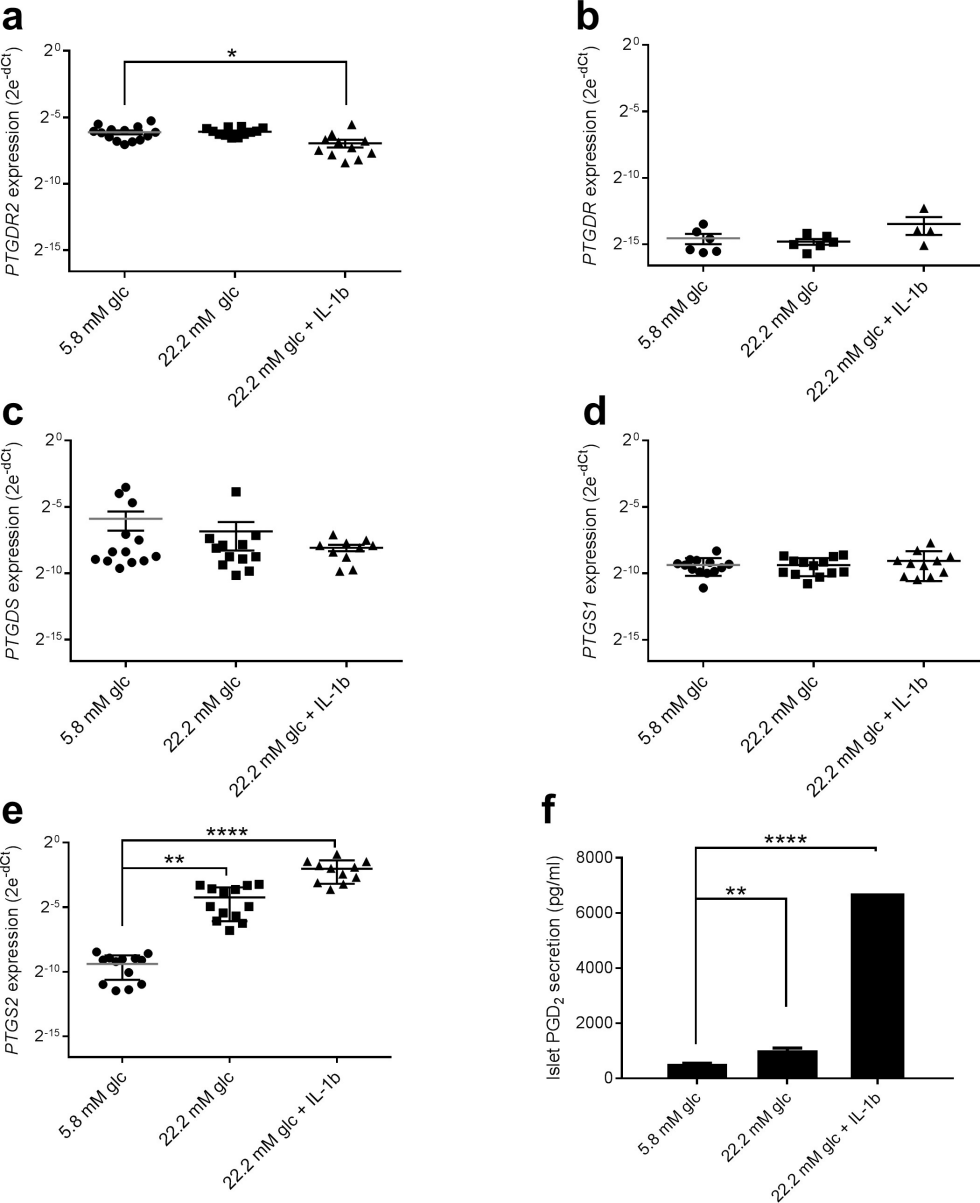
Human islet gene expression and regulation by high glucose with and without IL-1beta. Expression of *PTGDR2* (GPR44/DP2) (a), *PTGDR1* (DP1) (b), *PTGDS* (L-PGDS) (c), *PTGS1* (COX-1) (d) and *PTGS2* (COX-2) (e). *In vitro* human islet PGD_2_ secretion (f). Data are presented as mean ± SEM from 5 different human islet donors, where each condition was evaluated in quadruplicates. Glc = glucose. *p<0.05, **p<0.01, ****p<0.0001.

To confirm activation of the GPR44/DP2 pathway during metabolic stress, human islets were treated with high glucose with or without IL-1beta. GPR44/DP2 was highly expressed in intact human islets (Fig 3A, Fig Ba in S1 File), while DP1 was expressed at very low levels (Fig 3B, Fig Bb in S1 File), as expected based on the findings that only a small pool of cells in islets express DP1 (stellate cells, see above). Interestingly, a significant reduction in GPR44/DP2 expression was observed after incubation with high glucose and IL-1beta, while high glucose in itself had no impact (Fig 3A). PGD_2_ is produced from arachidonic acid via enzymatic activity of phospholipase A, the cyclooxygenases (*PTGS*) 1 and 2, and lipocalin-prostaglandin D_2_ synthase (*PTGDS*). *PTGDS* and *PTGS1* gene expression were not significantly affected by either high glucose or IL-1beta (Fig 3C and 3D), while *PTGS2* was significantly increased by high glucose (35-fold compared to 5.6 mM glucose, p<0.01) and even more prominent by IL-1beta (165-fold compared to control, p<0.0001; Fig 3E).

After incubation of human islet for 24 h, degraded PGD_2_ accumulated in the cell culture media at low glucose (5.6 mM) (Fig 3F). A significant increase in PGD_2_ production was observed at 22.2 mM glucose with a 2-fold increase (p<0.01), which was further potentiated by IL1-beta with a 10-fold increase in accumulated PGD_2_ over 24h (p<0.0001; Fig 3F).

### Effect of PGD_2_ and the GPR44/DP2 antagonist AZD1981 in human beta cells

**Fig 4:**
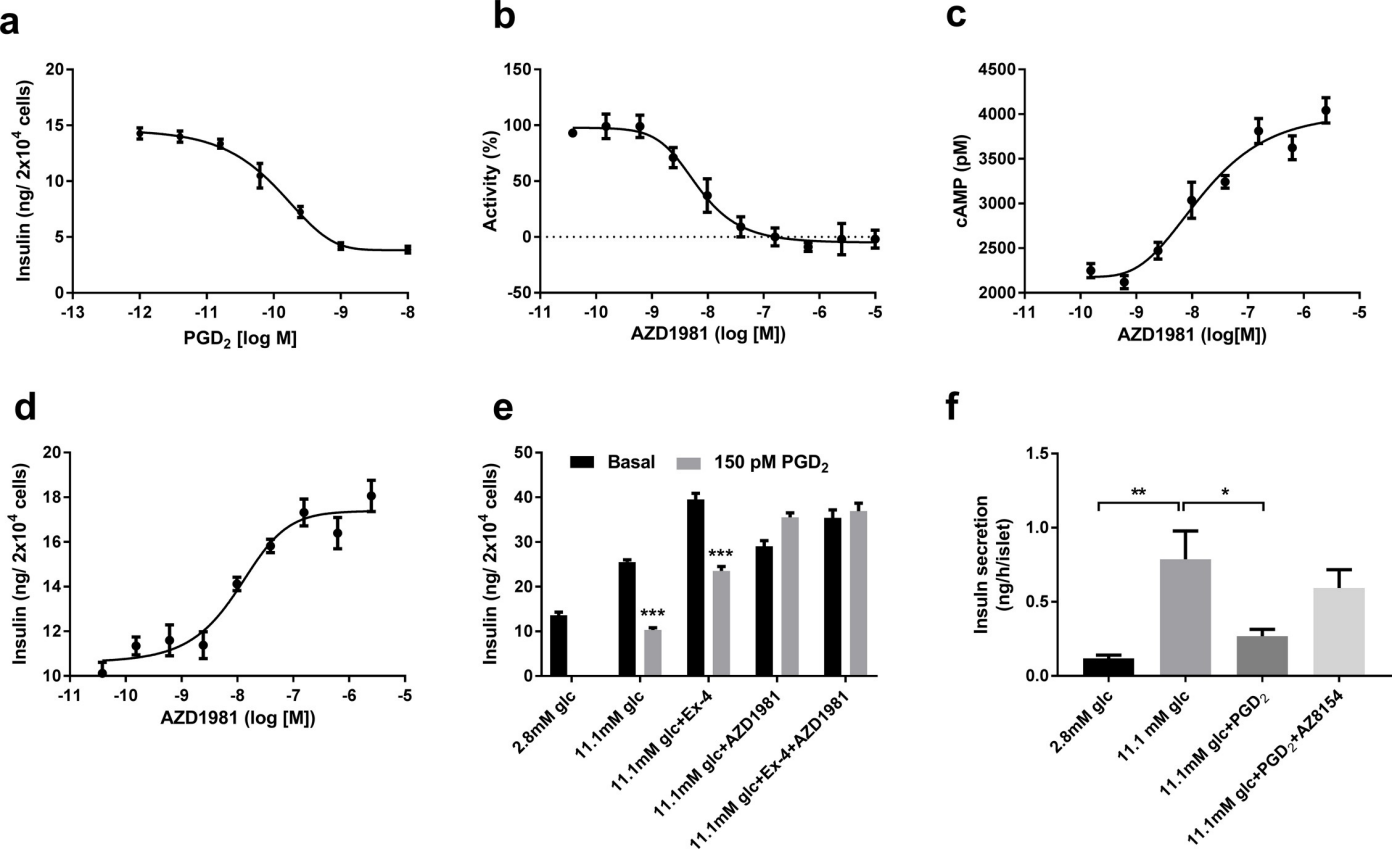
Effect of PGD_2_ on insulin secretion and *in vitro* pharmacology of AZD1981 in the human beta-cell line EndoC- betaH1. Dose response curve for the PGD_2_ analogue (15(R)-15-methyl-PGD_2_) on insulin secretion, from which the EC_50_ was determined (a). The *in vitro* potency of the GPR44/DP2 antagonist AZD1981 was determined using DMR (IC_50_ = 4.9±1nM) (b), cAMP (IC_50_ = 21±8nM) (c) and by glucose-stimulated insulin secretion (IC_50_ = 13±2nM) (d). The assays were performed in the human beta cell line EndoC-betaH1 with addition of 150 pM 15(R)-15-methyl-PGD_2_. Insulin secretion in EndoC-betaH1 cells was determined with and without addition of 150pM 15(R)-15-methyl-PGD_2_ at 11.1 mM glucose and 100nM exendin-4 (GLP-1 receptor agonist). 100nM of AZD1981 was added to restore GSIS. The effect of PGD_2_ on human islet insulin secretion was studied in islets from three donors where each incubation condition was run with 5 islets in 6 replicates (f). Data are presented as mean ± SEM.*p<0.05, **p<0.01***p<0.001.

Since the endogenous ligand for GPR44/DP2, PGD_2_, is unstable, a stable analogue, 15(R)-15-methyl-PGD_2_, was used to determine the effects on insulin secretion in human beta-cells (EndoC-betaH1). Activation of GPR44/DP2 by 15(R)-15-methyl-PGD_2_ resulted in a dose dependent inhibition of GSIS (Fig 4A). EC_50_ was calculated using three methods; DMR- dynamic mass distribution [22], cAMP and GSIS (Fig C in S1 File). The potency of AZD1981 on GPR44/DP2 signaling in EndoC cells were established at a constant concentration (150 pM, EC_80_) of the stable PGD_2_ analogue using the same three assays (DMR, cAMP and GSIS). All three assays correlated well and resulted in EC_50_ values for AZD1981 in the same range (Fig 4B–4D). Additional pharmacological profiling of AZD1981 and GPR44/DP2 in EndoC cells are presented in Figures D and E in S1 File.

Addition of 150 pM 15(R)-15-methyl-PGD_2_ to EndoC cells potently inhibited both GSIS as well as exendin-4-potentiated insulin secretion (Fig 3E). PGD_2_ inhibition of insulin secretion induced by both glucose and exendin-4, was completely restored by addition of 100 nM AZD1981 (Fig 4E). The inhibitory effect of 15(R)-15-methyl-PGD_2_ on GSIS was confirmed in human islets (Fig 4F). The GPR44/DP2 antagonist used in this experiment was however different from AZD1981but with similar potency in all three of the in vitro potency assays described for AZD1981 (data not shown).

**Fig 5:**
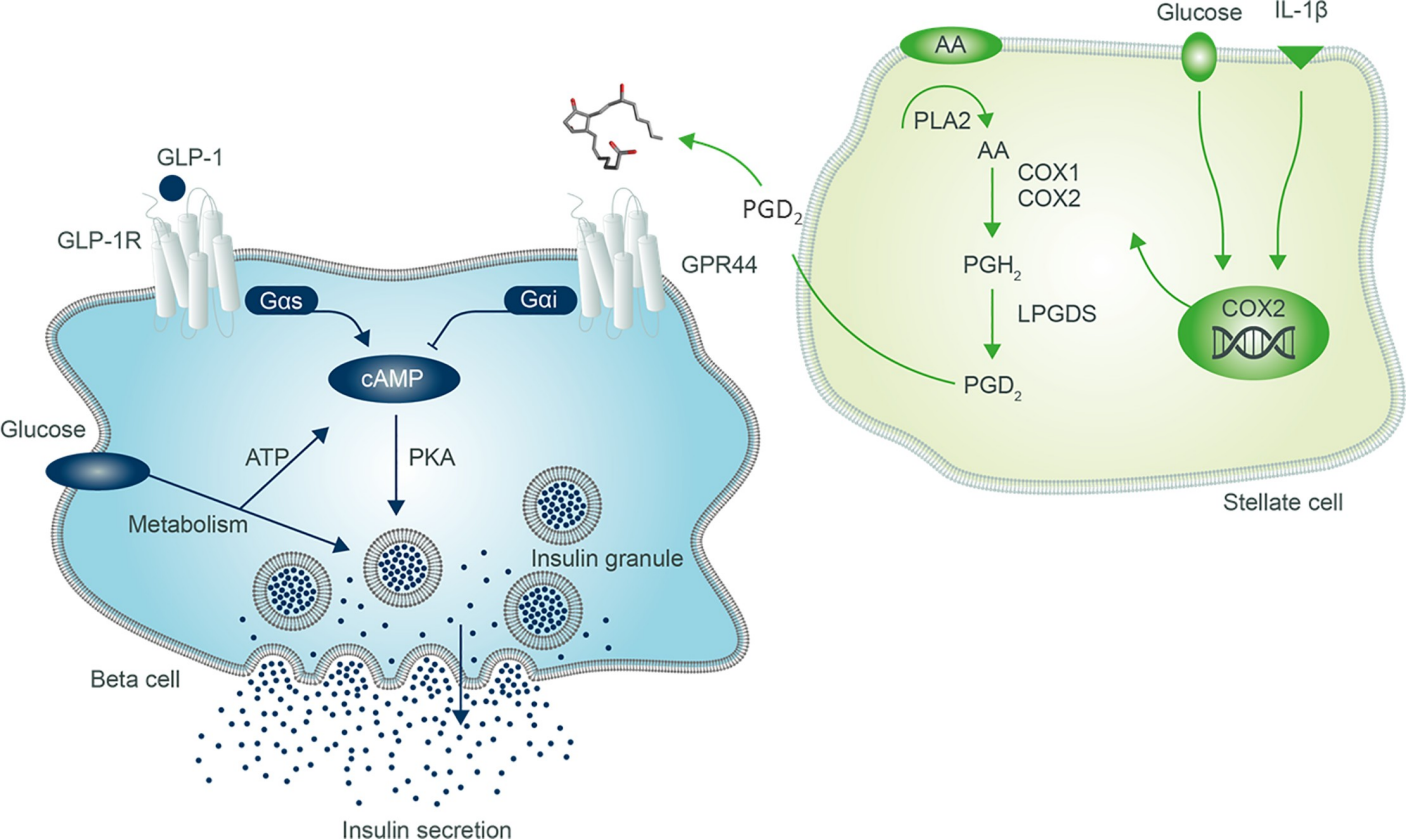
Hypothesis for the endogenous PGD_2_-GPR44/DP2 axis mechanism of action in islets. Glucose and IL-1beta induced *PTGS2* (COX-2) expression in stellate cells which stimulates the production of PGD_2_ synthesis. PGD_2_ is secreted and may act in a paracrine fashion on GPR44/DP2 on beta-cells, inhibiting cAMP and thereby reducing GSIS.

We demonstrate that GPR44/DP2 is activated in human beta cells by stress induced either by high glucose or high glucose in combination with IL-1beta, resulting in dysregulated glucose and GLP-1 stimulated insulin secretion in vitro, at least partly through production of PGD_2_ in stellate cells. Based on that we formulated a hypothesis that stellate cells are activated by glucose and cytokines, producing PGD_2_, which in turn acts on GPR44/DP2 on the beta-cells, reducing intracellular cAMP levels leading to inhibition of GSIS (Fig 5). To test the hypothesis that the PGD_2_-GPR44/DP2 axis is activated in T2DM and can be a therapeutically relevant target in treatment of T2DM, a mechanistic clinical study was designed and executed using the oral GPR44/DP2 antagonist AZD1981. The study’s aim was to explore the acute effects of GPR44/DP2 antagonism on both glucose and incretin dependent insulin secretion in T2DM subjects. Hence, to investigate the insulinotropic action in a physiological context an oral challenge was given through a 4h MMTT [17] and also an intravenous glucose challenge, i.e. a graded glucose (GGI) ranging from a normoglycemic to hyperglycaemic levels [18].

The study outline is described in more detail both the Materials and Method section and illustrated in Fig 1 as well as in the supporting information S1 and S3 Files.

### Effects on glucose, insulin and C-peptide after MMTT and GGI in T2DM patients

**Fig 6:**
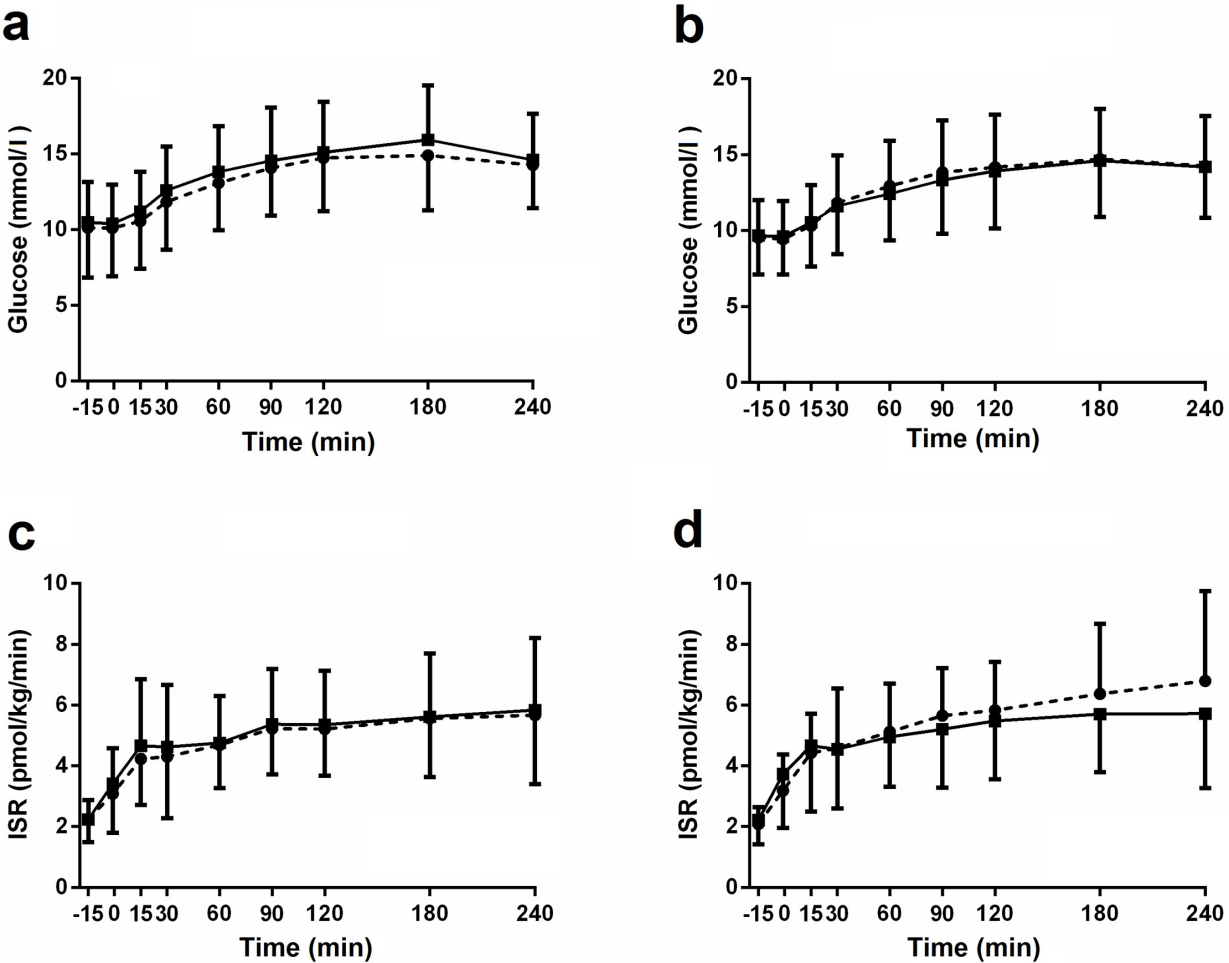
Mixed Meal Tolerance Test in T2DM patients. Glucose assessments at baseline (a), and after 3 days treatment (b) or Insulin Secretion Rate (ISR) at baseline (c) and after 3 days treatment (d). Circles connected with dotted lines represent placebo and squares connected with full lines represent AZD1981. Data are presented as mean ± SD.

Treatment with the GPR44/DP2 antagonist AZD1981 at steady state exposure (AZD1981 at 100 mg BID for three days) did not exert any clinically significant insulinotropic effect in T2DM patients on metformin (Fig 6 and Table 2) for any of the co-primary variables in the study. Neither following the insulinotropic challenge in a physiological context during an oral challenge eg a 4h MMTT (MMTT AUC_Glc 0-4h_, p = 0.12 and MMTT_Glc-Cmax_, p = 0.06) nor after the intravenous glucose challenge i.e. the graded glucose infusion from a normoglycemic to hyperglycaemic range (GGI AUC_C-pep 1-2h_ p = 0.41) In addition a number of linked secondary variables were also analysed in a similar manner with a mixed effect model to determine any treatment effect of AZD1981 but without any multiplicity test correction. In order to exclude confounding effects by hepatic insulin clearance, C-peptide secretion was evaluated with three parameters, C_max_, AUC_(0-4h),_ and deconvoluted prehepatic insulin secretion rate (ISR) according to Hovorka [20]. Notably, the hypothesized insulinotropic effect was not captured by ISR in any subject or time point after neither the MMTT nor the GGI tests. Further none of the secondary variables did show any significant differences from placebo apart from MMTT AUC _C-peptide 0-4h_ and GGI AUC _Glucose0-1h_ which showed mechanistically contradictory borderline significant effects of p = 0.04 and 0.045 respectively (Fig F in S1 File and Table 2).

**Table 2:** Results of mixed effects model for treatment differences in pharmacodynamic parameters before and after GPR44/DP2 antagonism treatment.

Pharmacodynamic variable	AZD1981 LS mean (SE) # (n = 20)	Placebo LS mean (SE)# (n = 20)	AZD1981/placebo Mean ratio$ (80% CI) (n = 20)	P-value$
MMTT AUC _Glucose 0-4h_ *	0.92 (0.02)	0.98 (0.02)	0.94 (0.90; 0.98)	0.12
MMTT Cmax Glucose *	0.93 (0.02)	0.97 (0.02)	0.96 (0.92; 0.99)	0.06
GGI AUC _C-peptide 1-2h_ *	1.05 (0.04)	1.06 (0.04)	0.99 (0.96; 1.03)	0.41
MMTT AUC _C-peptide 0-4h_	1.01 (0.03)	1.09 (0.03)	0.93 (0.88; 0.98)	0.04
MMTT Cmax _C-peptide 0-4h_	1.06 (0.04)	1.11 (0.04)	0.95 (0.89; 1.02)	0.17
MMTT AUC _Insulin 0-4h_	0.99 (0.04)	1.08 (0.04)	0.92 (0.85; 0.99)	0.06
MMTT AUC _Glucagon 0-4h_	1.02 (0.05)	0.97 (0.05)	1.05 (0.97; 1.14)	0.28
MMTT AUC _total GLP1 0-4h_	1.06 (0.06)	0.99 (0.05)	1.07 (0.97;1.19)	0.27
GGI AUC _C-peptide 0-1h_	1.02 (0.04)	1.03 (0.04)	0.99 (0.94; 1.05)	0.45
GGI AUC _Glucose0-1h_	1.01 (0.02)	0.97 (0.02)	1.04 (1.01; 1.06)	0.045
GGI AUC _Glucose 1-2h_	1.01 (0.01)	0.99 (0.01)	1.02 (1.00; 1.04)	0.06
GGI AUC _Insulin 0-1h_	1.04 (0.06)	1.07 (0.06)	0.97 (0.91; 1.04)	0.29
GGI AUC _Insulin 1-2h_	1.04 (0.06)	1.04 (0.06)	0.99 (0.91; 1.06)	0.37
GGI AUC _total GLP1 0-1h_	1.19 (0.06)	1.06 (0.05)	1.12 (1.00; 1.26)	0.09
AUC _Glucose 0-24h_	0.96 (0.02)	0.97 (0.02)	0.99 (0.97; 1.02)	0.34

* Primary study variables

# Ratio of Baseline to Endpoint. Data are presented as least squares geometric means with standard error.

$ The difference in primary variables between AZD1981 and placebo were analysed using mixed-effects models with treatment, treatment sequence, and period as fixed effects, baseline of the primary variable as a covariate, and subject-within-sequence as a random effect. Significance was established at 1-sided at 10%. Treatment estimates were provided as estimated mean ratios with 80% 2-sided CI.

Abbreviations: AUC = Area Under Curve, CI = confidence interval, CGGI = Graded Glucose Infusion, LS = Least square, MMTT = Mixed Meal Tolerance Test, PD = Pharmacodynamic.

### Effects on glucagon, GLP1, paracetamol, PK/PD, PGD_2_ biomarkers and safety in T2DM patients

To further delineate the mode of action and impact on the other major hormonal islet axes, insulin, glucagon and total GLP-1 were assessed during the MMTT and GGI challenges. However, as outlined in Table 2, AZD1981 had no significant effect on glucagon secretion or total GLP-1. To fully understand the impact on oral glucose disposal the change in oral paracetamol PK was used to assess the impact on gastric emptying concomitantly with the MMTT. In the paracetamol test, the 90% confidence intervals (CI) of the geometric least squares (LS) mean ratios (AZD1981 to placebo) for C_max_ and AUC_(0-t)_ were fully contained within the range of 0.80–1.25 (0.90–1.17 for C_max_, 0.91–1.20 for AUC_(0-t)_) and thus the results demonstrated that AZD1981 had no significant effect on gastric emptying in T2DM patients (Fig G in S1 File).

A PK/PD analysis was conducted between AZD1981 exposure (AUC) during either the MMTT or the GGI tests and all of the efficacy endpoints listed in Table 2. However, the PK/PD analysis indicated that no relationship existed between AZD1981 exposure and the MMTT or GGI efficacy variables (Fig H in S1 File).

We explored if we could identify responder sub-populations with high PGD_2_ tone by determining circulating surrogate biomarkers of local PGD_2_ and to correlate that with PD response. However, it was not possible to establish a correlation between efficacy and such biomarkers (Fig I in S1 File).

Finally, there were no AZD1981 safety and tolerability findings that could have impacted the PD effects (Tables A and B in S1 File). All of the adverse events (AEs) reported were mild, none resulted in withdrawal from the study, and there were no serious adverse events (SAEs). The incidence of AEs was similar in the AZD1981 (n = 5, 25%) and placebo (n = 6, 30%) treatments. Two (2) of the AEs reported following AZD1981 treatment (diarrhoea) were considered related to the investigational product. There were no unusual or clinically significant vital sign changes observed, and no significant differences between treatments. No clinically significant abnormal findings were detected by ECG or physical examination.

## Discussion

To our knowledge, this is the first study elucidating the effect of PGD_2_ on human islet function, although it has been known for almost 30 years that PGE_2_ inhibits glucose-stimulated insulin secretion [12]. Since then, PGE_2_ control of both beta-cell function and mass has been extensively studied and its receptor EP3 is a potential drug target for treatment of diabetes (recently reviewed by Carboneau et al. [23]). Taking that into account, the unknown impact of PGD_2_ on GSIS and the finding that GPR44/DP2 is highly expressed in human beta-cells [7, 8], motivated us to explore the role of PGD_2_ in human beta-cell function.

We hypothesized that GPR44/DP2 modulates beta-cell function through an impact on the insulin secretory machinery, based on tissue expression of the GPR44/DP2 pathway genes and robust functional *in vitro* human islet/beta-cell data. Surprisingly, we demonstrate that GPR44/DP2 antagonism do not acutely improve insulin secretion in T2DM patients irrespective of the endogenous incretin response, *i*.*e*. neither during MMTT nor GGI. Furthermore, the GPR44/DP2 antagonist AZD1981 had no significant effect on the paracetamol profile and thus gastric emptying in T2DM patients, which could have confounded the interpretation of MMTT data. At a dose of 200 mg per day for three days, AZD1981 was safe and well tolerated in T2DM patients treated with metformin. The AZD1981 PK profile was adequate and in line with previous reports, and thus, allowed for a clinically and therapeutically relevant testing of the hypothesis. Thereby, we cannot conclude that the PGD_2_-GPR44/DP2 axis is a major therapeutically relevant negative modulator of insulin secretion in T2DM patients.

The patient population recruited to the study was adequately representative of a therapeutically relevant target population e.g. patients with T2DM who have failed to achieve glycemic control on first line standard of care (oral metformin with a balanced gender, ethnic, BMI, and HbA1c distribution. However, it is noteworthy that the study population presented with comparatively long-standing disease as indicated by 9.5 years disease duration and HbA1c of 79 mmol/mol. In line with that, there are several typical pathophysiological features that accompany extended T2DM duration such as the observed glycemic profile during MMTT and the comparatively delayed gastric emptying as demonstrated by the delayed C_max_ observed in our study (See Fig F in S1 File and, for comparison, see Fig 2 in [24]). A potential limitation in the clinical study might be the chosen insulinotropic detection level of 8% in MMTT AUC_Glc 0-4h_ for which the study was powered (N = 20 evaluable subjects for 80% power at the 5% level). The 8%change in MMTT AUC_Glc 0-4h_ is the observed GSIS effect by DPPIV inhibitors [19]. It can be debated if this detection level was set too high given that we did not observe any effects. However, given that DPPIV inhibitors have comparatively modest chronic glycemic efficacy in T2DM, averaging 0.3–0.5% HbA1c reduction, when detecting and validating a novel insulin secretory regulatory pathway of broad clinical relevance and interest we found it hard to argue for a lower efficacy threshold.

A protocol deviation with GLP1 entrapment due to a microfilter incompatibility led to a less than planned GLP1 exposure during the GGI PD test and affected the ability to assess potential effects of GPR44/DP2 antagonism on GLP1 responsiveness during the GGI test. However, it was still possible to draw overall conclusions on GLP1 responsiveness due to the complete lack of effect on ISR after both the MMTT and GGI. The rationale for this is that we observed the expected increase in endogenous GLP1 during the MMTT but it did not result in increased insulinotropic action. This conclusion is further supported by the observation that the endogenous total GLP1 concentration after the MMTT was only about one third lower than the expected GLP1 exposure during the GGI/GLP1 PD test, and furthermore, by the knowledge that the GLP1-insulin secretion dose response relationship in T2DM is linear [25].

The underlying hypothesis for the clinical study, *i*.*e*. the insulinotropic action of GPR44/DP2 antagonism *in vitro*, was based on our data demonstrating that AZD1981 restores PGD_2_-inhibited GSIS by potentiation of intracellular cAMP. That is, we observed that a PGD_2_-analogue acutely reduces GSIS in the human beta-cell line EndoC-betaH1 through inhibition of cAMP. Moreover, high glucose with or without IL-1beta induced significant PGD_2_ production in human islets, as previously seen in rodent islets [11]. Therefore, we hypothesized that the PGD_2_ tone in islets is high in diabetics and that GPR44/DP2 antagonism consequently would improve the insulin response to glucose in humans with T2DM. However, irrespective of endogenous GLP1 secretion after both MMTT and GGI, AZD1981 for 3 days did not have any significant acute insulinotropic efficacy, and we concluded that the *in vitro* data did not translate to a therapeutically relevant *in vivo* disease setting. The possible reasons underlying the lack of translatability are speculative, but the primary hypotheses are [1] an absence of a PGD_2_ tone in T2DM islets *in vivo* and/or [2] few/absent GPR44/DP2 receptors in T2DM islets. We analyzed metabolites for PGD_2_ both in plasma and in urine but did not find any correlation between insulinotropic effects and PGD_2_ metabolite levels. Unfortunately, PGD_2_ has a very short half-life [26] and it is produced in multiple cell types and tissues [27]. Since islets comprise only a very small portion of the total cell pool that produce PGD_2,_ it is likely that global PGD_2_ metabolite concentrations do not accurately reflect the PGD_2_ production in islets. The lack of specific islet PDG_2_ metabolites makes it impossible to conclude if a low islet PGD_2_ tone was the reason for lack of efficacy. Obviously, it was not possible to measure GPR44/DP2 expression in the islets of the trial subjects. However, gene expression data from isolated islets do not support downregulation of GPR44/DP2 in diabetes (see Fig 2) and previously published data comparing GPR44/DP2 protein expression demonstrate no distinct difference between healthy and T2DM islets [8]. GPCR activity is, however, dependent on protein expression on the cell surface, and it is possible that long term diabetes leads to reduced GPR44/DP2 activity via receptor internalization or other mechanisms, which could have contributed to the poor *in vitro-in vivo* translation. In contrast to traditional insulin secretagogues, which have all demonstrated excellent *in vitro*-*in vivo* translation, the GPR44/DP2 mechanism depends on antagonism of a presumed negative feed-back loop. If this feed-back loop is not present in diabetics the antagonistic action of AZD1981 would have no acute effect, which seems to be the case.

This study demonstrates the complexity of islet function *in vivo*, where many different GPCRs work in concert to modulate insulin secretion to match the demand and achieve glucose control. The role of prostaglandins for beta-cell function was recently reviewed [23], further illustrating the complexity of prostaglandin signaling through different pathways that modulate beta-cell function, both stimulating and inhibiting insulin secretion. It has long been known that the enzymes needed for prostaglandin synthesis are expressed in rodent islets and that prostaglandins are produced in response to high glucose and IL-1beta [12, 13]. Also in human islets, activation of the prostaglandin synthesis pathway has been observed by increased expression of COX-2 after treatment with cytokines and high glucose [28, 29]. We confirm this mechanism in intact human islet preparations. However, by mining a human islet single cell sequencing data set [16] we found, to our surprise, that the prostaglandin machinery in human islets is not expressed in endocrine cells but in the stellate cells. This is a novel finding and is not in line with the current consensus for prostaglandin synthesis in islets [28]. To our knowledge the expression pattern has so far only been studied in whole islet preparations and this is the first-time single cell sequencing of human islet has been utilized, allowing analysis of the expression of genes at the cell level. The pancreatic stellate cells are few and quiescent under normal conditions and they surround the acinar, ductal and peri-islet areas [30]. The single cell sequencing data clearly demonstrate that stellate cells are present in human islet preparations [16]. During development of pancreatitis and pancreatic cancer, the stellate cells activate into a myofibroblast phenotype, deteriorating the tissue by rapid growth and secretion of cytokines [31, 32]. Recent studies in animal models have demonstrated activation of stellate cells during development of islet fibrosis and beta-cell dysfunction in T2DM [33, 34], which we confirm, albeit with a small number of cells. The role of stellate cells in the development of human T2DM is currently not known and it could only be speculated that inhibition of PGD_2_ signaling through GPR44/DP2 may require longer treatment periods than performed in the present study to alter beta-cell function and have an impact on glycemic control.

## Supporting information

S1 File Supplementary material vs 11.docx: **Fig A. Expression of PGD**_**2**_**-related genes in all human islet endocrine cell types.** GPR44 (a), DP1 (b), L-PGDS (c), COX1 (d), and COX2 (e). Alpha-cell, n = 886. Beta-cell, n = 309. Delta-cells, n = 114. PP-cells, n = 197. **Fig B. Gene expression of PGD**_**2**_ **receptors in rodent and human islets and beta cells.** (a) The mRNA expression of GPR44 in rodent islet and beta cell lines were significantly lower compared to human islet and beta cells. (b) mRNA expression of GPR44 and DP1 in rat and human primary islets shows almost undetectable levels of DP1 in human islets. The data were normalized against the expression of the housekeeping gene HPRT for each sample. **Fig C. PGD**_**2**_ **potently activates signalling in human beta cells.** The natural ligand for GPR44 is PGD_2_. Since PGD_2_ is unstable in water solution, 15(R)-15-methyl PGD_2_ was used in all experiments. The potency for 15(R)-15-methyl PGD_2_ at the GPR44 receptor was determined using DMR, cAMP and insulin assays. The agonist showed strong potency and good correlation between the assays. A) The DMR signal (Epic) for the PGD_2_ effect on human beta cells (Endo-βH1) (EC_50_ = 0.8x10^-10^M), B) the intracellular cAMP levels (EC_50_ = 0.6x10^-10^M) and C) effects on glucose-stimulated insulin secretion (EC_50_ = 1.2x10^-10^M) were determined. The curves illustrate one representative experiment for each method. Number of data points/assay were for cAMP and insulin assays 6-plicates and 3-plicates for DMR (Epic). Values shown are mean±SEM. **Fig D. Signaling pathway for GPR44 in human beta cells.** GPCRs couple to different G-proteins and it is known that GPR44 couple to the G_αi_ pathway, inhibiting cAMP levels. To establish the signaling pathway in human beta cells, pertussis toxin (PTX) was added to inhibit signaling through G_αi_. Using the DMR assay, PGD_2_ induced a potent effect on the human EndoC-beta cells, while addition of PTX completely blocked the total cell response. This indicate that the major signaling pathway for GPR44 in human beta cells occur through the G_αi_ pathway. **Fig E. AZD1981 dose-response in human beta cell-line (Endo-βH1).** AZD1981 in different concentrations induced no significant agonist response in human beta cells while 15(R)-15-methyl-PGD_2_, which was used as positive control, produced a dose response curve, demonstrating the presence of active GPR44 receptors on the cells. **Fig F. Graded Glucose Infusion in T2DM patients.** C-peptide assessments at baseline (A), after 3 days treatment (B) or Insulin Secretion Rate (ISR) at baseline (C) and after 3 days treatment. Data are presented as mean ± SD. **Fig G. Mixed meal test in T2DM patients with plasma paracetamol assessments after 3 days treatment of AZD1981 or placebo.** Data are presented as mean ± SD. **Fig H. PK/PD analysis of AZD1981 exposure vs selected PD variable after 3 days treatment.** Mixed Meal Tolerance Test and AUC _Glucose (0-4h)_ (A) and for Graded Glucose Infusion and AUC _C-peptide (0-1h)_ (B) in T2DM patients. Data are presented as linear regression. **Fig I. Analysis of exploratory surrogate biomarkers of PGD**_**2**_ **vs AZD1981 PD response eg MMTT AUC**_**Glc (0-4h)**_ **after 3 days treatment.** Plasma 11-beta-PGF_2_-alpha (A) Urine 11-beta-PGF_2_-alpha /creatinine (B) Urine tetranor-PGDM / creatinine (C) and plasma L-PGDS (D) in T2DM patients. Data are presented as linear regression. **Table A. Safety evaluation: Adverse events / Serious Adverse events Table. B. Safety evaluation: Clinical chemistry. Table C. Maximum Plasma AZD1981 Concentration at Steady-State, Css,Max**
(DOCX)

S2 File CONSORT 2010 checklist. (DOC)

S3 File D6420C00001 clinical study protocol final. (PDF)
