# Supplementary material for: Exploring the insulin secretory properties of the PGD2-GPR44/DP2 axis in vitro and in a randomized phase-1 trial of type 2 diabetes patients
Source: PLoS One. 2018 Dec 17;13(12):e0208998. doi: 10.1371/journal.pone.0208998 (PMC6296667; doi:10.1371/journal.pone.0208998)
Supplement: S1 File — Supplementary material vs 11.docx: Fig A. Expression of PGD2-related genes in all human islet endocrine cell types. GPR44 (a), DP1 (b), L-PGDS (c), COX1 (d), and COX2 (e). Alpha-cell, n = 886. Beta-cell, n = 309. Delta-cells, n = 114. PP-cells, n = 197. Fig B. Gene expression of PGD2 receptors in rodent and human islets and beta cells. (a) The mRNA expression of GPR44 in rodent islet and beta cell lines were significantly lower compared to human islet and beta cells. (b) mRNA expression of GPR44 and DP1 in rat and human primary islets shows almost undetectable levels of DP1 in human islets. The data were normalized against the expression of the housekeeping gene HPRT for each sample. Fig C. PGD2 potently activates signalling in human beta cells. The natural ligand for GPR44 is PGD2. Since PGD2 is unstable in water solution, 15(R)-15-methyl PGD2 was used in all experiments. The potency for 15(R)-15-methyl PGD2 at the GPR44 receptor was determined using DMR, cAMP and insulin assays. The agonist showed strong potency and good correlation between the assays. A) The DMR signal (Epic) for the PGD2 effect on human beta cells (Endo-βH1) (EC50 = 0.8x10^-10^M), B) the intracellular cAMP levels (EC50 = 0.6x10^-10^M) and C) effects on glucose-stimulated insulin secretion (EC50 = 1.2x10^-10^M) were determined. The curves illustrate one representative experiment for each method. Number of data points/assay were for cAMP and insulin assays 6-plicates and 3-plicates for DMR (Epic). Values shown are mean±SEM. Fig D. Signaling pathway for GPR44 in human beta cells. GPCRs couple to different G-proteins and it is known that GPR44 couple to the Gαi pathway, inhibiting cAMP levels. To establish the signaling pathway in human beta cells, pertussis toxin (PTX) was added to inhibit signaling through Gαi. Using the DMR assay, PGD2 induced a potent effect on the human EndoC-beta cells, while addition of PTX completely blocked the total cell response. This indicate that the major signaling pathway f [file pone.0208998.s001.docx]

**Supplementary material**

Table of Contents

[Material & Methods 2](#_Toc500966022)

[DMR assay – label free receptor assay 2](#_Toc500966023)

[cAMP measurements 2](#_Toc500966024)

[Insulin secretion 2](#_Toc500966025)

[PGD_2_ secretion from human islets 3](#_Toc500966026)

[Expression of GPR44 and components in the PGD2 synthesis pathway in human islets 3](#_Toc500966027)

[Study design 4](#_Toc500966028)

[Pharmacokinetic and pharmacodynamic in clinical study 4](#_Toc500966029)

[PD analysis of exploratory surrogate biomarkers of PGD_2_ tone 5](#_Toc500966030)

[AZD1981 demonstrate no internal agonist activity 9](#_Toc500966031)

[Effects on C-peptide after GGI in T2DM patients 11](#_Toc500966032)

[Effects on paracetamol pharmacokinetics after MMTT in T2DM patients 12](#_Toc500966033)

[PK/PD analysis after MMTT & GGI in T2DM patients 13](#_Toc500966034)

[Analysis of exploratory surrogate biomarkers of PGD_2_ tone vs PD response 14](#_Toc500966035)

[ESM Table 1. Potency of AZD1981 in inhibition of cellular responses mediated by GPR44 15](#_Toc500966036)

[ESM Table 2. Safety evalution: Adverse events / Serious Adverse events 16](#_Toc500966037)

[ESM Table 3. Safety evaluation: Clinical chemistry 17](#_Toc500966038)

## Material & Methods

### DMR assay – label free receptor assay

The Epic® label-free screening system (Corning®) measures the dynamic mass redistribution (DMR) of cells, which enables real-time detection of integrated cellular responses in living cells. It relies on detection of refractive index alterations on biosensor-coated microplates that originate from changes in the total biomass proximal to the sensor surface in response to a stimulus such as activation of a G-protein coupled receptor. This technique can be used to confirm the activity of ligands at cell surface receptors and to explore the signaling pathways [19].

Human EndoC-βH1 cells were plated at a density of 2x10^4^ cells/well in 384-well fibronectin-coated Epic biosensor plates (Corning) and cultured at 37°C 5% CO_2_ for 24 h. On the day of experiment the cells were washed with assay buffer (1xHBSS, 20 mM HEPES (pH 7.4) and 0.2 % BSA) and allowed to equilibrate for 1h inside the Corning Epic Biosensor at 26°C. Following equilibration, a 5 min scan was performed to create a baseline read before applying AZD1981 at a concentration range of 1x10^-5^M to 3.8x10^-11^M diluted in assay buffer containing 150 pM 15(R)-15-methyl Prostaglandin D_2_ using a CyBi-Well vario. The real-time measurement of DMR was detected during a 60 min scan. For the signaling pathway studies Human EndoC-βH1 cells were treated with 100 ng/ml of PTX (Pertussis toxin), 24 h prior to activation with AZD1981 and the real-time measurement of DMR.

### cAMP measurements

Human EndoC-βH1 cells were plated in fibronectin- and extracellular matrix-coated 96-well plates at a density of 5x10^4^ cells per well and cultured for 2 days at 37°C, 5% CO_2_. The maintenance medium was then replaced with low glucose (2.8mM) medium and cells were cultured for another 16 h. Before stimulation of cAMP production, a 1 h pre-incubation in KREBS buffer with 0.2% BSA and 0.5mM glucose was performed at 37°C, 5% CO_2_. Cyclic AMP experiments were then performed for 30 minutes at 37°C, 5% CO_2_ by incubating cells in KREBS buffer containing 0.2% BSA, 0.5mM 3-isobutyl-1-methyl-xanthine (IBMX, a phosphodiesterase inhibitor) and either 2.8 or 11.1mM glucose, in the presence of serial dilutions of AZD1981 at a concentration range of 3.81x10^-11^M to 2.5 x10^-6^M, with or without EC_80_ (150 pM) of the GPR44 agonist 15(R)-15-methyl Prostaglandin D_2_. Exendin-4 was used as a reference compound, well-known to stimulate insulin secretion via increased cAMP production.

At the end of the incubation period, cells were lysed, and cAMP levels in the cell lysate was measured using an HTRF®-based cAMP assay from Cisbio Bioassays and read on a Paradigm plate reader (Molecular Devices) at 665 and 620nm. cAMP standard curve was prepared according to the Cisbio protocol.

### Insulin secretion

Human EndoC-βH1 cells were plated and pre-treated as described above for the cAMP measurements. GSIS experiments were then performed for 1hr at 37°C, 5% CO_2_ by incubating cells in KREBS buffer containing 0.2% BSA, 0.5mM 3-isobutyl-1-methyl-xanthine (IBMX, a phosphodiesterase inhibitor) and either 2.8 or 11.1mM glucose, in the presence of serial dilutions of AZD1981 at a concentration range of 3.81x10^-11^M to 2.5 x10^-6^M, with or without EC_80_ of the GPR44 agonist 15(R)-15-methyl Prostaglandin D_2_. Exendin-4 was used as a reference compound.

At the end of the incubation period, cell-culture supernatants were collected and insulin levels determined by an HTRF®-based insulin assay from Cisbio Bioassays and read on a Paradigm plate reader (Molecular Devices) at 665 and 620nm. Insulin standard curve was prepared according to the Cisbio protocol.

In other experiments, EndoC cells were incubated with 15(R)-15-methyl-PGD_2_ at EC_80_ (150 pM) at 11.1 mM glucose with and without addition of 100 nM exendin-4 (Bachem AG, CH), a GLP-1 receptor agonist. The effect of AZD1981 on insulin secretion was explored at high glucose with 15(R)-15-methyl-PGD_2_. After 30 min incubation, insulin secretion was measured as described above.

### PGD_2_ secretion from human islets

Human islets (Prodo Laboratories Inc USA) were used for measurement of PGD_2_ secretion and expression of enzymes in the PGD_2_ synthesis pathway. Islets were shipped over night in transportation media (PIMT®, Prodo Laboratories Inc) and cultured for one days to allow recovery. Approximately 2000 human islet equivalents were divided and transferred into 24-well plates with complete media (PIMS®, Prodo Labs) at 5.6 mM glucose, 22.2 mM glucose or 22.2 mM glucose and 20 ng/ml IL-1β (BD Biosciences, San Diego, USA). The islets were incubated at 37^o^C, at 5% CO_2_ for 24h. A sample of the media was taken from each well and stored at -80^o^C for analysis of PGD_2_ (PGD_2_ EIA-kit, Cayman). After another 24h incubation, a new media sample was collected from each well and the islets were harvested and stored at -80^o^C before analysis of gene expression.

### Expression of GPR44 and components in the PGD_2_ synthesis pathway in human islets

The expression of genes in the GPR44 and PGD_2_ synthesis pathway was measured using qPCR in primary human islets and by reanalyzing published in house human islet single cell sequencing data [16]. Baron et al. [18] previously identified three subtypes of stellate cells in a large dataset of 457 human stellate cells (which included 1 TD2 donor). We used the cell type markers identified in this study to perform average linkage hierarchical clustering on 54 stellate cells in the in-house dataset [16] (which contained 4 T2DM donors). The clustering was based on the cosine distance between the log2 transformed transcriptomes reduced to the 20 cell type markers only. Clusters were identified by visual inspection of the dendrogram branches. Enrichment for quiescent, ‘standard activated’ and ‘immune-activated’ stellate cells in TD2 was calculated based on Fisher’s exact test (one-tailed). Differential expression between the different states of activation was investigated with Non-parametric one-way ANOVA (Kruskal-Wallis test).

For qPCR, total RNA was extracted from the islets that were treated with low or high glucose and cytokines as described above, using RNeasy MiniKit (Qiagen Inc, USA). Complementary DNA (cDNA) was generated using the High-Capacity cDNA reverse transcription kit (Applied Biosystems) and RT-qPCR analysis was performed with an ABI Prism 7900 (Applied Biosystems Inc, USA) using Taqman Gene expression assays (list of genes in Supplementary Table 1, Applied Biosystems) following manufacturer’s instructions. The data was normalized against acidic ribosomal phosphoprotein P0 (m36B4) expression for each sample.

**Supplementary Table.** Primer sequence of the analyzed human genes

| **Gene name** | **Protein** | **Primer** |
| --- | --- | --- |
| PTGDR2 | GPR44 | Hs01867513_s1 |
| PTGDR | DP1 | Hs00235003_m1 |
| PTGDS | L-PGDS | Hs00168748_m1 |
| PTGS1 | Cox1 | Hs00377726_m1 |
| PTGS2 | Cox 2 | Hs00153133_m1 |

### Study design

The primary purpose was to increase the human target validation of GPR44 antagonism as a novel anti-diabetic treatment modality as well as to explore the glycemic mechanism of action (MoA) of GPR44 antagonism by using AZD1981 as a tool compound given po 100 mg BID for 3 days. The pharmacokinetic (PK) and pharmacodynamic (PD) effects of AZD1981 were determined relative to placebo. Patients were randomly assigned (ratio 1:1), using consecutive randomisation codes, to receive treatment with either AZD1981 or placebo first.

In more detail, after screening, twenty metformin treated Type 2 Diabetes (T2DM) subjects with inadequate glycemic control (HbA1c) were randomized and recruited. After study inclusion they went through a run-in period of at least 14 days. At day -2 they were admitted for physical examination including vital signs and safety laboratory. After assessment of BMR individualized weight maintaining diet was put in place through-out the residential stay, considering body weight, body composition, gender, dietary habits as planned by a dietist/nutritionist upon first admission at day -2. At day -1, after an over-night fast, the baseline pharmacodynamic assessment for the first study period were done. The subjects were also further put on an individualised weight neutral diet from day-1. At day 3 another pharmacodynamic assessment was performed. Day 4 & 5 were wash-out days and at day 6 another pharmacodynamic assessment (baseline for 2^nd^ study period) was performed. At day 9 the final pharmacodynamic assessment was done. The subjects were then discharged and returned for a follow up visit 7-14 days later for physical examination including vital signs, safety laboratory tests and AE reporting.

A dose of 200 mg (100mg twice a day) for three days was used for the PoM study as:

• Steady state are achieved after 1 day

• Cmin 3-fold greater than the free DMR EPIC potency binding data (EC50 of 1.3nM)

• Adequate safety margin versus the observed LFT effects at >800 mg/day

• Adequate safety margin versus significant DDI at doses >800 mg/day

### Pharmacokinetic and pharmacodynamic in clinical study

Two cannulas were inserted (one in each forearm) following the (optional) application of topical anesthetic cream. These were kept patent by slowly running saline drip. Two baseline blood samples were taken, at 15 min prior to the Ensure Plus drink and at 1 min prior to the Ensure Plus drink. At time 0 min, the patient were asked to drink Ensure Plus drink (472 ml, 16 Oz, 100g CHO) over a period of 2-5 min. Blood samples were taken at 15, 30, 60, 90, and 120 post the Ensure Plus.

Identical study procedures and subject restriction were undertaken for the second pharmacodynamics assessment, e.g. the graded glucose infusion. At time t=0 min a graded glucose infusion was started using 20% glucose following the schedule: 2mg/kg/min for 30 min, 4 mg/kg/min for 30 min, 6 mg/kg/min for 30 min, 8 mg/kg/min for 30 min. Blood samples were taken at 10 min intervals between 130 and 180 min. At time t=180 min a GLP1 infusion was started, with an infusion rate of 2 pmol/min/kg for 10 min then reducing to 1 pmol/min/kg for 50 min. Blood samples were taken at 10 min intervals between 190 and 240 min. However, analysis of total GLP1 during the GLP1 infusion after study completion showed no increase in GLP1 levels. After investigation it was revealed that the GLP1 was entrapped in the infusion kit.

The blood at each time point was placed into an EDTA tube (4 ml) for glucose, C-peptide, and insulin (21 time points in total) and into BD P800 tubes (2ml) at 7 time points for glucagon and total GLP1 assessment.

Paracetamol was used to determine the rate of gastric emptying concurrent with the MMTT on days 3 and 9. Immediately following consumption of the Ensure Plus® drink, a 1000 mg paracetamol solution was consumed.

Blood samples were obtained for assessments of AZD1981 PK on days 2, 3, 8, and 9 concurrent with the MMTT and GGI, and for paracetamol PK on days 3 and 9 during the MMTT. Samples collected during placebo treatment were only analysed for plasma paracetamol, not for AZD1981 unless specified.

EDTA plasma and urine was collected on day -1, 3, 6 and 9 for analysis of exploratory biomarkers performed at AstraZeneca.

### PD analysis of exploratory surrogate biomarkers of PGD_2_ tone

An exploration was made to identify subjects with a high PGD_2_ tone (high local pancreatic levels of PGD_2_) by determining circulating surrogate biomarkers of local PGD2 tone and to correlate them with PD response in order to see if there is a specific responder sub-population. Local cellular PGD_2_ is rapidly metabolized. The PGD_2_ surrogate biomarkers selected for assessment were the major PGD_2_ metabolites 11-beta-PGF_2_-alpha in plasma and urine and tetra-nor-PGDM in urine. As a potential marker of high PGD_2_ synthesis plasma L-PGDS was assessed as well. Analysis was performed in a fully automated Beckman Coulter Motoman robotic system (Including Biomek FX Span-8 and 96-head, Biotek cellwasher, Cytomat Incubators, hotels and MD Paradigm Reader) allowing high-quality data in short time.

11-beta-PGF_2_-alpha was analyzed in undiluted plasma and 1:10 diluted urine using the Cayman EIA-kit (cat no 516521) with a calibration curve range of 1.6 - 1000 pg/mL. In-house QC sample were prepared from pooled human plasma samples.

Tetranor-PGDM (a PGD_2_ metabolite) was analyzed in 1:25 diluted urine using the Cayman EIA-kit (cat no 501001) with a calibration curve range of 6.6 - 4000 pg/mL. In-house QC sample were prepared from pooled human urine samples.

Prostaglandin D Synthase (Lipocalin-type) (L-PGDS) was analyzed in 1:100 diluted plasma using the BioVendor ELISA (cat no RD191113100R). Samples were diluted 1:100 in the final assay. The calibration curve range was 1 - 40 ng/mL. In-house QC samples were prepared from pooled human plasma.

Urinary creatinine was analyzed in 1:10 diluted urine using the Horiba ABX Creatinine 120CP kit (cat no A11A01933) set up for automation on the Pentra 400 instrument. Samples were diluted 1:10 prior to analysis. The calibration curve range was 20 - 3500 µM. In-house QC samples were prepared from pooled human urine.


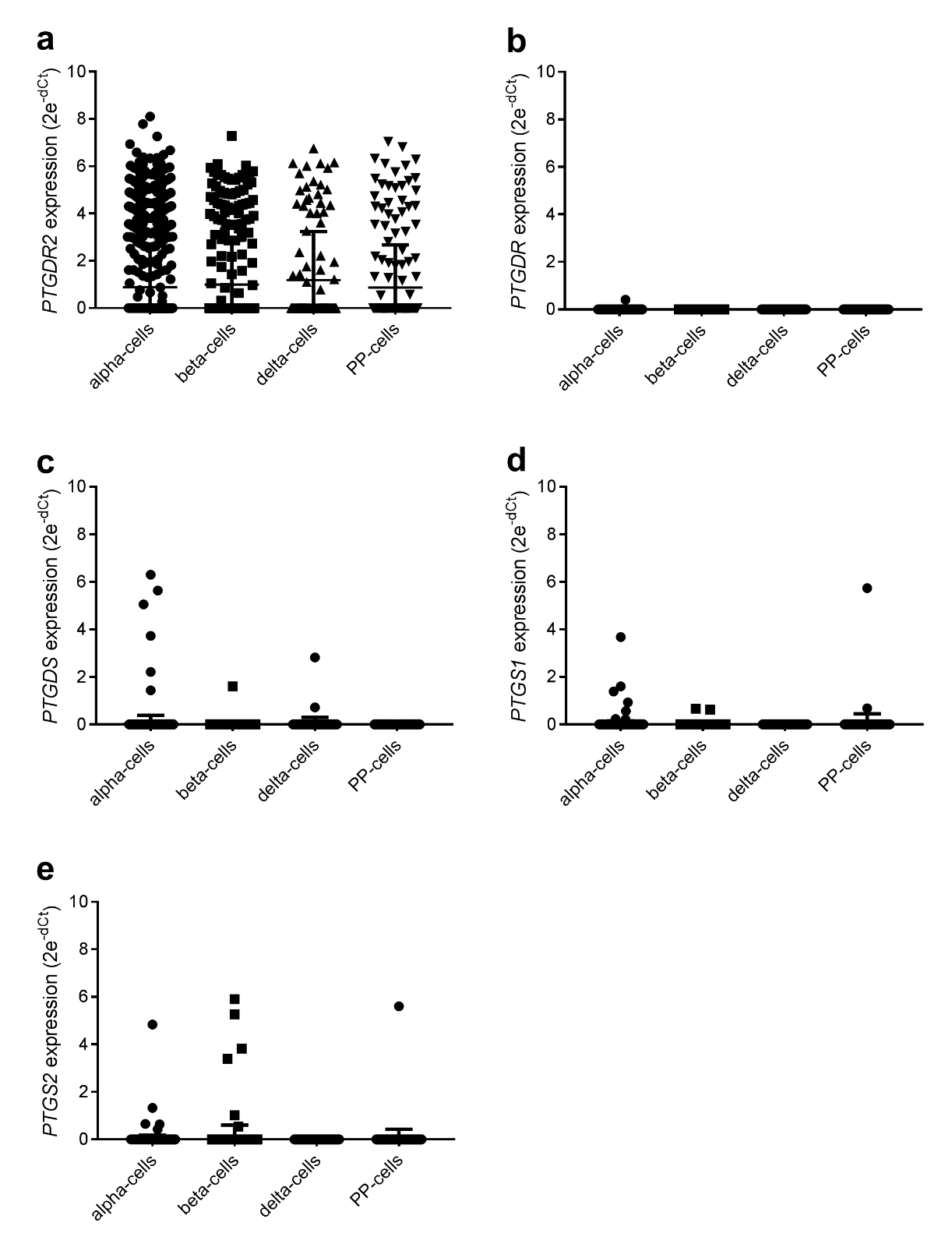


**Fig A. Expression of PGD_2_-related genes in all human islet endocrine cell types.** GPR44 (a), DP1 (b), L-PGDS (c), COX1 (d), and COX2 (e). Alpha-cell, n=886. Beta-cell, n=309. Delta-cells, n=114. PP-cells, n=197.


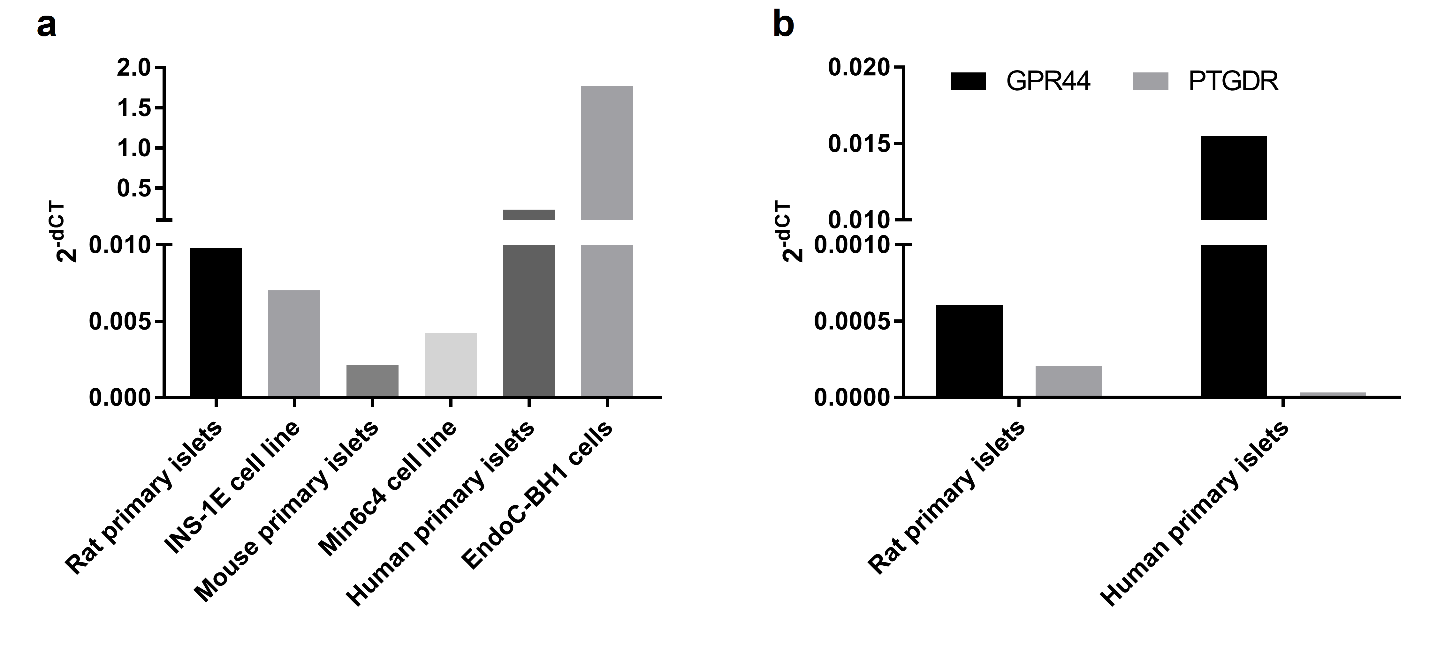


**Fig B. Gene expression of PGD_2_ receptors in rodent and human islets and beta cells** (a) The mRNA expression of GPR44 in rodent islet and beta cell lines were significantly lower compared to human islet and beta cells. (b) mRNA expression of GPR44 and DP1 in rat and human primary islets shows almost undetectable levels of DP1 in human islets. The data were normalized against the expression of the housekeeping gene HPRT for each sample.


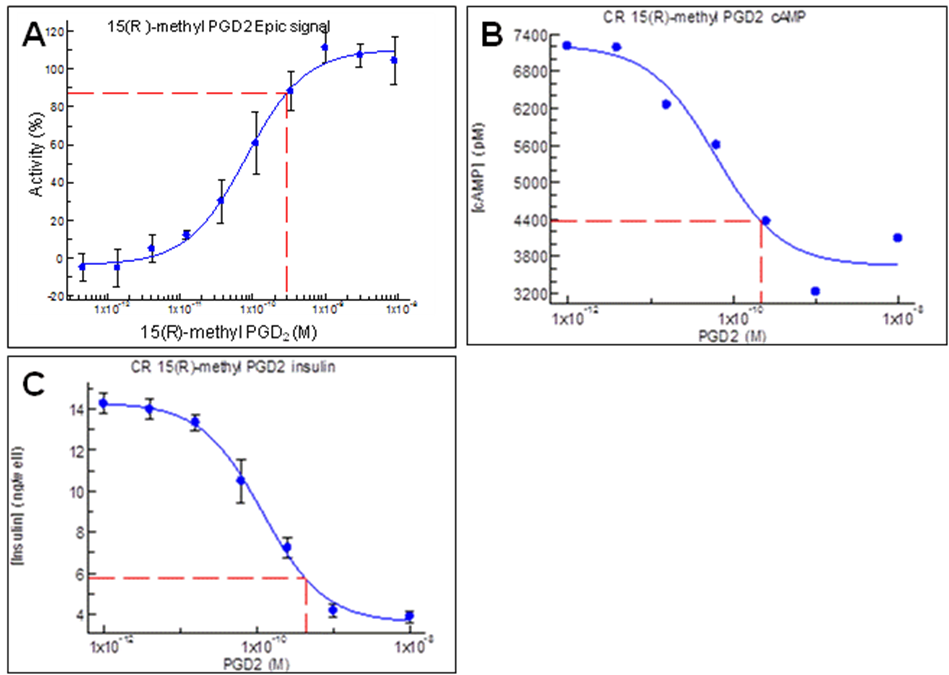


**Fig C. PGD_2_ potently activates signalling in human beta cells.** The natural ligand for GPR44 is PGD_2_. Since PGD_2_ is unstable in water solution, 15(R)-15-methyl PGD_2_ was used in all experiments. The potency for 15(R)-15-methyl PGD_2_ at the GPR44 receptor was determined using DMR, cAMP and insulin assays. The agonist showed strong potency and good correlation between the assays. A) The DMR signal (Epic) for the PGD_2_ effect on human beta cells (Endo-βH1) (EC_50_= 0.8x10^-10^M), B) the intracellular cAMP levels (EC_50_= 0.6x10^-10^M) and C) effects on glucose-stimulated insulin secretion (EC_50_= 1.2x10^-10^M) were determined. The curves illustrate one representative experiment for each method. Number of data points/assay were for cAMP and insulin assays 6-plicates and 3-plicates for DMR (Epic). Values shown are mean±SEM.


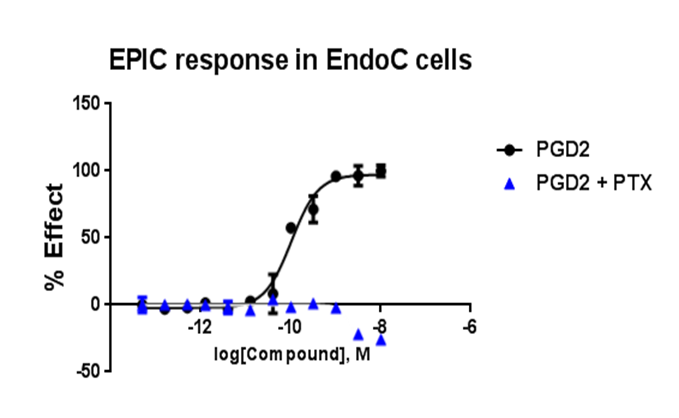


**Fig D. Signaling pathway for GPR44 in human beta cells.** GPCRs couple to different G-proteins and it is known that GPR44 couple to the G_αi_ pathway, inhibiting cAMP levels. To establish the signaling pathway in human beta cells, pertussis toxin (PTX) was added to inhibit signaling through G_αi_. Using the DMR assay, PGD_2_ induced a potent effect on the human EndoC-beta cells, while addition of PTX completely blocked the total cell response. This indicate that the major signaling pathway for GPR44 in human beta cells occur through the G_αi_ pathway.

### AZD1981 demonstrate no internal agonist activity

The effect of AZD1981 on the total cell response (DMR; Coring Epic biosensor) was tested without addition of 15(R)-15-methyl-PGD_2_ to establish if the compound has an internal agonist activity. Human beta cells (Endo-βH1) were incubated with different concentrations of AZD1981 and the total cell response was recorded. 15(R)-15-methyl-PGD_2_ was added to other cells to represent a positive control demonstrating a potent effect on GPR44 activation. No agonist effect was detected with AD1981 in concentrations up to 1µM.


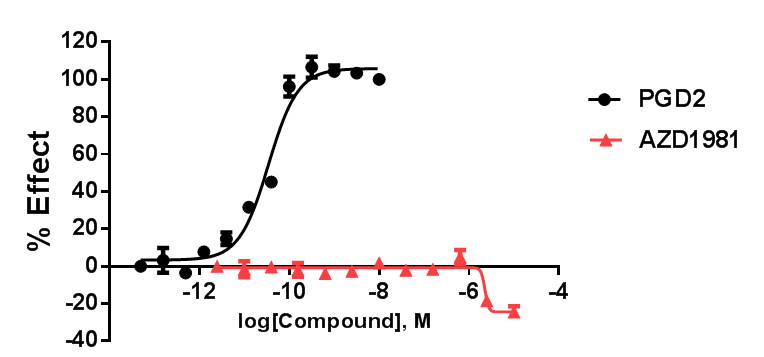


**Fig E.** **AZD1981 dose-response in human beta cell-line (Endo-βH1).** AZD1981 in different concentrations induced no significant agonist response in human beta cells while 15(R)-15-methyl-PGD_2_, which was used as positive control, produced a dose response curve, demonstrating the presence of active GPR44 receptors on the cells.

### Effects on C-peptide after GGI in T2DM patients


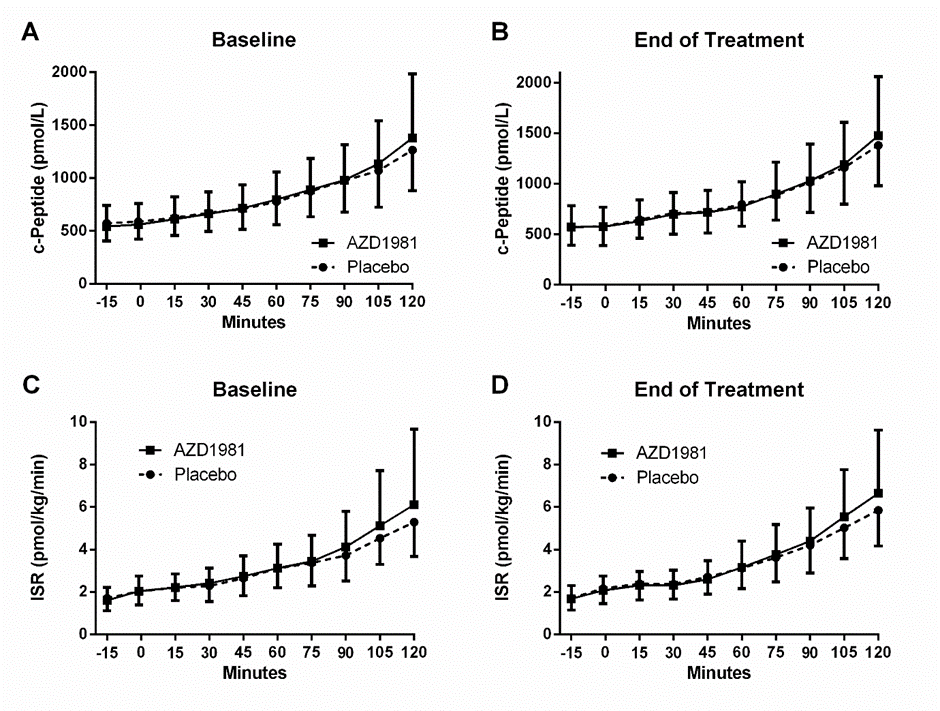


**Fig F. Graded Glucose Infusion in T2DM patients.** C-peptide assessments at baseline (A), after 3 days treatment (B) or Insulin Secretion Rate (ISR) at baseline (C) and after 3 days treatment. Data are presented as mean ± SD.

### Effects on paracetamol pharmacokinetics after MMTT in T2DM patients





**Fig G. Mixed meal test in T2DM patients with plasma paracetamol assessments after 3 days treatment of AZD1981 or placebo.** Data are presented as mean ± SD.

### PK/PD analysis after MMTT & GGI in T2DM patients

**

**

**Fig H. PK/PD analysis of AZD1981 exposure vs selected PD variable after 3 days treatment.** Mixed Meal Tolerance Test and AUC _Glucose (0-4h)_ (A) and for Graded Glucose Infusion and AUC _C-peptide (0-1h)_ (B) in T2DM patients. Data are presented as linear regression.

### Analysis of exploratory surrogate biomarkers of PGD_2_ tone vs PD response


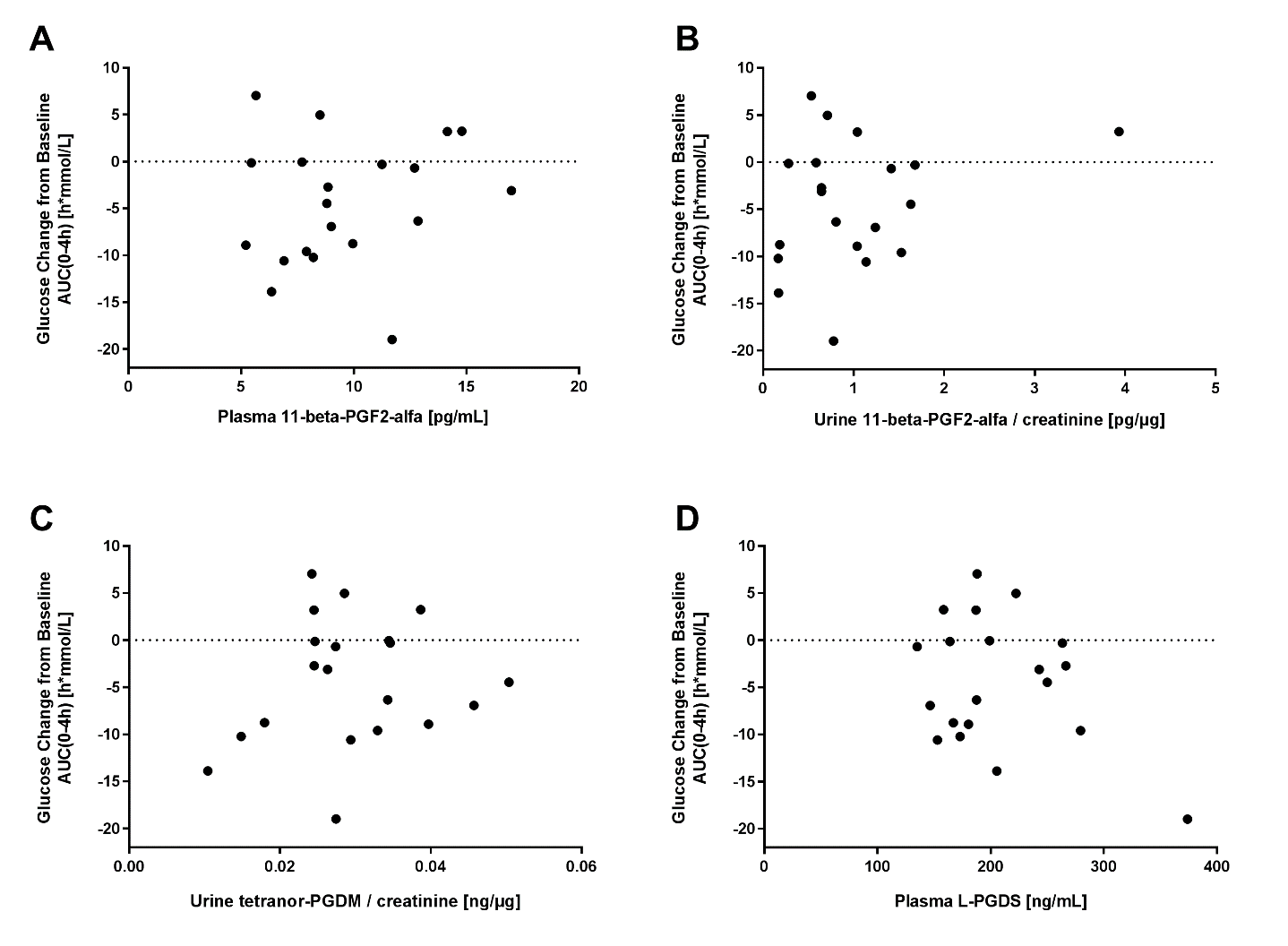


**Fig I. Analysis of exploratory surrogate biomarkers of PGD_2_ vs AZD1981 PD response eg MMTT AUC _Glc (0-4h)_ after 3 days treatment.** Plasma 11-beta-PGF_2_-alpha (A) Urine 11-beta-PGF_2_-alpha /creatinine (B) Urine tetranor-PGDM / creatinine (C) and plasma L-PGDS (D) in T2DM patients. Data are presented as linear regression.

### Table A. Safety evaluation: Adverse events / Serious Adverse events

| Summary of TEAEs by Treatment- Safety Population | | |
| --- | --- | --- |
| Treatment-emergent adverse events (TEAE) | Treatment | |
| System Organ Class, n (%)  Preferred term, n (%) | AZD1981 (n=20) | Placebo (n=20) |
| **Number of patients with any TEAE** | 5 (25.00) | 6 (30.00) |
| **Blood and lymphatic system disorders** | 0 (0.00) | 1 (5.00) |
| Anaemia | 0 (0.00) | 1 (5.00) |
| **Gastrointestinal disorders** | 3 (15.00) | 4 (20.00) |
| Diarrhoea | 3 (15.00) | 2 (10.00) |
| Abdominal distension | 0 (0.00) | 1 (5.00) |
| Abdominal pain | 0 (0.00) | 1 (5.00) |
| Frequent bowel movements | 0 (0.00) | 1 (5.00) |
| Nausea | 0 (0.00) | 1 (5.00) |
| Vomiting | 0 (0.00) | 1 (5.00) |
| **General disorders and administration site conditions** | 2 (10.00) | 4 (20.00) |
| Vessel puncture site haemorrhage | 2 (10.00) | 2 (10.00) |
| Local swelling | 0 (0.00) | 1 (5.00) |
| Vessel puncture site reaction | 0 (0.00) | 1 (5.00) |
| **Psychiatric disorders** | 1 (5.00) | 1 (5.00) |
| Insomnia | 1 (5.00) | 1 (5.00) |
| **Skin and subcutaneous tissue disorders** | 0 (0.00) | 1 (5.00) |
| Dermatitis contact | 0 (0.00) | 1 (5.00) |

### Table B. Safety evaluation: Clinical chemistry

| Mean (SD) Change from Baseline for all Clinical Chemistry Parameters | | | | | | |
| --- | --- | --- | --- | --- | --- | --- |
| Laboratory Test (Unit) | AZD1981-Placebo (n=10) | | | Placebo-AZD1981 (n=10) | | |
|  | Day 3 | Day 9 | Follow-up | Day 3 | Day 9 | Follow-up |
| Alkaline Phosphatase (IU/l) | -7.10 (9.62) | -9.70 (8.88) | 7.40 (12.62) | -10.20 (9.31) | -11.00 (8.78) | 2.00 (7.32) |
| Alanine Aminotransferase (IU/l) | 1.40 (5.36) | -1.30 (4.83) | 10.90 (31.54) | 1.50 (8.34) | -1.50 (5.70) | 1.10 (8.27) |
| Aspartate Aminotransferase (IU/l) | 0.00 (3.62) | 0.00 (3.16) | 1.20 (2.53) | 0.00 (7.04) | -1.40 (4.03) | 1.80 (5.01) |
| Bilirubin (mg/dl) | -0.06 (0.12) | -0.08 (0.11) | -0.04 (0.14) | -0.15 (0.15) | -0.12 (0.18) | -0.04 (0.17) |
| Calcium (mg/dl) | -0.07 (0.35) | -0.10 (0.18) | 0.25 (0.35) | -0.22 (0.46) | -0.16 (0.76) | 0.15 (0.54) |
| Cholesterol (mg/dl) | -13.20 (8.57) | -23.10 (10.03) | 1.40 (13.87) | -8.80 (16.69) | -12.90 (25.83) | 1.40 (13.70) |
| Creatinine | -0.01 (0.11) | 0.00 (0.10) | 0.04 (0.07) | -0.02 (0.10) | -0.04 (0.15) | 0.05 (0.09) |
| C-reactive Protein (mg/l) | -0.03 (1.65) | 0.60 (2.60) | 0.51 (4.31) | -1.93 (4.92) | -2.17 (5.50) | -1.77 (6.55) |
| Free Fatty Acid (mEQ/l) | 0.00 (0.13) | 0.06 (0.18) | -0.05 (0.26) | -0.13 (0.10) | -0.05 (0.15) | 0.02 (0.22) |
| Glomerular Filtration Rate^a^ | 1.40 (8.81) | 0.20 (7.02) | -3.40 (5.30) | 2.10 (8.31) | 4.00 (12.71) | -3.20 (8.30) |
| Potassium (mmol/l) | -0.14 (0.37) | -0.16 (0.26) | -0.01 (0.33) | -0.10 (0.41) | -0.14 (0.32) | 0.12 (0.25) |
| Sodium (mmol/l) | 0.10 (1.97) | 0.00 (1.83) | -0.40 (1.35) | 0.30 (1.57) | 0.40 (1.84) | 0.50 (2.37) |
| Triglycerides (mg/dl) | -1.20 (30.46) | -13.20 (25.53) | 4.80 (25.82) | -7.20 (35.67) | -15.60 (20.32) | 3.90 (20.43) |

### Table C. Maximum Plasma AZD1981 Concentration at Steady-State, Css,Max

|  | **MMTT** | **GGI (Graded Glucose and GLP1 Infusion)** |
| --- | --- | --- |
| **Participants Analyzed** | **20** | **20** |
| **Maximum Plasma AZD1981 Concentration at Steady-State, Css,Max**  [Units: nmol/L] Geometric Mean (Geometric Coefficient of Variation) | **2507   (71.1%)** | **3662   (86.9%)** |
