# Supplementary material for: Exploring the insulin secretory properties of the PGD2-GPR44/DP2 axis in vitro and in a randomized phase-1 trial of type 2 diabetes patients
Source: PLoS One. 2018 Dec 17;13(12):e0208998. doi: 10.1371/journal.pone.0208998 (PMC6296667; doi:10.1371/journal.pone.0208998)
Supplement: S3 File — (PDF) [file pone.0208998.s003.pdf]

**Clinical Study Protocol**	
Drug Substance	AZD1981
Study Code	D6420C00001
Edition Number	1.0
Date	11 Dec 2014

A Single Centre, Double-blind, Randomised, Placebo-controlled, Cross-over Phase I Study to Assess the Pharmacodynamics of oral AZD1981 after Administration of Repeated Doses for 3 days in Subjects with Type 2 Diabetes Mellitus

Sponsor: AstraZenecaAB, S-151 85 Södertälje, Sweden

The following Amendment(s) and Administrative Changes have been made to this protocol since the date of preparation:

Amendment No:	Date of Amendment:	Local Amendment No:	Date of Local Amendment:
**Administrative Change No:**	**Date of Administrative Change:**	**Local Administrative Change No:**	**Date of Local Administrative Change:**

This submission /document contains trade secrets and confidential commercial information, disclosure of which is prohibited without providing advance notice to AstraZeneca and opportunity to object.

PROTOCOL SYNOPSIS

A Single Centre, Double-blind, Randomised, Placebo-controlled, Cross-over Phase I Study to Assess the Pharmacodynamics of oral AZD1981 after Administration of Repeated Doses for 3 days in Subjects with Type 2 Diabetes Mellitus

Principal Investigator

Liliana Uribe-Bruce, MD, MCI
Endocrinology, Diabetes & Metabolism
Phone: +1(619) 409-1277 or (619) 409-1261
Liliana.uribe-bruce@profilinstitute.com

Study centre

Profil™ Institute for Clinical Research, Inc.
855 3rd Avenue, Suite 4400
Chula Vista, CA 91911, U.S.A.

Number of subjects planned

Up to 25 subjects are planned to be randomised/dosed in order to achieve 20 fully evaluable subjects (completed both treatment periods with full primary pharmacodynamic (PD) assessments done).

Study time schedule

Study period		Phase of development
Estimated date of first subject enrolled	Q1 2015	PoM - Human Target Validation
Estimated date of last subject completed	Q2/Q3 2015	

Objectives

Primary Objectives

- To assess the change in plasma glucose after mixed-meal tolerance test (MMTT) compared to baseline
  - Change in glucose concentration-time curve ($AUC_{0\text{-}4\,h}$)
  - Change in glucose maximum plasma concentration (C_{max}) level
- To assess the change in insulin secretion rate by assessment of C-peptide after graded glucose and GLP1 infusion (GGI)

Secondary Objectives

- To assess the change in plasma insulin, C-peptide and glucagon after MMTT compared to baseline
- To assess the change in glucose and insulin after GGI
- To assess the change in 24 h glucose, fasting insulin and beta cell responsiveness after MMTT and GGI
- To assess the pharmacokinetics (PK) of AZD1981 after repeated oral doses
- To assess the change in paracetamol in plasma during MMTT
- To assess safety and tolerability after repeated oral doses of AZD1981 in patients with type 2 diabetes mellitus (T2DM)

Exploratory Objectives

- To assess changes in the primary endpoints based on exploratory patient segmentation biomarkers (plasma and urine PGD2-metabolites, P-SHBG, B-Eosinophils)
- To explore the relationship between AZD1981 exposure and the pharmacodynamic (PD) variables e.g. MMTT AUCPD and GGI AUCPD
- To collect and store plasma samples for potential future exploratory research aimed at exploring biomarkers involved associated with the pharmacological action of G protein-coupled receptor 44 (GPR44) antagonism and the PGD2-GPR44 pathway
- To collect and store blood samples for potential future exploratory genetic research aimed at identifying/exploring genetic variations that may affect pharmacokinetics and pharmacodynamics, safety and tolerability related to GPR44 antagonism and the PGD2-GPR44 pathway (see Appendix D of this Clinical Study Protocol)

Study design
This is a single-centre, double-blind, randomised, placebo-controlled, cross-over study to investigate the effects of repeated doses of AZD1981 on Glucose Stimulated Insulin Secretion (GSIS) in male and female T2DM patients.

Target subject population
Male and female T2DM patients, aged ≥18 years, with poor glycaemic control on metformin (HbA1c ≥7.5 but ≤11%) and BMI ≥19 and ≤38 kg/m^2.

Investigational product, dosage and mode of administration
Each subject will receive an oral dose of AZD1981 (10 tablets of 10 mg) or matching placebo (10 tablets) twice daily (12 hours apart) on Days 1 to 3 of Treatment period 1. The first morning dose on each treatment day will be administered after an overnight fast of at least 10 hours with 240 mL of water. On each treatment day, the 2nd daily dose will be given at

least 1 hour after a meal. The same dosing pattern will be applied during the Treatment period 2 (Days 6 to 9). The last dose in the study will be given on Day 9.

The total daily dose of AZD1981 will be 200 mg.

Duration of treatment
Each subject will receive repeated doses of AZD1981 or placebo for 3 consecutive days in a cross-over design consisting of two treatment periods separated by a washout period of 3 days.

Outcome variable(s):

Safety

- Adverse events, vital signs, electrocardiograms (ECGs), safety laboratory variables

Pharmacokinetics (PK)

AZD1981
The following PK parameters will be determined for AZD1981:

- Maximum plasma concentration at steady state ($C_{ss,max}$), time to steady state C_{max} ($t_{ss,max}$), minimum plasma concentration at steady state ($C_{ss,min}$), apparent oral plasma clearance at steady state (CL_{ss}/F), area under the plasma concentration-time curve during following intervals: 0-1 h ($AUC_{0\text{-}1h}$), 0-2 h ($AUC_{0\text{-}2h}$), 0-4 h ($AUC_{0\text{-}4h}$), 0-12 h ($AUC_{0\text{-}12h}$), 0-24 h ($AUC_{0\text{-}24h}$) and 1-2 h ($AUC_{1\text{-}2h}$)

Paracetamol
The following PK parameters will be determined for paracetamol:

- Maximum concentration in plasma (C_{max}), time to C_{max} (t_{max}), Area under the concentration-time curve in plasma from zero (predose) to time of last quantifiable concentration ($AUC_{(0\text{-}t)}$)

Pharmacodynamics (PD)

- Change in glucose concentration-time curve during MMTT ($\Delta AUC\text{-}GL_{0\text{-}4h}$), change in glucose C_{max} ($\Delta GL\text{-}C_{max}$)
- Change in C-peptide concentration-time curve during GGI ($\Delta AUC\text{-}C_{ptd}$ 1-2h)

Statistical methods
According to the primary objective two independent tests will be done in this study, i.e. MMTT and GGI. The primary variables will be analysed using analysis of covariance (ANCOVA) models with treatment and period as factors and baseline of the primary variable as a covariate. The primary variables will be log-transformed prior to the analysis and the results then transformed back to the linear scale, and significant tests will be 1-sided at 10% significance level.

Treatment estimates will be presented, and estimated mean ratios with 90% 1-sided confidence intervals and p-values will be presented for the treatment contrast of the one dose of AZD1981. The analysis will be made using the PD analysis set.

The analyses of safety tolerability, pharmacokinetic, secondary pharmacodynamic data will be summarised descriptively including tables, listings and graphs, as appropriate.

The relationship between AZD1981 exposure and effect on the primary pharmacodynamic variables will be primarily explored graphically.

TABLE OF CONTENTS PAGE

PROTOCOL SYNOPSIS 2
TABLE OF CONTENTS 6
LIST OF ABBREVIATIONS AND DEFINITION OF TERMS 10
1. INTRODUCTION 12
1.1 Background 12
1.2 Rationale for conducting this study 12
1.3 Benefit/risk and ethical assessment 13
2. STUDY OBJECTIVES 14
2.1 Primary objective 14
2.2 Secondary objectives 14
2.3 Exploratory objectives 14
3. STUDY PLAN AND PROCEDURES 15
3.1 Overall study design and flow chart 15
3.1.1 Study procedures 15
3.1.2 Mixed meal tolerance test (MMTT) on Day -1 and Days 3, 6 and 9 21
3.1.3 Graded Glucose Infusion with GLP1 (GGI) on Day -2 and Days 2, 5 and 8 21
3.1.4 Other PD and PK assessments 21
3.2 Rationale for study design, doses and control groups 22
4. SUBJECT SELECTION CRITERIA 23
4.1 Inclusion criteria 23
4.2 Exclusion criteria 24
5. STUDY CONDUCT 25
5.1 Restrictions during the study 25
5.2 Subject enrolment and randomisation 26
5.2.1 Replacement procedure 26
5.2.2 Procedures for randomisation 27
5.3 Procedures for handling incorrectly randomised subjects 27
5.4 Blinding and procedures for unblinding the study 27
5.4.1 Methods for ensuring blinding 27
5.4.2 Methods for unblinding the study 27
5.5 Treatments 28
5.5.1 Identity of investigational product(s) 28
5.5.2 Doses and treatment regimens 28

5.5.3 Labelling 29
5.5.4 Storage 29

5.6 Concomitant and post-study treatment(s) 29

5.7 Treatment compliance 29
5.7.1 Accountability 29

5.8 Discontinuation of investigational product and withdrawal from the study 30
5.8.1 Procedures for withdrawal of a subject from the study 30

6. COLLECTION OF STUDY VARIABLES 30

6.1 Recording of data 31
6.1.1 Electronic data capture 31

6.2 Enrolment and screening procedures 31
6.2.1 Follow-up procedures 32

6.3 Safety 32
6.3.1 Definition of adverse events (AEs) 32
6.3.2 Definitions of serious adverse event (SAE) 33
6.3.3 Recording of AEs 33
6.3.4 Reporting of SAEs 35
6.3.5 Laboratory safety assessment 35
6.3.6 Physical examination 37
6.3.7 ECG 37
6.3.7.1 Resting 12-lead ECG 37
6.3.8 Vital signs 37
6.3.8.1 Pulse and blood pressure 37
6.3.8.2 Body temperature 37
6.3.9 Other safety assessments 38

6.4 Pharmacokinetics 38
6.4.1 Collection of PK samples 38
6.4.2 Determination of drug concentration in PK samples 38

6.5 Pharmacodynamics 38
6.5.1 Collection of PD samples 38

6.6 Pharmacogenetics 39

7. BIOLOGICAL SAMPLING PROCEDURES 39

7.1 Volume of blood 39

7.2 Handling, storage and destruction of biological samples 40
7.2.1 PK samples 40
7.2.2 PD samples 40
7.2.3 Pharmacogenetic samples 40
7.2.4 Exploratory biomarkers 40

7.3 Labelling and shipment of biohazard samples 40

7.4 Chain of custody of biological samples 41

7.5 Withdrawal of informed consent for donated biological samples 41

8. ETHICAL AND REGULATORY REQUIREMENTS 42

8.1 Ethical conduct of the study 42

8.2 Subject data protection 42

8.3 Ethics and regulatory review 42

8.4 Informed consent 43

8.5 Changes to the protocol and ICF 43

8.6 Audits and inspections 44

9. STUDY MANAGEMENT 44

9.1 Pre-study activities 44

9.2 Training of study site personnel 44

9.3 Monitoring of the study 44
9.3.1 Source data 45

9.4 Study agreements 45
9.4.1 Archiving of study documents 45

9.5 Study timetable and end of study 45

10. DATA MANAGEMENT 46

11. EVALUATION AND CALCULATION OF VARIABLES 46

11.1 Calculation or derivation of safety variable(s) 46
11.1.1 Other significant AEs 46

11.2 Calculation or derivation of PK variables 47
11.2.1 PK of AZD1981 47
11.2.2 PK of Paracetamol 47

11.3 Calculation or derivation of PD variables 48

11.4 Calculation or derivation of the relationship between PK and PD variables 49

11.5 Calculation or derivation of pharmacogenetic variables 50

12. STATISTICAL METHODS AND SAMPLE SIZE DETERMINATION 50

12.1 Description of analysis sets 50
12.1.1 General principles 50
12.1.2 Analysis of Safety population 50
12.1.3 PK analysis set 50
12.1.4 PD analysis set 51

12.2 Methods of statistical analyses 51
12.2.1 General principles 51

12.2.2 Subject characteristics 51
12.2.3 Safety and tolerability 51
12.2.4 Pharmacokinetics 52
12.2.4.1 AZD1981 52
12.2.4.2 Paracetamol 52
12.2.5 Pharmacodynamics 52
12.2.5.1 Primary variables 52
12.2.5.2 Descriptive presentations related to the primary objective 52
12.3 Determination of sample size 52
13. IMPORTANT MEDICAL PROCEDURES TO BE FOLLOWED BY THE INVESTIGATOR 53
13.1 Medical emergencies and AstraZeneca/CRO contacts 53
13.2 Overdose 54
13.3 Pregnancy 54
13.3.1 Maternal exposure 54
13.3.2 Paternal exposure 54
14. LIST OF REFERENCES 55

LIST OF TABLES

Table 1 Study Plan - General overview 17
Table 2 Study Plan - Time schedule for Mixed Meal Tolerance Test (MMTT) on Days -1, 3, 6 and 9 19
Table 3 Graded glucose and GLP1 infusion (GGI with GLP1) schedule on Days -2, 2, 5 and 8 20
Table 4 Identity of investigational product(s) 28
Table 5 Volume of blood to be drawn from each subject 39

LIST OF FIGURES

Figure 1 Study Flow Chart 15

LIST OF APPENDICES

Appendix A Signatures
Appendix B Additional Safety Information
Appendix C IATA 6.2 Guidance document
Appendix D Pharmacogenetic Research
Appendix E Actions required in cases of combined increase of Aminotransferase and Total Bilirubin - Hy’s Law

LIST OF ABBREVIATIONS AND DEFINITION OF TERMS

The following abbreviations and special terms are used in this study protocol.
The abbreviations for the pharmacokinetic (PK) and pharmacodynamic (PD) variables and calculations are explained in the main body of the study protocol under their respective sections.

Abbreviation or special term	Explanation
AE	Adverse event (see definition in Section 6.3.1)
ALP	Alkaline phosphatase
ALT	Alanine aminotransferase
ANCOVA	Analysis of covariance
AST	Aspartate aminotransferase
AUC	Area under plasma concentration time curve
BMI	Body Mass Index
BP	Blood pressure
C	Plasma concentration
cAMP	Cyclic adenosine monophosphate
CD11b	Cluster of Differentiation molecule 11B
CHO	Carbohydrate
CIU	Chronic Idiopathic Urticaria
COPD	Chronic obstructive pulmonary disease
CRF	Case Report Form (electronic/paper)
CRO	Clinical Research Organisation
CRP	C-reactive protein
CRTh2	Chemoattractant receptor-homologous molecule expressed on Th2 cells
CVMD	Cardiovascular and Metabolic Disease
CYP3A4	Cytochrome P450 3A4
DCCT	Diabetes Control and Complications Trial
DDI	Drug-drug Interaction
DMR EPIC	Dynamic mass redistribution of cellular contents using the Corning Epic system
EC50	Half maximal effective concentration of a drug (measure of a drug's potency)
ECG	Electrocardiogram
pECG	Paper print-out ECG
EndoC-βH1	A genetically engineered human pancreatic beta cell line exhibiting glucose-inducible insulin secretion
FSH	Follicle stimulating hormone
GGI	Graded Glucose infusion
GLP1	Glucagon-like peptide-1
GPR44	G protein-coupled receptor 44
GRand	Global Randomisation System
GSIS	Glucose Sensitive Insulin Secretion
h	hour

Abbreviation or special term	Explanation
HbA1C	Glycated haemoglobin (A1c), which identifies average plasma glucose concentration
HBE	Harris Benedict Equation
HIV	Human Immunodeficiency Virus
IB	Investigator’s Brochure
ICF	Informed Consent Form
ICH	International Committee of Harmonisation
IP	Investigational Product
IRB	Institutional Review Board
LFT	Liver Function Test
LH	Luteinizing hormone
MedDRA	Medical Dictionary for Regulatory Activities
MMTT	Mixed Meal Tolerance Test
MoA	Mode of Action
OAD	Oral Anti-Diabetic
OAE	Other Significant Adverse Event (see definition in Section 11.1.1)
PD	Pharmacodynamics
PGD2	Prostaglandin D2
PK	Pharmacokinetics
PoM	Proof-of-Mechanism
QC	Quality control
R&D	Research and Development
S	Serum
SAE	Serious adverse event (see definition in Section 6.3.2).
SHGBG	Sex Hormone Binding Globulin
ss	Steady state
SUSAR	Suspected Unexpected Serious Adverse Reactions
T2DM	Type 2 Diabetes Mellitus
Th2	Type 2 T helper cells
U	Urine
ULN	Upper Limit of Normal
WBDC	Web Based Data Capture

1. INTRODUCTION

1.1 Background

CRTh2 (chemoattractant receptor-homologous molecule expressed on Th2 cells) or G protein-coupled receptor 44 (GPR44) receptor antagonists are in pharmaceutical clinical testing predominantly for the treatment of asthma and other allergic and inflammatory conditions (Norman 2014). AZD1981 is a potent, selective, reversible antagonist at GPR44, which has been evaluated by AstraZeneca in 17 clinical studies (Schmidt et al 2013). The development focus to date has been in asthma and COPD while the current development focus for AZD1981 is the early clinical exploration for type 2 diabetes mellitus (T2DM).
The predominant endogenous agonist for GPR44 is prostaglandin D2 (PGD2). PGD2 is significantly elevated in patients with asthma, and the major source is believed to be the mast cell. Thus, activation of the GPR44 receptor by PGD2 is thought to be mediated via allergic stimulation of the mast cells and hence the pharmaceutical rationale for therapeutic evaluation of GPR44 receptor antagonists in allergic conditions. However, there is also notably a high expression of GPR44 receptors on human pancreatic beta cells (Lindskog et al 2012). Functional *in vitro* studies on human beta cells and pancreatic islets have demonstrated that during a PGD2 tone GPR44 antagonists can increase glucose sensitive insulin secretion (GSIS), improve the Glucagon-like peptide-1 (GLP1) response and also have anti-apoptotic effects when cytotoxic cytokines are present. However, due to the very low number of GPR44 receptors on rodent beta cells, neither acute nor chronic *in vivo* pharmacodynamic (PD) data has been possible to generate. In summary, the demonstrated effects on human islets, with improved insulin secretion as well as cell protective effects, warrant further studies of GPR44 antagonists as a potential novel therapeutic modality in T2DM.

1.2 Rationale for conducting this study

The primary purpose of this study is to increase the human target validation of GPR44 antagonism as a novel anti-diabetic treatment modality as well as to explore the glycaemic mechanism of action (MoA) of GPR44 antagonism. AZD1981 is a CRTh2 receptor/GPR44 antagonist and a potent inhibitor of CRTh2-mediated adhesion molecule (CD11b) expression and shape change induced by PGD_2 or analogues in human eosinophils *in vitro*. AZD1981 also inhibits PGD_2 induced chaemotaxis in human eosinophils and Th2 cells. AZD1981 also potently restores GSIS by elevation of intracellular cAMP levels induced by PGD_2 or analogues in human beta cells (EndoC-βH1) and in primary human islets in *vitro*. Given the availability of adequate clinical GPR44 antagonists the aim of this study is to explore acute effects of GPR44 antagonism on key Proof-of-Mechanism (PoM) biomarkers e.g. GSIS. This will then provide confidence that pharmacological modulation of the target is relevant in a therapeutic disease context e.g. T2DM. Further on, as GLP1 responsiveness is intrinsically linked to GPR44 MoA that PD variable will be assessed as a primary variable. Other contributing MoA aspects e.g. glucagon secretion and beta cell responsiveness will also be assessed as secondary variables.

In addition, as an exploratory objective, different patient segmentation and treatment response biomarkers will be explored as well as exposure-response correlation of the PD variables.

1.3 Benefit/risk and ethical assessment

GPR44 antagonism is due to its novel, multimodal MoA on pancreatic beta cells expected to deliver superior anti-hyperglycaemic efficacy through increased glucose-dependent insulin secretion and increased incretin action as well as providing beta-cell protection via its anti-apoptotic effects. Therefore, GPR44 antagonism has the potential to provide superior disease control to existing oral agents.

Oral small molecule GPR44 antagonists are in clinical development for the treatment of asthma, chronic obstructive pulmonary disease (COPD), and other inflammatory conditions. AZD1981 has previously been in Phase 2B testing for asthma and COPD while the current development focus for AZD1981 is the early clinical exploration for T2DM. Further on, AZD1981 has adequate pharmacokinetic (PK), PD and safety properties to be amenable for this PoM study. In the current study, a dosing regimen of 200 mg AZD1981 daily for three days is proposed. Both the proposed dose and the treatment duration in this study are not expected to pose either any safety or tolerability issues. This is based on previous studies where AZD1981 doses of up to 1000 mg b.i.d. for 4 weeks or 400 mg for 12 weeks were administered in a total of 17 clinical studies with an acceptable tolerability profile. The emerging safety profile of AZD1981 states only an association with increase in hepatic enzymes. However, the hepatic enzyme findings have only been seen in exposures exceeding 800 mg daily. Hence, in the current study where only a dose of 200 mg for three days will be used, the risk for increase in hepatic enzymes has been judged to be minor. Nevertheless, hepatic safety laboratory parameters will be assessed and closely watched. Further on, experiments with human hepatocytes have shown that AZD1981 has the potential to both inhibit and induce metabolic enzyme activity. Specific clinical drug-drug interaction studies have been completed. For details please refer to the Investigator's Brochure (IB).
A daily dose of 200 mg is not believed to pose any significant risk for altered exposure of concomitant medications as AZD1981 caused weak inhibition of CYP2C9 activity at 100 mg b.i.d. (increase in S warfarin AUC by 35%), inhibition of organic anion-transporting polypeptide 1B1 (OATP1B1) activity at 100 mg b.i.d. (increase in pravastatin AUC by 27 %) and negligible induction of cytochrome P450 3A4 (CYP3A4) activity (where 500 mg b.i.d. decreased midazolam area under the curve (AUC) by 27%). Further on, AZD1981 is also being explored in other allergic inflammatory conditions e.g. antihistamine non-responding chronic idiopathic urticaria (CIU) in an ongoing Investigator Sponsored Study, which is a randomised, double-blind, placebo-controlled 8-week study in a total of 48 patients at John Hopkins University, USA. A daily dose of 3x40 mg is compared to placebo in the study. The study commenced in Q1 2014 and is expected to be completed in Q1 2015.

The clinical findings to date give confidence that the known risks can be mitigated, and that the proposed 3-day dosing regimen, can be conducted without undue risk to patient safety (provided that patients' safety is monitored in accordance with this clinical study protocol) and meets the standard of ethics and patient safety.

2. STUDY OBJECTIVES

2.1 Primary objective

- To assess the change in plasma glucose after mixed-meal tolerance test (MMTT) compared to baseline
  - Change in glucose concentration-time curve ($AUC_{0\text{-}4\,h}$)
  - Change in glucose C_{max} level
- To assess the change in insulin secretion rate by assessment of C-peptide after graded glucose and GLP1 infusion (GGI)

2.2 Secondary objectives

- To assess the change in plasma insulin, c-peptide and glucagon after MMTT compared to baseline
- To assess the change in glucose and insulin after GGI
- To assess the change in 24 h glucose, fasting insulin and beta cell responsiveness after MMTT and GGI
- To assess the pharmacokinetics (PK) of AZD1981 after repeated oral doses
- To assess the change in paracetamol in plasma during MMTT
- To assess safety and tolerability after repeated oral doses of AZD1981 in T2DM patients

2.3 Exploratory objectives

- To assess changes in the primary endpoints based on exploratory patient segmentation biomarkers (plasma and urine PGD2-metabolites, P-SHBG, B-Eosinophils)
- To explore the relationship between AZD1981 exposure and PD variables e.g. MMTT AUC_{PD} and GGI AUC_{PD}
- To collect and store plasma samples for potential future exploratory research aimed at exploring biomarkers involved associated with the pharmacological action of GPR44 antagonism and the PGD2-GPR44 pathway
- To collect and store blood samples for potential future exploratory genetic research aimed at identifying/exploring genetic variations that may affect pharmacokinetics and pharmacodynamics, safety and tolerability related to GPR44 antagonism and the PGD2-GPR44 pathway (see Appendix D of this Clinical Study Protocol)

3. STUDY PLAN AND PROCEDURES

This Clinical Study Protocol has been subject to a peer review according to AstraZeneca standard procedures.

3.1 Overall study design and flow chart

This is a Phase I, randomised, double-blind, placebo-controlled, multiple-dose, cross-over study conducted at a single centre in n=20 fully evaluable (i.e. completed both treatment periods with full primary PD assessments done) male and female T2DM patients. In order to ascertain n=20 evaluable patients, up to n= 25 patients may be randomised and dosed.

The study design will consist of run-in period that will include screening and enrolment procedures, and 2 treatment periods of 3 days each, separated by a washout period of 3 days. During the treatment periods the study subjects, staying at the research unit, will receive orally 100 mg (10 tablets of 10 mg) of AZD1981 or placebo, 2 times per day, for 3 consecutive days. The total daily dose of active AZD1981 per subject and day will be 200 mg.

The overall study design is shown in Figure 1.

Figure 1 Study Flow Chart

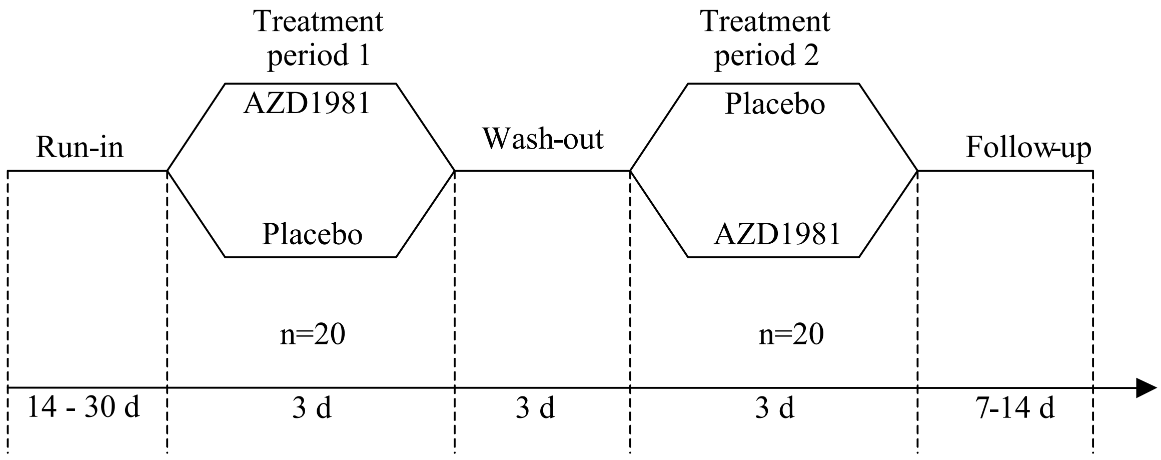

3.1.1 Study procedures

Screening procedure and start of run-in
Metformin-treated T2DM patients with inadequate glycaemic control (HbA1c) will be screened and recruited at the Screening visit (Visit 1). After study inclusion they will go through a run-in period of at least 14-30 days.

Eligibility assessments and Randomisation
After completion of the run-in period (Visit 2, Day-3 after overnight fast) the subjects will undergo physical examination including vital signs, measurement of body weight, ECG and safety laboratory assessments. Eligible subjects will then be randomised and admitted to the research unit for a 13-day residential study period (Day -3 to Day 10).

Eligible subjects, will receive a standardised, individualised, weight-maintaining diet, i.e. the same menu across the study with a calorie intake individualised to 100-200 kcals of HBE x 1.5 (Harris Benedict Equation) to be applied starting on Day-1 and maintained throughout the residential stay at the unit. Subjects will be encouraged to consume 100% of meals during their stay.

Baseline pharmacodynamic (PD) assessments

On Day -2, after an overnight fast, the 1st baseline PD (graded glucose and GLP1 infusion (GGI/GLP1)) and exploratory assessments (biomarker blood sampling) for the 1st treatment period will be conducted.

On Day -1, after an overnight fast, the second baseline PD (MMTT) and exploratory assessments for the 1st treatment period will be conducted.

Treatment periods and wash-out

On Day 1 (1st day of the 1st Treatment period) the subjects will receive the 1st dose of AZD1981 and the following assessments will be done: recording of concomitant medication, serious Adverse Event (SAE) reporting and start of non-serious Adverse Event reporting.

On Day 2, Day 5 (1st baseline for the 2nd treatment period) and on Day 8, the PK, PD (e.g. GGI/GLP1) and exploratory assessments will be repeated. On Day 3 (last day of the 1st treatment period), Day 6 (2nd baseline for the 2nd treatment period) and on Day 9 (last day of the 2nd treatment period) the PK, PD (e.g. MMTT) and exploratory assessments, will be repeated. Paracetamol challenge, for assessing delayed gastric emptying during the MMTT, will be performed on Days 3 and 9.

Days 4-6 are wash-out days.

The study treatments will be assigned in a random order. The subjects who received active treatment during the 1st Treatment period will receive placebo during the 2nd Treatment period and vice versa. The final PD, PK and exploratory assessments will be conducted on Day 9 (the last day of the 2nd treatment period) and the subjects will be discharged on the morning of Day 10. They will be asked to return for a follow up visit 7-14 days later.

Follow-up assessments

The follow-up visit (Visit 3), after an overnight fast, will include the following assessments: physical examination (incl. measurement of body weight and vital signs), ECG, safety laboratory measurements and AE reporting.

Table 1 Study Plan - General overview

**Study period**	**Run-in (14-30 days*)**				**1st treatment period**			**Wash-out**			**2nd treatment period**				**Follow-up**
**Visits**	**1**	**2**													**3**
	**Enrolment** (at the start of run-in)	**Day -3**	**Day -2**	**Day -1**	**Day 1**	**Day 2**	**Day 3**	**Day 4**	**Day 5**	**Day 6**	**Day 7**	**Day 8**	**Day 9**	**Day 10**	**Post-study** (7-14 d)
Informed consent	X														
Inclusion/exclusion criteria	X	X													
Demographic data	X														
Other subject characteristics [a]	X	X													
Medical & Surgical history	X	X													
**Randomisation**		X													
Study residency		X	X	X	X	X	X	X	X	X	X	X	X	X	
Non-residential visit	X														X
**Study drug administration**					X	X	X				X	X	X		
**Safety and tolerability:**															
Adverse event (AE) recording					X	X	X	X	X	X	X	X	X	X	X
Serious AE (SAE) recording	X	X	X	X	X	X	X	X	X	X	X	X	X	X	X
Vital signs [b]	X	X					X						X		X
12-lead ECG	X	X					X						X		X
Laboratory safety assessment [c]	X	X					X						X		X
Body weight	X	X					X						X		X
Height and BMI	X														
Diet review and planning of weight-maintaining diet		X													
Standardised weight-maintaining diet			X	X	X	X	X	X	X	X	X	X	X	X	
Pregnancy test	X	X													X
Drug and alcohol screen	X	X													
Concomitant medication	X	X	X	X	X	X	X	X	X	X	X	X	X	X	X
Full physical examination [d]	X														X
Brief physical examination [d]		X												X	

Table 1 Study Plan - General overview

**Study period**	**Run-in (14-30 days*)**				**1st treatment period**			**Wash-out**			**2nd treatment period**				**Follow-up**
**Visits**	**1**	**2**													**3**
	**Enrolment** (at the start of run-in)	**Day -3**	**Day -2**	**Day -1**	**Day 1**	**Day 2**	**Day 3**	**Day 4**	**Day 5**	**Day 6**	**Day 7**	**Day 8**	**Day 9**	**Day 10**	**Post-study** (7-14 d)
**Pharmacodynamics (PD)** [e]**:**															
GGI with GLP1 (Graded glucose and GLP1 infusion)			X			X			X			X			
MMTT (Mixed meal tolerance test)				X			X			X			X		
Blood: insulin, glucose, C-peptide			X	X		X	X		X	X		X	X		
Blood: total GLP1			X			X			X			X			
Blood: glucagon and total GLP1 [f]				X			X			X			X		
Blood: 7p Glucose				X			X			X			X		
Exploratory Biomarkers:															
Blood: P-PGD2 metabolites, P-SHBG, B-Eosinophils [g]				X			X			X			X		
Urine: PGD2 metabolites [g]				X			X			X			X		
Blood: Future biomarkers [g]				X			X			X			X		
**Pharmacokinetics (PK):**															
Blood: AZD1981						X	X					X	X		
Blood: Paracetamol							X						X		
**Pharmacogenetics**		X													

* Timings relative to dose administration on Day 1
a For details see Section 6.2
b Supine blood pressure, pulse rate and body temperature, see Section 6.3.8
c Laboratory safety assessment according to Section 6.3.5
d For details see Section 6.3.6
e For details see Table 2 and Table 3 and Section 6.5
f Analysis of GLP1 will be done on the blood collected for the glucagon sample
g For details see Table 2

Table 2 Study Plan - Time schedule for Mixed Meal Tolerance Test (MMTT) on Days -1, 3, 6 and 9

Protocol time (hh:mm)	Ensure Plus® drink intake (over 5-10 min)	Blood sample Exploratory biomarkers	Blood sample Paracetamol PK[a]	Blood sample AZD1981 PK[a]	Blood sample 7p Glucose	Blood sample glucagon and total GLP1	Blood sample glucose, C-peptide and insulin	Other activities
-01:15		X[b]						- Administration of oral medications<br>- Safety lab [a]<br>- PD urine collection [c]<br>- 12-lead ECG [a], Vital signs [a] and body weight [a]
-00:15						X	X	
-00:01			X	X	X	X	X	
00:00	X							- Study drug intake (before the start of Ensure Plus® drink) [a],<br>- Paracetamol solution intake (immediately after the intake of Ensure Plus® drink) [a]
00:15			X			X	X	
00:30			X			X	X	
01:00			X	X		X	X	
01:30			X				X	
02:00			X	X	X	X	X	
02:30								
03:00			X			X	X	
03:30								
04:00			X	X	X	X	X	
06:00					X			
08:00								
09:00					X			
10:00								
12:00				X	X			Study drug intake [a]
14:00					X			
18:00					X			
24:00				X	X			

a Only applicable on Days 3 and 9
b Exploratory biomarkers: P-PGD2 metabolites, P-SHBG & B-Eosinophil count and plasma samples for future project-related use
c Pre-dose urine for PGD2, 2nd void morning collection

Table 3 Graded glucose and GLP1 infusion (GGI with GLP1) schedule on Days -2, 2, 5 and 8

Protocol time (hh:mm)	Administration of oral medications	Blood sample AZD1981 PK [a]	GGI [b]	GLP1 infusion [b]	Blood sample glucose, C-peptide and insulin	Blood sample total GLP1	Other activities
-01:15	X						
-00:15					X		
-00:01		X			X	X	
00:00			X				Study drug intake (before the start of GGI) [a]
00:15					X		
00:30			X		X		
00:45					X		
01:00		X	X	X	X	X	
01:10				X		X	
01:15					X	X	
01:30			X		X		
01:45					X		
02:00		X			X	X	

a Only applicable on Days 2 and 8
b For details on GGI and GLP1 infusion rates and dosing volumes see Section 3.1.3

3.1.2 Mixed meal tolerance test (MMTT) on Day -1 and Days 3, 6 and 9

All oral medications including OAD, e.g. metformin, will be given and taken 75 min prior to the start of MMTT. Patients will be fasting from 10 pm the previous day. They are allowed to drink water but no other fluids up to one hour before dosing.

Two cannulae will be inserted (one in each forearm) following the (optional) application of topical anaesthetic cream. These will be kept patent by slowly running saline drip.

Two baseline blood samples, for analyses of blood glucose, C-peptide and insulin, will be taken prior to the Ensure Plus® drink. At the time 0 min, on Days 3 and 9 after the intake of the study drug, the patient will be asked to drink Ensure Plus® drink (472 mL, 16 Oz, 100 g CHO) over a period of 5-10 min. Immediately after completion of the drink, the patient will be given 1000 mg paracetamol solution.

Blood samples after the intake of Ensure Plus® drink will be taken as specified in Table 2.

The total blood volume drawn from each subject will be specified in Section 7.1.

3.1.3 Graded Glucose Infusion with GLP1 (GGI) on Day -2 and Days 2, 5 and 8

All oral medications including OAD, e.g. metformin, will be given and taken 75 min prior to the start of the GGI. Patients will be fasting from 10 pm the previous day. They are allowed to drink water but no other fluids up to one hour before dosing.

Two cannulae will be inserted (one in each forearm) following the (optional) application of topical anaesthetic cream. These will be kept patent by slowly running saline drip.

At time t=00:00 (hh:mm), on Days 2 and 8 after the intake of the study drug, a graded glucose infusion will be started using 20% glucose following the schedule: 2 mg/kg/min for 30 min, 4 mg/kg/min for 30 min, 6 mg/kg/min for 30 min and 8 mg/kg/min for 30 min.

Blood samples will be taken at 15-minute intervals between 0 and 60 min.

At time t=01:00 (hh:mm), GLP1 infusion will be started, with an infusion rate of 2 pmol/min/kg for 10 min, then reducing to 1 pmol/min/kg for 50 min.

Blood samples will be taken at 15-min intervals between 60 and 120 min.

For sampling time schedule see Table 3.

The total blood volume drawn from each subject will be specified in Section 7.1.

3.1.4 Other PD and PK assessments

On the efficacy/pharmacodynamic assessment days also a 7-point (7p) glucose curve ($AUC_{0\text{-}24h}$) will be determined on Days –1, 3, 6 and 9. For details see Table 2 in Section 3.1.1.

Samples for the determination of AZD1981 levels in plasma will be taken on Days 2, 3, 8 and 9. For details see Table 2 and Table 3 in Section 3.1.1.

Samples for the determination of paracetamol levels in plasma will be taken on Days 3 and 9. For details see Table 2 in Section 3.1.1.

3.2 Rationale for study design, doses and control groups

Study design, choice of control group(s) and treatment duration

A randomised, double-blind, placebo-controlled study design is standard in clinical drug studies and is considered the best choice to achieve the objectives of the study, from both safety and efficacy perspectives. Further on, inclusion of a cross-over design is an established means of reducing sample size and improving statistical power. The study is designed to demonstrate superior efficacy versus placebo.

Patient population

Metformin will be the drug of choice for the vast majority of newly diagnosed T2DM patients for the foreseeable future according to T2DM treatment guidelines. However, many patients with T2DM do not reach glycaemic control goals with metformin monotherapy and the addition of a glucose-lowering agent, with a different mechanism of action, is indicated. This motivates the inclusion of metformin-treated T2DM. Further on, preclinical efficacy data highlight the dependence on high glucose for GPR44 antagonism to exert its beneficial effects on insulin secretion. Hence, the rationale for testing of the concept in hyperglycaemic subjects with inadequate control on metformin.

Choice of dosing

A dose of 200 mg is proposed for the PoM study as:

- Steady state is achieved after 1 day
- C_{min} 3-fold greater than the free DMR EPIC potency binding data (EC50 of 1.3nM)
- Adequate safety margin versus the observed LFT effects at >800 mg/day
- Adequate safety margin versus significant DDI at doses >800 mg/day

Pharmacodynamic variables

The two key glycaemic efficacy parameters that have been demonstrated on human beta cells and human islets are: 1) an increase in GSIS (key Proof-of-Mechanism biomarker) and 2) increase in GLP1-mediated insulin secretion e.g. increased GLP1 responsiveness. Hence both the primary and the secondary variables have been chosen, appropriately based on a well-established pharmacodynamic assessment. To assess GSIS in a physiological way, incorporating incretin and neuronal components, a MMTT method has been chosen. Further on, due to the co-localisation of GLP1R and GPR44 in submucosal neuronal tissue of human gastric and intestine and thus the possibility of delayed gastric emptying, the MMTT has been extended to 4 hours and also a paracetamol test been included. Moreover, to assess GLP1

responsiveness, a GGI pharmacodynamic method with concomitant GLP1 infusion has been chosen in order to assess the GLP1 response over a wide range of glucose concentrations.

4. SUBJECT SELECTION CRITERIA

Investigators should keep a record i.e., subject screening log, of subjects who entered study screening.

Each subject must meet all of the inclusion criteria and none of the exclusion criteria at the time of randomisation for this study. Under no circumstances can there be exceptions to this rule.

4.1 Inclusion criteria

For inclusion the study subjects should fulfill the following criteria at the time of enrolment:

1. Provision of informed consent prior to any study specific procedures
2. Male or female of non-childbearing potential (postmenopausal, and/or have undergone hysterectomy and/or bilateral oophorectomy or salpingectomy/tubal ligation) aged ≥18. Women will be defined as postmenopausal if last menstruation period was >1 year ago and serum FSH and LH are within the postmenopausal range, or if age >50 years and with last menstruation period >2 years ago.
3. Patients with HbA1c ≥7.5 but ≤11% at enrolment visit (Visit 1) (HbA1c value according to international DCCT standard)
4. Body mass index ≥19 to ≤38 kg/m^2
5. The fasting plasma glucose should be in the range of 3-14 mmol/L (54-252 mg/dL, inclusive) on the morning of Visit 1.
6. Clinical diagnosis of type 2 diabetes mellitus
7. Metformin as only anti-diabetic treatment, at least for the last 3 months

For inclusion in the genetic component of the study subjects must fulfil the following additional criterion:

8. Provision of signed, written, and dated informed consent for optional genetic research. If a subject declines to participate in the genetic component of the study, there will be no penalty or loss of benefit to the volunteer. The subject will not be excluded from other aspects of the study described in this CSP, as long as they consent

4.2 Exclusion criteria

Subjects should not enter the study if any of the following exclusion criteria are fulfilled at Visit 1 or at the start of Visit 2:

1. History or sign of any clinically significant disease or disorder which, in the opinion of the investigator, may either put the subject at risk because of participation in the study, or influence the results or the subject's ability to participate in the study
2. Any clinically significant abnormalities in clinical chemistry, haematology or urinalysis results as judged by the investigator
3. Aspartate aminotransferase (AST) or alanine aminotransferase (ALT) laboratory results >3x upper level of normal range (ULN)
4. Any positive result on screening for serum hepatitis B surface antigen, hepatitis C antibody and human immunodeficiency virus (HIV)
5. Pregnant or planning to be pregnant during the study
6. Clinical diagnosis of Type 1 diabetes mellitus and/or history of diabetic ketoacidosis or positive Glutamic Acid Decarboxylase Autoantibodies test (GAD antibodies test).
7. Patients treated with single Insulin therapy within the last 3 months
8. Known or suspected history of significant drug abuse within the past 5 years in the opinion of the investigator
9. History of alcohol abuse or excessive intake of alcohol as judged by investigator.
10. Regular smoking of more than 5 cigarettes per week or consumption of more than 3 portion of snuff or equivalent per day within 30 days before administration of study drug
11. History of severe allergy/hypersensitivity or ongoing allergy/hypersensitivity, as judged by the investigator
12. Plasma donation within one month of screening or any blood donation during the 3 months prior to screening
13. Non-leukocyte depleted whole blood transfusion within 120 days of the date of the genetic sample collection (see Appendix D of this Clinical Study Protocol for details of pharmacogenetic research).
14. Any other condition with in the opinion of the investigator would render the patient unsuitable for inclusion in the study and /or for the patients safety
15. Involvement in the planning and/or conduct of the study (applies to both AstraZeneca staff and/or staff at the study site)
16. Judgment by the investigator that the subject should not participate in the study if considers subject unlikely to comply with study procedures, restrictions and requirements

For procedures for handling incorrectly randomised subjects see Section 5.3.

5. STUDY CONDUCT

5.1 Restrictions during the study

The following restrictions apply for the specified times during the study period:

1. Fast from at least 10 hours before the randomisation visit (Day-3), planned start of PD assessments (Days -2 to-1 and Days 5 to 6) and during the treatment days i.e. Days 1 to 3 and Days 7 to 9. A moderate amount of water is allowed up to 1 hour prior to start and may be resumed 1 hour after dosing

   A meal can be given 5 hours after the start of the assessments

   For other doses during the study days, the subject should be fasting for 1 hour before and 1 hour after the dose
2. Eat and drink only meals and drinks provided (apart from water) during the residential period in the unit. In this study, individualised weight-maintaining diet, considering body weight, body composition, gender etc., planned by a nutritionist upon admission on Day -3, will apply
3. Abstain from consuming any of the following:
   - Alcohol from 48 hours before admission, during the residential period and for 48 hours before the study follow-up visit
   - Energy drinks containing taurine or glucuronolactone e.g., Red Bull from 72 hours before admission, during the residential period and for 72 hours before the study follow-up visit
   - Caffeine-containing drinks during the residential period apart from any provided as part of a standardised meal. Excessive intake of caffeine should be avoided between discharge from the unit and the study follow-up visit
   - Poppy seeds found in speciality bread from time of consent until after the final medical examination at the study follow-up
   - Grapefruit, grapefruit juice, Seville oranges, Seville orange marmalade or other products containing grapefruit or Seville oranges from 7 days before admission until after the final medical examination at the study follow-up
4. Abstain from nicotine use, smoking and drugs of abuse from time of consent until after the final medical examination at the study follow-up
5. Abstain from taking any novel medication (prescribed or over the counter products) or making any dose adjustments of existing therapy, from 2 weeks prior to the first administration of investigational product until after the final medical examination at the study follow-up. Abstain from taking paracetamol/acetaminophen throughout the residential study period. However, this should not obviate necessary medical

treatment. If any medication is necessary during the residential period, it should be prescribed by the investigator and the AstraZeneca Study Team Physician should be informed (Section 5.6)

6. Subjects should refrain from strenuous physical activity, which is not within the subject's normal daily routine, from 48 hours prior to admission to the unit until after the final medical examination at the study follow-up
7. Abstain from blood or plasma donation until 3 months after the final medical examination at the study follow-up
8. Male subjects should use a condom to prevent pregnancy and drug exposure of a partner and refrain from donating sperm or fathering a child from the first administration of investigational product until 3 months after the last administration of investigational product

5.2 Subject enrolment and randomisation

The Principal Investigator will ensure that:

1. Signed informed consent is obtained from each potential subject before any study specific procedures are performed
2. Each potential subject is assigned a unique enrolment number in the format E000100Y (where 1 will be the centre number and Y the consecutive enrolment number)
3. The eligibility of each subject is determined See Sections 4.1 and 4.2
4. Each eligible subject is assigned a unique randomisation code (subject number), beginning with "#"

Enrolment (=Screening) is conducted at Visit 1.

Randomisation will be performed on Day -3.

Randomisation codes will be assigned strictly sequentially as subjects become eligible for randomisation.

If a subject withdraws his/her participation in the study, then his/her enrolment/randomisation code cannot be reused.

5.2.1 Replacement procedure

In case of replacement a 'like for like sequence' principle will be applied. Therefore, the randomisation will be performed for twice the needed number of subjects. If there is a need of a new randomisation code for subject replacement, the site is to contact the relevant representative at AstraZeneca to receive the correct randomisation number with the correct sequence to ensure the blinding of the study.

The replacement will be applicable if a subject has not completed both treatment periods with full primary PD assessments done.

5.2.2 Procedures for randomisation

A randomisation scheme will be produced by AstraZeneca R & D using the global randomisation system (GRand). A randomisation scheme of approximately double the intended sample number will be produced in order to enable replacements. Subjects will be allocated to AZD1981 or placebo in a ratio of 1:1. The randomisation will be done using consecutive randomisation codes (subject numbers).

5.3 Procedures for handling incorrectly randomised subjects

Subjects who fail to meet the inclusion/exclusion criteria should not, under any circumstances, be enrolled or receive study medication. There can be no exceptions to this rule.

Where a subject, who does not meet the inclusion and/or exclusion criteria, is randomised in error and this is identified before dosing, the subject should be withdrawn from the study. A discussion should occur between the AstraZeneca Study Team Physician and the investigator regarding whether a replacement may be considered.

Where a subject, who does not meet the selection criteria, is randomised in error and started on treatment, or where a subject subsequently fails to meet the study criteria post initiation, the investigator should inform the AstraZeneca Study Team Physician immediately and withdraw the subject from the study (see Section 5.8.1).

The AstraZeneca Study Team Physician is to ensure all decisions are appropriately documented.

5.4 Blinding and procedures for unblinding the study

5.4.1 Methods for ensuring blinding

This study is double-blind with regard to treatment with 10 tablets of 10 mg AZD1981 or placebo given twice daily for 3 + 3 days.

The following personnel will have access to the randomisation list:

- The AstraZeneca personnel carrying out the labelling and packaging of study drug
- The personnel analysing the PK samples

The randomisation list should be kept in a secure location until the end of the study.

5.4.2 Methods for unblinding the study

Individual treatment codes, indicating the treatment randomisation for each randomised subject, will be available to the investigators or pharmacists at the study centre.

The treatment code should not be broken except in medical emergencies when the appropriate management of the subject requires knowledge of the treatment randomisation.
The investigator documents and reports the action to AstraZeneca, without revealing the treatment given to subject to the AstraZeneca staff.

AstraZeneca retains the right to break the code for SAEs that are unexpected and are suspected to be causally related to an investigational product and that potentially require expedited reporting to regulatory authorities. Treatment codes will not be broken for the planned analyses of data until all decisions on the evaluability of the data from each individual subject have been made and documented.

Before unblinding, a decision should be made about the action to be taken based on the revealed treatment allocation.

5.5 Treatments

5.5.1 Identity of investigational product(s)

Table 4 Identity of investigational product(s)

Investigational product	Dosage form and strength	Manufacturer
AZD1981	Oral tablet, 10 mg	AstraZeneca
Placebo for AZD1981	Oral tablet	AstraZeneca

The investigational product will be supplied by AstraZeneca in a subject specific labelled container for each treatment period according to the randomisation scheme.

5.5.2 Doses and treatment regimens

Each subject will receive a 100 mg oral dose of AZD1981 (10 tablets of 10 mg) or matching placebo (10 tablets) twice daily on Days 1 to 3 of Treatment period 1. The first morning dose on each treatment day will be administered with 240 mL of water after an overnight fast of at least 10 hours. The 2nd dose will be given 12 h after the 1st dose with 240 mL of water after at least 1 hour of fasting. The same dosing pattern will be applied during the Treatment period 2 (Days 7 to 9). Subjects who received AZD1981 in the first treatment period will receive placebo in the second treatment period and subjects who received placebo in the first treatment period will receive AZD1981 in the second treatment period. The last dose in the study will be given on Day 9. There will be a washout period of 3 days between the two treatment periods.

The total daily dose of AZD1981 will be 200 mg.

On the dosing days (Days 1-3 and Days 7-9) moderate amounts of water will be allowed until 1 hour before dosing. Water will be allowed from 1 hour after dosing and a standard meal will be given 5 hours after dosing. After dosing, subjects will remain on their bed or sitting for as long as necessary for the required study procedures.

5.5.3 Labelling

Labels will be prepared in accordance with Good Manufacturing Practice and local regulatory guidelines. The labels will fulfill Good Manufacturing Practice Annex 13 requirements for labelling.

5.5.4 Storage

All study drugs should be kept in a secure place under appropriate storage conditions. The investigational product label on the container specifies the appropriate storage.

5.6 Concomitant and post-study treatment(s)

No novel concomitant medication or therapy, or dose adjustments of existing therapy will be allowed. The subjects should abstain from taking paracetamol/acetaminophen throughout the residential study period. The subjects should be instructed that no other medication is allowed including herbal remedies, vitamin supplements and over-the-counter products without the consent of the investigator.

Medication, which is considered necessary for the subject’s safety and well-being, may be given at the discretion of the investigator during the residential period. When any medication is required, it should be prescribed by the investigator who should inform the AstraZeneca Study Team Physician. Following consultation with the Study Team Physician, the investigator should determine whether or not the subject should continue in the study.

5.7 Treatment compliance

The administration of all medication (including investigational product) should be recorded in the appropriate sections of the Case Report Forms.

Treatment compliance will be assured by supervised administration of the investigational product by the investigator or delegate. The dose, dates and times of administration of the investigational product will be recorded and checked by the monitor at monitoring visits.

5.7.1 Accountability

It is the investigator’s responsibility to establish a system for handling study treatments, including investigational medicinal products, to ensure that:

1. Deliveries of such products from AstraZeneca are correctly received by a responsible person (*e.g.,* pharmacist)
2. Such deliveries are recorded
3. Study treatments are handled and stored safely and properly
4. The study drug provided for this study will be used only as directed in the study protocol

5. The study personnel will account for all drugs received at the site, dispensed for the subject and returned to the pharmacy. Any discrepancies should be documented, investigated and appropriately resolved

6. At the end of the study, site personnel will account for all unused drugs and for destruction/return to a designated facility for destruction. Certificates of delivery and destruction/return should be signed. Destruction must not take place unless the responsible person at AstraZeneca has approved it

5.8 Discontinuation of investigational product and withdrawal from the study

Subjects may be discontinued from investigational product in the following situations:

- Subject decision. The subject is at any time free to withdraw his/her participation in the study, without prejudice
- Adverse events
- Severe non-compliance to the study protocol as judged by the investigator and/or AstraZeneca
- Randomisation in error (see Section 5.3)
- Termination of the study by the Investigator or AstraZeneca

Subjects who discontinue investigational product will be withdrawn from the study.

Subjects who are withdrawn from the study by the investigator due to adverse events after the start of dosing will be replaced. Subjects who withdraw for any reason before the first dose or for reasons other than adverse events after the start of dosing may be replaced.

5.8.1 Procedures for withdrawal of a subject from the study

Subjects are at any time free to withdraw from the study (study treatment and assessments), without prejudice (withdrawal of consent). Such subjects will always be asked about the reason(s) and the presence of any adverse events. If possible, subjects who withdraw from the study after the start of dosing and before completion should be seen by an investigator and undergo the assessments and procedures scheduled for the follow-up visit. Adverse events should be followed up (see Sections 6.3.3 and 6.3.4).

6. COLLECTION OF STUDY VARIABLES

The study assessments are described in the sections below and the timings of these assessments are detailed in the Study Plans (Table 1, Table 2 and Table 3).

It is important that PD sampling occurs as close as possible to scheduled time. In order to achieve this, other assessments scheduled at the same time may be initiated prior to the time point. The sequence at a particular time point is:

1. Pharmacodynamic blood samples
2. Pharmacokinetic blood samples
3. Exploratory biomarker samples
4. Blood pressure and pulse rate
5. ECG
6. Study drug administration
7. Ensure® Plus drink
8. Paracetamol solution intake

6.1 Recording of data

The investigator will ensure that data are recorded on the electronic Case Report Forms (eCRFs) as specified in the study protocol. He/she ensures the accuracy, completeness, and timeliness of the data recorded, for data queries and all required reports according to any instructions provided.

The Principal Investigator will sign the completed eCRFs. A copy of the completed eCRFs will be archived at the study site.

6.1.1 Electronic data capture

During the study days, data will be captured on paper source documents at bedside. Data will be entered from the paper source documents into the clinical study database by means of the electronic Case Report Forms.

The clinic staff will perform Quality Control (QC) checks on the source data before the data are entered into the clinical study database. Any changes made prior to investigator approval of the electronic Case Report Forms will be documented within the audit trail of the electronic data capture application. Any missing, impossible, or inconsistent entries will be queried within the electronic data capture application in accordance with Profil Institute practices.

Profil Institute will perform data entry, editing and analyses. The data entry screens used to capture the CRF data will be designed according to the AstraZeneca CRF Standard.

6.2 Enrolment and screening procedures

At enrolment (Visit 1; start of run-in period), each potential subject will provide informed consent prior to starting any study specific procedures.

Demographic data and other characteristics will be recorded and will include: date of birth, gender, race, alcohol consumption, smoking history, disease duration and diabetes heredity status.

Each subject will undergo screening assessments during the 30-14 days prior to the 1st dose to confirm eligibility (Table 1). These will consist of:

1. A standard medical, medication and surgical history with review of the inclusion and exclusion criteria with the subject
2. A complete physical examination
3. Height, weight and calculation of BMI
4. Vital signs - resting supine BP, pulse and oral body temperature
5. Recording a resting 12-lead paper ECG
6. A blood sample for routine clinical chemistry, haematology and screen for hepatitis B surface antigen, antibodies to hepatitis C virus and antibodies to HIV*
7. A urine sample for routine urinalysis, drugs of abuse screen and pregnancy test

* *If the subject shows up fasting samples will be drawn, otherwise the subject will have to come back for a new visit.*

After admission and before randomisation (Day -3) the investigator should reassess each subject to reconfirm eligibility.

6.2.1 Follow-up procedures

A post-study medical examination will be performed 7 to 14 days after the last dose. This will be similar to the one performed at the enrolment visit and will include a complete physical examination, measurement of weight, vital signs, recording a 12-lead paper ECG, a blood sample for clinical chemistry and haematology (after an overnight fast), a urine sample for urinalysis and pregnancy test, and assessment of any adverse events or required medication.

6.3 Safety

The Principal Investigator is responsible for ensuring that all staff involved in the study is familiar with the contents of this section.

6.3.1 Definition of adverse events (AEs)

An adverse event (AE) is the development of an undesirable medical condition or the deterioration of a pre-existing medical condition following or during exposure to a pharmaceutical product, whether or not considered causally related to the product.
An undesirable medical condition can be symptoms (e.g., nausea, chest pain), signs (e.g., tachycardia, enlarged liver) or the abnormal results of an investigation (e.g., laboratory findings, electrocardiogram). In clinical studies, an AE can include an undesirable medical condition occurring at any time, including run-in or washout periods, even if no study treatment has been administered.

The term AE is used generally to include any AE whether serious or non-serious.

6.3.2 Definitions of serious adverse event (SAE)

A serious adverse event (SAE) is an AE occurring during any study phase (i.e., run-in, treatment, washout, follow-up), that fulfils one or more of the following criteria:

- Results in death
- Is immediately life-threatening
- Requires in-patient hospitalisation or prolongation of existing hospitalisation
- Results in persistent or significant disability/incapacity or substantial disruption of the ability to conduct normal life functions
- Is a congenital abnormality or birth defect
- Is an important medical event that may jeopardise the subject or may require medical intervention to prevent one of the outcomes listed above

For further guidance on the definition of a SAE, see Appendix B of this Clinical Study Protocol. For definition of other significant adverse events (OAE) see Section 11.1.1.

6.3.3 Recording of AEs

Time period for collection of AEs

SAEs will be collected from the signing of Informed Consent (Visit 1) throughout the treatment period and including the follow-up period. Non-serious AEs will be collected from the start of dosing (Visit 2, Day 1) throughout the treatment period and including the follow-up period.

Follow-up of unresolved AEs

Any AEs that are unresolved at the subject's last visit in the study are followed up by the investigator for as long as medically indicated, but without further recording in the CRF. AstraZeneca retains the right to request additional information for any subject with ongoing AE(s)/SAE(s) at the end of the study, if judged necessary.

Variables

The following variables will be collected for each AE:

- AE diagnosis/description
- The date and time when the AE started and stopped
- Intensity
- Whether the AE is serious or not
- Investigator causality rating against the investigational product (yes or no)

- Action taken with regard to investigational product
- AE caused subject's withdrawal from study (yes or no)
- Outcome

Additional variables will be collected for all SAEs including treatment given for the event.

The following intensity ratings will be used:

1. mild (awareness of sign or symptom, but easily tolerated)
2. moderate (discomfort sufficient to cause interference with normal activities)
3. severe (incapacitating, with inability to perform normal activities)

It is important to distinguish between serious and severe AEs. Severity is a measure of intensity whereas seriousness is defined by the criteria in Section 6.3.2. An AE of severe intensity need not necessarily be considered serious. For example, nausea that persists for several hours may be considered severe nausea, but not a SAE. On the other hand, a stroke that results in only a limited degree of disability may be considered a mild stroke but would be a SAE.

Causality collection

The investigator will assess causal relationship between investigational product and each adverse event, and answer "yes" or "no" to the question "Do you consider that there is a reasonable possibility that the event may have been caused by the investigational product?"

For SAEs causal relationship will also be assessed for other medication and study procedures. Note that for SAEs that could be associated with any study procedure the causal relationship is implied as "yes".

A guide to the interpretation of the causality question is found in Appendix B of this Clinical Study Protocol.

AEs based on signs and symptoms

All AEs spontaneously reported by the subject or reported in response to the open question from the study personnel: *"Have you had any health problems since you were last asked?"*, or revealed by observation will be collected and recorded in the CRF. When collecting AEs, the recording of diagnoses is preferred (when possible) to recording a list of signs and symptoms. However, if a diagnosis is known and there are other signs or symptoms that are not generally part of the diagnosis, the diagnosis and each sign or symptom will be recorded separately.

AEs based on examinations and tests

The results from protocol mandated laboratory tests, vital signs, ECGs and other safety assessments will be summarised in the clinical study report. Deterioration as compared to baseline in protocol-mandated laboratory values, vital signs, ECGs and other safety

assessments should therefore only be reported as AEs if they fulfill any of the SAE criteria or are the reason for discontinuation of treatment with the investigational product.

If deterioration in a laboratory value or vital sign is associated with clinical signs and symptoms, the sign or symptom will be reported as an AE and the associated laboratory result or vital sign will be considered as additional information. Wherever possible the reporting investigator uses the clinical, rather than the laboratory term (e.g., anaemia versus low haemoglobin value). In the absence of clinical signs or symptoms, clinically relevant deteriorations in non-mandated parameters should be reported as AE(s).

Any new or aggravated clinically relevant abnormal medical finding at a physical examination as compared with the baseline assessment will be reported as an AE.

NB. Cases where a subject shows an AST **or** ALT ≥3xULN **or** total Bilirubin ≥ 2xULN may need to be reported as SAEs, please refer to Appendix E 'Actions required in cases of combined increase of Aminotransferase and Total Bilirubin - Hy's Law', for further instructions.

6.3.4 Reporting of SAEs

All SAEs have to be reported, whether or not considered causally related to the investigational product, or to the study procedure(s). All SAEs will be recorded in the CRF.

If any SAE occurs in the course of the study, then investigators or other site personnel inform appropriate AstraZeneca representatives immediately, or **no later than 24 hours** of when he or she becomes aware of it.

The designated AstraZeneca representative works with the investigator to ensure that all the necessary information is provided to the AstraZeneca patient safety data entry site **within one calendar day** of initial receipt for fatal and life threatening events and **within five calendar days** of initial receipt for all other SAEs.

For fatal or life-threatening adverse events where important or relevant information is missing, active follow-up is undertaken immediately. Investigators or other site personnel inform AstraZeneca representatives of any follow-up information on a previously reported SAE immediately, or **no later than 24 hours** of when he or she becomes aware of it.

Investigators or other site personnel send relevant CRF modules by fax to the designated AstraZeneca representative.

6.3.5 Laboratory safety assessment

Blood and urine samples for determination of laboratory safety assessments (clinical chemistry, haematology, and urinalysis) will be taken at the times indicated in the Study Plans (see Table 1 and Table 2). The date and time of collection of all laboratory tests will be recorded in the appropriate CRF.

The following laboratory variables will be measured:

Clinical chemistry	Haematology
Serum (S)-ALT	Blood (B)-Haemoglobin
S-AST	B-HbA1c
S-ALP	B-Eosinophil count
S-Bilirubin, total	B-Leukocyte
S-Calcium, total	B-Absolute leukocyte differential count
S-Creatinine	B-Platelet count
S-Fasting glucose	
S-Potassium	
S-Sodium	**Urinalysis**
S-Total Cholesterol	Urine (U)-Glucose - dipstick
S-Fasting Triglycerides	U-Haemoglobin
S-fasting Free Fatty Acids	U-Protein
S-fasting Insulin	U-Pregnancy test
S-CRP	

Additionally, at enrolment (Visit 1), all subjects will be tested for HIV, hepatitis B surface antigen and antibodies to hepatitis C. Urine will be tested for the following drugs of abuse at screening and admission: amphetamines, barbiturates, tricyclic antidepressants, cocaine, methadone, morphine, phencyclidine, tetrahydrocannabinol and opiates. At enrolment (Visit 1) and on admission (Day -3) the subject will be screened for alcohol. A urine pregnancy test will be performed at enrolment (Visit 1), on admission (Visit -3) and at follow-up (Visit 3). If a subject tests positive to any of these screening tests he/she will be excluded from the study.

Laboratory values outside the reference limit suspected to be of any clinical significance will be repeated (e.g., ALT >2 x ULN, ALP >ULN, bilirubin >1.5 x ULN). Subjects in who suspected clinical significance is confirmed will either not be included or if already randomised will be followed until normalisation or for as long as the investigator considers necessary. Additional laboratory variables may be performed for safety reasons if judged appropriate by the investigator.

NB. In case a subject shows an AST **or** ALT ≥3xULN **or** total bilirubin ≥2xULN please refer to Appendix E 'Actions required in cases of combined increase of Aminotransferase and Total Bilirubin - Hy's Law', for further instructions.

The samples for clinical chemistry, haematology and urinalysis will be analysed using routine methods at the local laboratory and will be disposed of after analysis. Urinalysis will be performed on glucose, protein and haemoglobin.

For blood volumes see Section 7.1.

6.3.6 Physical examination

The timings of individual examinations are indicated in the overall Study Plan (Table 1).

A complete physical examination will be performed at screening and follow-up and include an assessment of the following: general appearance, skin, head and neck, lymph nodes, thyroid, abdomen, musculo-skeletal, cardiovascular, respiratory and neurological systems.
On admission prior to dosing and at discharge from the unit only a brief physical examination will be conducted and will consist of measurement of blood pressure and pulse and assessment of general appearance.

Height will be measured in centimeters and weight in kilograms. Measurements should be taken without shoes and, if possible, the same scale used for all measurements. BMI will be calculated from the height and weight.

6.3.7 ECG

For timing of assessments refer to the Study Plans (Table 1 and Table 2).

6.3.7.1 Resting 12-lead ECG

At the enrolment visit, on admission (Day –3) and at the follow-up visit, 12-lead paper ECGs (pECG) will be obtained after a 10-minute supine rest. In addition, a 10-second paper ECG print-out from the 12-lead ECGs will be taken pre-dose, for overall evaluation (normal/ abnormal), before the study drug administration on Days 3 and 9. Only the overall evaluation will be recorded in the CRF.

The investigator may add extra 12-lead ECG safety assessments if there are any abnormal findings or if the investigator considers it is required for any other safety reason.

6.3.8 Vital signs

6.3.8.1 Pulse and blood pressure

Supine blood pressure and pulse will be measured using a semi-automatic blood pressure recording device with an appropriate cuff size after a 10-minute rest on a bed. For timings of assessments refer to the Study Plans (Table 1 and Table 2).

6.3.8.2 Body temperature

Body temperature will be collected as part of the vital signs assessment. For timings refer to the Study Plans (Table 1 and Table 2).

6.3.9 Other safety assessments

Blood glucose samples to be collected in the occurrence of hypoglycaemic symptoms according to the investigators judgment. If no timely blood glucose samples can be taken can capillary glucose measurements be performed with a calibrated glucose meter.

6.4 Pharmacokinetics

6.4.1 Collection of PK samples

Venous blood samples (approximately 3 mL/analyte) for the determination of concentrations of AZD1981 and paracetamol in plasma will be taken at the times presented in the Study Plans given in Table 2 and Table 3.

The date and time of collection of each sample will be recorded in the electronic data capture system, on eCRFs. Samples will be collected, labelled, stored and shipped as detailed in the Laboratory Manual.

Samples for determination of AZD1981 and paracetamol concentration in plasma will be analysed by COVANCE on behalf of Clinical Bioanalysis Alliance, AstraZeneca R&D, using appropriate bioanalytical methods. Full details of the analytical methods used will be described in a separate bioanalytical report.

For blood volumes see Section 7.1.

6.4.2 Determination of drug concentration in PK samples

Blood sampling for determination of AZD1981 in plasma will be performed on Days 2, 3, 8 and 9 (Table 1). All samples still within the known stability of the analytes of interest (i.e. AZD1981) at time of receipt by the bioanalytical laboratory will be analysed.

Placebo samples will not be analysed unless specified.

Additional analyses may be conducted on the biological samples to further investigate the presence and/or identity of drug metabolites. Any results from such analyses may be reported separately from the clinical study report.

6.5 Pharmacodynamics

6.5.1 Collection of PD samples

Pharmacodynamic samples include the following samples taken during the MMTT and GGI/GLP1 tests: Glucose, Insulin, C-peptide (4 mL), Glucagon (2 mL), GLP1 (2 mL) as well as samples for 7p Glucose (0.5 mL) curve up to 24 h. The samples collected for PD assessments will be analysed by the Profil Institute or a designated and qualified CRO.

Exploratory biomarker samples (5 mL) will include analyses of P-PGD2 metabolites, P-SHBG, B-Eosinophils and U-PGD2 metabolites as well as plasma samples for future exploratory analyses.

Exploratory biomarker samples, except for B-Eosinophils, will be sent to AZ for analysis and storage. For details see Section 7.2. B-Eosinophils will be determined at the local laboratory as part of the safety laboratory screen.

Samples will be collected, labelled, stored and shipped as detailed in the Laboratory Manual.

For sampling days and time schedules refer to Table 1, Table 2 and Table 3.

For blood volumes see Section 7.1.

6.6 Pharmacogenetics

See Appendix D of this Clinical Study Protocol.

For blood volume see Section 7.1.

7. BIOLOGICAL SAMPLING PROCEDURES

7.1 Volume of blood

The total volume of blood that will be drawn from each subject in this study is as follows:

Table 5 Volume of blood to be drawn from each subject

Assessment	Sample volume (mL)	No. of samples	Total volume (mL)
Safety:			
Clinical chemistry	5	5	25
Haematology	2	5	10
Serology (HIV, Hepatitis B and C)	5	1	5
AZD1981 Pharmacokinetics (PK)	3	18	54
Paracetamol PK	3	16	48
Pharmacodynamics (PD):			
Total GLP1	2	20	40
Glucagon	2	32	64
7p glucose	0.5	36	18
Glucose, C-peptide, insulin	4	76	304
Exploratory Biomarkers[a]	5	4	20
Pharmacogenetics	10	1	10
Total			598

a PGD2 metabolites, SHBG, S.Eosinophils & future use

The number of samples taken, as well as the volume required for each analysis, may be changed during the study as new data on AZD1981become available. However, the maximum volume to be drawn from each subject will not exceed 600 mL.

7.2 Handling, storage and destruction of biological samples

The samples will be used up or disposed of after analyses, or retained for further use as described below.

7.2.1 PK samples

Pharmacokinetic and paracetamol samples received by the Bioanalysis group will be disposed of after the Bioanalytical Report finalization or 6 months after issuance of the draft Bioanalytical Report (whichever is earlier), unless requested for future analyses. Pharmacokinetic samples may be disposed of or destroyed and anonymised by pooling. Additional analyses may be conducted on the anonymised, pooled PK samples to further evaluate and validate the analytical method. Any results from such analyses may be reported separately from the CSR. Incurred sample reproducibility analysis, if any, will be performed alongside the bioanalysis of the test samples and reported in a separate bioanalytical report.

7.2.2 PD samples

Pharmacodynamic samples will be disposed of by Profil Institute after the CSR finalisation, unless requested for future analyses. Destruction must not take place unless the responsible person at AstraZeneca has approved it.

7.2.3 Pharmacogenetic samples

See Appendix D of this Clinical Study Protocol.

7.2.4 Exploratory biomarkers

Exploratory biomarker samples include analysis of P-PGD2 metabolites, P-SHBG, B-Eosinophils and U-PGD2 metabolites as well as plasma samples for future exploratory analysis.

The biomarker samples, except for B-Eosinophils will be shipped for storage in the AstraZeneca R&D Mölndal Biobank and subsequent analysis by the Cardiovascular and Metabolic Disease (CVMD) Translational Science Laboratory.

The exploratory biomarker samples can be retained on behalf of AstraZeneca R&D for a maximum of 15 years following the last subject’s last visit, after which they will be destroyed. The results from future analyses will not be reported in the Clinical Study Report but separately in a Clinical Study Report Addendum/Scientific Report or Scientific Publication.

7.3 Labelling and shipment of biohazard samples

The Principal Investigator ensures that samples are labelled and shipped in accordance with the Laboratory Manual, and the Biological Substance, Category B regulations (materials containing or suspected to contain infectious substances that do not meet Category A criteria), see Appendix C of this Clinical Study Protocol ‘IATA 6.2 Guidance Document’.

Any samples identified as Infectious Category A materials are not shipped and no further samples will be taken from the subject unless agreed with AstraZeneca and appropriate labelling, shipment and containment provisions are approved.

7.4 Chain of custody of biological samples

A full chain of custody is maintained for all samples throughout their lifecycle.

The Principal Investigator keeps full traceability of collected biological samples from the subjects while in storage at the centre until shipment or disposal (where appropriate) and keeps documentation of receipt of arrival.

The sample receiver keeps full traceability of the samples while in storage and during use until used, disposed of or until further shipment and keeps documentation of receipt of arrival.

AstraZeneca keeps oversight of the entire life cycle through internal procedures, monitoring of study sites and auditing of external laboratory providers.

Samples retained for further use are registered in the AstraZeneca Biobank system during the entire life cycle.

7.5 Withdrawal of informed consent for donated biological samples

If a subject withdraws consent to the use of donated biological samples, the samples will be disposed of/destroyed, if not already analysed and the action documented. If samples are already analysed, AstraZeneca is not obliged to destroy the results of this research.

As collection of donated biological samples is an integral part of the study then the subject is withdrawn from further study participation.

The Principal Investigator:

- Ensures subject's withdrawal of informed consent to the use of donated samples is notified immediately to AstraZeneca
- Ensures that biological samples from that subject, if stored at the study site, are immediately identified, disposed of/destroyed and the action documented
- Ensures the laboratory(ies) holding the samples is/are informed about the withdrawn consent immediately and that samples are disposed of/destroyed, the action documented and the signed document returned to the study site
- Ensures that the subject and AstraZeneca are informed about the sample disposal

8. ETHICAL AND REGULATORY REQUIREMENTS

8.1 Ethical conduct of the study

The study will be performed in accordance with ethical principles that have their origin in the Declaration of Helsinki and are consistent with International Conference on Harmonisation (ICH) Good Clinical Practice, applicable regulatory requirements and the AstraZeneca policy on Bioethics and Human Biological Samples.

8.2 Subject data protection

The Informed Consent Form (ICF) will incorporate (or, in some cases, be accompanied by a separate document incorporating) wording that complies with relevant data protection and privacy legislation.

See Appendix D of this Clinical Study Protocol for additional precautions for genetic data.

8.3 Ethics and regulatory review

The Institutional Review Board (IRB) should approve the final study protocol, including the final version of the ICF and any other written information and/or materials to be provided to the subjects. The investigator will ensure the distribution of these documents to the applicable IRB, and to the study site staff.

The opinion of the IRB should be given in writing. The investigator should submit the written approval to AstraZeneca before enrolment of any subject into the study.

The IRB should approve all advertising used to recruit subjects for the study.

AstraZeneca should approve any modifications to the ICF that are needed to meet local requirements.

If required by local regulations, the protocol should be re-approved by the IRB annually.

Before enrolment of any subject into the study, the final study protocol, including the final version of the ICF, is approved by the national regulatory authority or a notification to the national regulatory authority is done, according to local regulations.

AstraZeneca will handle the distribution of any of these documents to the national regulatory authorities.

AstraZeneca will provide the Regulatory Authority, IRB and Principal Investigator with safety updates / reports according to local requirements, including SUSARs (Suspected Unexpected Serious Adverse Reactions), where relevant.

The Principal Investigator is also responsible for providing the IRB with reports of any serious and unexpected adverse drug reactions from any other study conducted with the

investigational product. AstraZeneca will provide this information to the Principal Investigator so he/she can meet these reporting requirements.

8.4 Informed consent

Any incentives for subjects who participate in the study as well as any provisions for subjects harmed as a consequence of study participation should be described in the informed consent form that is approved by an Ethics Committee.

The Principal Investigator will:

- Ensure that each subject is given full and adequate oral and written information about the nature, purpose, possible risk and benefit of the study
- Ensure that each subject is notified that they are free to withdraw from the study at any time
- Ensure that each subject is given the opportunity to ask questions and allowed time to consider the information provided
- Ensure each subject provides signed and dated informed consent before conducting any procedure specifically for the study
- Ensure the original, signed ICF(s) is/are stored in the Investigator's Study File
- Ensure a copy of the signed ICF(s) is/are given to the subject

8.5 Changes to the protocol and ICF

Study procedures will not be changed without the mutual agreement of the investigator and AstraZeneca.

If there are any substantial changes to the study protocol, then these changes will be documented in a study protocol amendment and where required in a new version of the study protocol (Revised Protocol).

The amendment should be approved by the IRB and if applicable, also the national regulatory authority, before implementation. Local requirements should be followed for revised protocols.

AstraZeneca will distribute any subsequent amendments and new versions of the protocol to the Principal Investigator. For distribution to the IRB see Section 8.3.

If a protocol amendment requires a change to the ICF, AstraZeneca and the IRB should approve the revised ICF before the revised form is used.

If local regulations require, any administrative change will be communicated to or approved by the IRB.

8.6 Audits and inspections

Authorised representatives of AstraZeneca, a regulatory authority, or an IRB may perform audits or inspections at the centre, including source data verification. The purpose of an audit or inspection is to systematically and independently examine all study-related activities and documents, to determine whether these activities were conducted, and data were recorded, analysed, and accurately reported according to the protocol, Good Clinical Practice (GCP), guidelines of the International Conference on Harmonisation (ICH), and any applicable regulatory requirements. The investigator will contact AstraZeneca immediately if contacted by a regulatory agency about an inspection at the centre.

9. STUDY MANAGEMENT

9.1 Pre-study activities

Before the first subject is entered into the study, it is necessary for a representative of AstraZeneca to:

- Determine the adequacy of the facilities
- Determine availability of subjects appropriate for the study
- Discuss with the investigators (and other personnel involved with the study) their responsibilities with regard to protocol adherence, and the responsibilities of AstraZeneca or its representatives. This will be documented in a Clinical Study Agreement, or equivalent, between AstraZeneca and the investigator

9.2 Training of study site personnel

Before the first subject is entered into the study, an AstraZeneca representative will review and discuss the requirements of the Clinical Study Protocol and related documents with the investigational staff and also train them in any study specific procedures. Profil Institute Clinical Data Management will ensure necessary training for relevant study personnel in the electronic data capture system.

The Principal Investigator will ensure that appropriate training relevant to the study is given to all of the staff, and that any new information relevant to the performance of this study is forwarded to the staff involved.

The Principal Investigator will maintain a record of all individuals involved in the study (medical, nursing and other staff).

9.3 Monitoring of the study

During the study, a study monitor contracted by Profil Institute will have regular contacts with the study site, including visits to:

- Provide information and support to the investigators

- Confirm that facilities remain acceptable
- Confirm that the investigational team is adhering to the protocol, that data are being accurately and timely recorded in the CRFs, that biological samples are handled in accordance with the Laboratory Manual and that investigational product accountability checks are being performed
- Perform source data verification (a comparison of the data in the CRFs with the subject's medical records at the hospital or practice, and other records relevant to the study) including verification of informed consent of participating subjects. This will require direct access to all original records for each subject (e.g., clinic charts)
- Ensure withdrawal of informed consent to the use of the subject's biological samples is reported and biological samples are identified and disposed of/destroyed accordingly, and the action is documented, and reported to the subject

The AstraZeneca representative or contracted monitor will be available between visits if the investigators or other staff at the centre needs information and advice about the study conduct.

9.3.1 Source data

Source data is maintained onsite throughout the course of the trial. Following study completion, records are archived and stored at an off-site facility (Corodata).

9.4 Study agreements

The Principal Investigator should comply with all the terms, conditions, and obligations of the Clinical Study Agreement or equivalent for this study. In the event of any inconsistency between this Clinical Study Protocol and the Clinical Study Agreement or equivalent, the Clinical Study Protocol shall prevail with respect to the conduct of the study and the treatment of subjects and in all other respects, the terms of the Clinical study Agreement or equivalent shall prevail.

Agreements between AstraZeneca and the Principal Investigator should be in place before any study-related procedures can take place, or any subjects are enrolled.

9.4.1 Archiving of study documents

The investigator follows the principles outlined in the Clinical Study Agreement or equivalent.

9.5 Study timetable and end of study

The end of the study is defined as "the last visit of the last subject undergoing the study".

The study is expected to start in Q1 2015 and to end by Q2/Q3 2015.

The study may be terminated if the study procedures are not being performed according to Good Clinical Practice, or if recruitment is slow. AstraZeneca may also terminate the entire study prematurely if concerns for safety arise within this study or in any other study with AZD1981.

10. DATA MANAGEMENT

Data management will be performed by Profil Institute.

The Data Management Plan will describe the methods used to collect, check, validate and process clinical data in detail. It will also clarify the roles and responsibilities for the different functions and personnel involved in the data management process. Furthermore the Data Management Plan will describe the data flow and timelines within the study.

Data entered into Profil Institute's electronic data capture system will be immediately saved to the applicable database and changes tracked to provide an audit trail. Data queries will be raised for inconsistent, impossible or missing data.

The data collected through third party sources will be obtained and reconciled against study data.

Data associated with biological samples will be transferred to laboratories internal or external to AstraZeneca.

Adverse events and medical/surgical history will be classified according to the terminology of the latest version of the Medical Dictionary for Regulatory Activities (MedDRA). Medications will be classified according to the latest AstraZeneca Drug Dictionary. Profil Institute's Clinical Data Management team will perform all coding.

Quality control procedures will be applied to each stage of data handling to ensure that all data are reliable and have been processed correctly.

When all data have been coded, validated, and electronically signed by the PI, the database may be locked and clean file will be declared. Any treatment revealing data may thereafter be incorporated into analysis files.

Further details about the handling of the clinical data and processes will be documented in the Data Management Plan.

See Appendix D of this Clinical Study Protocol for handling of genetic data.

11. EVALUATION AND CALCULATION OF VARIABLES

11.1 Calculation or derivation of safety variable(s)

11.1.1 Other significant AEs

During the evaluation of the AE data, an AstraZeneca medically qualified expert will review the list of AEs that were not reported as SAEs or AEs leading to discontinuation of investigational product and withdrawal from the study.. Based on the expert's judgment, significant adverse events of particular clinical importance may, after consultation with the Global Safety Physician, be considered OAEs and reported as such in the Clinical Study

Report. A similar review of other data from laboratory tests, vital signs, ECGs and other safety assessments will be performed for identification of OAEs.

Examples of these are marked haematological and other laboratory abnormalities, and certain events that lead to intervention (other than those already classified as serious), dose reduction or significant additional treatment.

11.2 Calculation or derivation of PK variables

11.2.1 PK of AZD1981

Pharmacokinetic analysis of the plasma concentration data for AZD1981 will be performed at COVANCE on behalf of AstraZeneca R&D. The actual sampling times will be used in the PK parameter calculations and PK parameters will be derived using standard non-compartmental methods using WinNonlin v5.2 or higher and/or SAS® version 9.1 or higher.

Where possible, the following PK parameters will be determined for AZD1981:

$C_{ss,max}$	Maximum plasma concentration (µmol/L) at steady state, obtained directly from the observed concentration versus time data
$t_{ss,max}$	Time of maximum plasma concentration at steady state (h), obtained directly from the observed concentration versus time data
$C_{ss,min}$	Minimum plasma concentration (µmol/L) at steady state, obtained directly from the observed concentration versus time data
CL_{ss}/F	Apparent oral plasma clearance (L/h), at steady state
AUC_{0-1h}	Area under the plasma concentration-time curve (µmol*h/L) from 0 h to 1 h of the dosing interval
AUC_{0-2h}	Area under the plasma concentration-time curve (µmol*h/L) from 0 h to 2 h of the dosing interval
AUC_{0-4h}	Area under the plasma concentration-time curve (µmol*h/L) from zero to 4 h of the dosing interval
AUC_{0-12h}	Area under the plasma concentration-time curve (µmol*h/L) from zero to 12 h of the dosing interval
AUC_{0-24h}	Area under the plasma concentration-time curve (µmol*h/L) from zero to 24 h of the dosing interval
AUC_{1-2h}	Area under the plasma concentration-time curve (µmol*h/L) from 1h to 2 h of the dosing interval

11.2.2 PK of Paracetamol

The analyses of plasma concentrations of paracetamol will be performed at COVANCE. The actual sampling times will be used in the parameter calculations. Pharmacokinetic parameters will be derived using standard non-compartmental methods using WinNonlin version 5.2 or higher and/or SAS® version 9.1 or higher.

The following parameters will be calculated for paracetamol:

C_{max}	Maximum concentration in the plasma (µmol/L), obtained directly from the observed concentration versus time data
t_{max}	Time of maximum plasma concentration (h), obtained directly from the observed concentration versus time data
$AUC_{(0\text{-}t)}$	Area under the concentration-time curve in plasma from zero (predose) to time of last quantifiable concentration (µmol*h/L), calculated by linear up/ log down trapezoidal summation. A minimum of 4 quantifiable post-dose concentration values will be required for AUC calculation.

11.3 Calculation or derivation of PD variables

Pharmacodynamic analysis of the primary and secondary pharmacodynamic data will be performed at Profil Institute on behalf of AstraZeneca R&D. The actual sampling times will be used in the PD parameter calculations.

Where possible, the following primary PD parameters will be determined:

MMTT $AUC_{Glc\ 0\text{-}4h}$	Area under the glucose plasma concentration-time curve during a mixed meal tolerance test, (mmol*h/L) calculated by linear up/ log down trapezoidal summation from zero to 4h of the MMTT
MMTT Glc C_{max}	Maximum glucose plasma concentration (mmol/L) during a mixed meal tolerance test, obtained directly from the observed concentration versus time data
GGI $AUC_{C\text{-}pep\ 1\text{-}2h}$	Area under the C-peptide plasma concentration-time curve during a graded glucose infusion, (nmol*h/L) calculated by linear up/ log down trapezoidal summation from 1 to 2h of the GGI e.g. during the GLP1 infusion.

Where possible, the following secondary PD parameters will be determined:

MMTT $AUC_{Glu\ 0\text{-}4h}$	Area under the glucagon plasma concentration-time curve during a mixed meal tolerance test, (ng*h/L) calculated by linear up/ log down trapezoidal summation from zero to 4h of the MMTT
MMTT $AUC_{Ins\ 0\text{-}4h}$	Area under the insulin plasma concentration-time curve during a mixed meal tolerance test, (pmol*h/L) calculated by linear up/ log down trapezoidal summation from zero to 4h of the MMTT
GGI $AUC_{Glc\ 1\text{-}2h}$	Area under the glucose plasma concentration-time curve during a graded glucose infusion, (mmol*h/L) calculated by linear up/ log down trapezoidal summation from 1 to 2 h of the GGI e.g. during the GLP1 infusion.
GGI $AUC_{Ins\ 1\text{-}2h}$	Area under the insulin plasma concentration-time curve during a graded glucose infusion, (pmol*h/L) calculated by linear up/ log down

	trapezoidal summation from 1 to 2h of the GGI e.g. during the GLP1 infusion.
GGI $AUC_{Glc\ 0\text{-}1h}$	Area under the glucose plasma concentration-time curve during a graded glucose infusion, (mmol*h/L) calculated by linear up/ log down trapezoidal summation from 0 to 1 h of the GGI.
GGI $AUCIns_{\ 0\text{-}1h}$	Area under the insulin plasma concentration-time curve during a graded glucose infusion, (pmol*h/L) calculated by linear up/ log down trapezoidal summation from 0 to 1h of the GGI.
$AUC_{Glc\ 0\text{-}24h}$	Area under the glucose plasma concentration-time curve, (mmol*h/L) calculated by linear up/ log down trapezoidal summation from zero to 24 h.

For the three parameters listed below, the analysis details will be specified in the Statistical Analysis Plan (SAP).

Fasting β-cell responsiveness

It is calculated as fasting C-peptide secretion (per unit volume of the central compartment) divided by fasting plasma glucose concentration before the start of the MMTT.

Postprandial β cell responsiveness

It equals the increment in insulin secretion (again per unit volume of the central compartment) in response to a unit increment in glucose concentration during MMTT.

GGI β cell responsiveness

It equals the increment in insulin secretion rate (again per unit volume of the central compartment of C-peptide) in response to a unit increment in glucose concentration during the GGI.

Further on, additional pharmacodynamic variables will be specified in the SAP.

11.4 Calculation or derivation of the relationship between PK and PD variables

The relationship between AZD1981 exposure and effect on the PD variables will be primarily explored graphically. Specifically, the following plots may be included in the analysis:

- MMTT $\Delta AUC_{PD\ 0\text{-}4h}$ vs PK-$AUC_{0\text{-}4h}$ (change in the area under the pharmacodynamic variable (such as glucose, C-peptide)-time curve between 0-4h (i.e. during MMTT) plotted against the area under the AZD1981 plasma concentration-time curve during the corresponding time interval)
- GGI $\Delta AUC_{PD\ 0\text{-}1h}$ vs PK-$AUC_{0\text{-}1h}$ (change in the area under the pharmacodynamic variable -time curve between 0-1h (i.e. during 1st hour of GGI) plotted against the

area under the AZD1981 plasma concentration-time curve during the corresponding time interval)

- GGI $\Delta AUC_{PD\ 1\text{-}2h}$ vs PK-$AUC_{1\text{-}2h}$ (change in the area under the pharmacodynamic variable -time curve between 1-2h (i.e. during 2nd hour of GGI/GLP1) plotted against the area under the AZD1981 plasma concentration-time curve during the corresponding time interval)

If appropriate, exposure-response relationships will be explored further.

11.5 Calculation or derivation of pharmacogenetic variables

See Appendix D of this Clinical Study Protocol.

12. STATISTICAL METHODS AND SAMPLE SIZE DETERMINATION

The statistical analysis will be performed by Profil Institute.

12.1 Description of analysis sets

12.1.1 General principles

The analysis of data will be based on different subsets according to the purpose of analysis, i.e., for safety, PK and PD, respectively. The decision regarding validity of data for each of the analysis sets will be based on a blind review of data.

The as-treated principle will be applied to all evaluations; i.e., subjects who received another treatment than the one assigned in the randomisation list will be analysed as belonging to the actual treatment group and not that assigned by randomisation.

12.1.2 Analysis of Safety population

All subjects who received at least one dose of randomised investigational product, AZD1981 or placebo and for whom any post-dose data are available will be included in the safety population.

12.1.3 PK analysis set

The PK analysis set will be based on all subjects who receive both doses of AZD1981 on the PK sampling days e.g. Day 3 or day 9. The PK analysis set should include all evaluable pharmacokinetic data appropriate for the evaluation of interest (with no major protocol deviations or violations thought to significantly affect the pharmacokinetics of the drug) from all subject who received investigational product. Subjects that receive placebo will not be part of the PK analysis set.

12.1.4 PD analysis set

The PD analysis set will be based on all patients who receive all doses of AZD1981. The PD analysis set should include all evaluable pharmacodynamic data appropriate for the evaluation of interest (with no major protocol deviations or violations thought to significantly affect the pharmacodynamics of the drug) from all subject who received investigational product.

12.2 Methods of statistical analyses

12.2.1 General principles

According to the primary objective two independent tests will be done in this study, i.e. mixed meal tolerance test (MMTT) and graded glucose infusion (GGI).

No adjustment for multiplicity will be done.

Missing data will be result in a reduced sample size for that parameter. Hence, no imputation of missing observation will be performed unless otherwise stated.

A subject who withdraws prior to the last planned observation in a study period will be included in the analyses up to the time of discontinuation.

A statistical analysis plan will be prepared before FSI.

12.2.2 Subject characteristics

Continuous variables will be summarised using descriptive statistics (n, mean, SD, min, median, max) by treatment group. Categorical variables will be summarised in frequency tables (frequency and proportion) by treatment group.

12.2.3 Safety and tolerability

Continuous variables will be summarised using descriptive statistics (n, mean, SD, min, median, max) by treatment group. Categorical variables will be summarised in frequency tables (frequency and proportion) by treatment group. Graphical presentations will be used as appropriate. Examples may include line graphs showing individual or mean development over time, and shift plots showing pre-treatment values on horizontal axis and post-treatment values on vertical axis.

SAEs will be collected for each subject from the time when informed consent is obtained (Visit 1) until and including the follow-up visit. Non-serious AEs will be collected from the start of dosing (Visit 2, Day 1) until and including the follow-up visit.

Adverse events will be summarised by PT (Preferred term) and SOC (System organ class) using MedDRA vocabulary. Furthermore, listings of serious adverse events and adverse events that led to withdrawal will be made and the number of subjects who had any adverse events, serious adverse events, adverse events that led to withdrawal, and adverse events with severe intensity will be summarised.

12.2.4 Pharmacokinetics

12.2.4.1 AZD1981

PK variables for AZD1981will be summarised using appropriate descriptive statistics (e.g. n, geometric mean, coefficient of variance (CV), min, median, max) by treatment group. The geometric mean is calculated as the exponential of the arithmetic mean calculated from data on a log scale. The CV is calculated as $100 \cdot \sqrt{(\exp(s^2)-1)}$ where s is the standard deviation of the data on a log scale. Graphical presentations of PK variables will be used as appropriate.

12.2.4.2 Paracetamol

A listing of paracetamol blood sampling times by individual will be provided. Subject listings of all plasma paracetamol concentration time data for each dose and sampling occasion will be presented. Plasma concentration versus time data will be presented graphically. Descriptive statistics will be presented for C_{max}, t_{max} and $AUC_{(0-t)}$.

12.2.5 Pharmacodynamics

12.2.5.1 Primary variables

The primary variables are MMTT $AUC_{Glc\ 0-4h}$ and GGI $AUC_{C-pep\ 1-2h}$.

The primary variables will be analysed using analysis of covariance (ANCOVA) models with treatment and period as factors and baseline of the primary variable and baseline of glucose as covariates. The primary variables will be log-transformed prior to the analysis and the results then transformed back to the linear scale, and significant tests will be 1-sided at 10% significance level.

Treatment estimates will be presented, and estimated mean ratios with 90% 1-sided confidence intervals and p-values will be presented for the treatment contrast of the one dose of AZD1981.

The analysis will be made using the PD analysis set.

12.2.5.2 Descriptive presentations related to the primary objective

Descriptive statistics (n, mean, median, min, max, q1 and q3) will be presented per treatment.

12.3 Determination of sample size

In crossover design, the intra-individual standard deviation for ln(MMTT $AUC_{Glc\ 0-4h}$) in T2DM subjects is estimated base on two sources. Estimation of standard deviation of ln(MMTT $AUC_{Glc\ 0-4h}$) form an internal study data (AR-H039242XX), is 0.091 and from a published study (Malloy et al 2009) is less than 0.15. Taking these two estimations into account, SD of ln(MMTT $AUC_{Glc\ 0-4h}$) is estimated equal to 0.1. For this SD and alpha 0.1, one-sided test and 8% difference versus control in MMTT $AUC_{Glc\ 0-4h}$, sample size of 20 evaluable subjects yields in at least 80% power.

On the other hand based on simulated data from a published study (Chang et al 2003), SD of ln(GGI $AUC_{C\text{-pept } 1\text{-}2\ h}$) was estimated as 0.17. With a similar test as the former one (MMTT $AUC_{Glc\ 0\text{-}4h}$), sample size of 20 subjects yields in at least 80% power to detect at least 20% difference versus control in GGI $AUC_{C\text{-pep } 1\text{-}2h}$.

Log-normal distribution was assumed for MMTT $AUC_{Glc\ 0\text{-}4h}$ and GGI $AUC_{C\text{-pep } 1\text{-}2h}$ when sample size was calculated.

13. IMPORTANT MEDICAL PROCEDURES TO BE FOLLOWED BY THE INVESTIGATOR

13.1 Medical emergencies and AstraZeneca/CRO contacts

The Principal Investigator is responsible for ensuring that procedures and expertise are available to handle medical emergencies during the study. **A medical emergency usually constitutes an SAE and is to be reported as such, see Section 6.3.4**

Medically responsible contacts, in the case of a medical emergency, are specified below:

Name	Role in the study	Address & telephone numbers
**AstraZeneca**		
Stanko Skrtic, MD, PhD, Senior Medical Science Director	AstraZeneca Study Team Physician responsible for the protocol at central R&D site	AstraZeneca R&D Mölndal<br>Early Clinical Development<br>CVMD Therapy Medical Unit<br>43183 Mölndal, Sweden<br>Mobile: +46 705 23943<br>Stanko.Skrtic@astrazeneca.com
**PROFIL™ Institute**		
Liliana Uribe-Bruce, MD, MCI, Research Physician	Principal Investigator	Profil™ Institute for Clinical Research, Inc.<br>Endocrinology, Diabetes & Metabolism<br>855 3rd Avenue, Suite 4400<br>Chula Vista, CA 91911, U.S.A<br>Phone: +1(619) 409-1277 or (619) 409-1261<br>Fax: +1(619) 872-2424<br>Liliana.uribe-bruce@profilinstitute.com
Elaine Watkins, DO, MSPH, Associate Medical Director	Medically responsible physician	Profil™ Institute for Clinical Research, Inc.<br>855 3rd Avenue, Suite 4400<br>Chula Vista, CA 91911 U.S.A.<br>Phone: +1(619) 409-1277 or (619) 409-1261<br>Fax: +1(619) 872-2424<br>elaine.watkins@profilinstitute.com

13.2 Overdose

There is no data on overdosing on AZD1981 and there is no known antidote. In the present study, doses in excess of 200 mg should be regarded as overdoses.

In case of an overdose, close medical supervision and monitoring should be continued until the patient recovers. In case of suspected overdose, the patient should be treated per standard medical practice based on the judgment of the PI. *In vitro* experiments indicate that AZD1981 is absorbed onto charcoal at conditions similar to those in the gastrointestinal tract.

In order to collect more information concerning excessive doses of AZD1981, a drug intake fulfilling the overdose definition needs to be reported to AstraZeneca as an overdose, regardless of clinical consequences.

- An overdose with associated AEs is recorded as the AE diagnosis/symptoms on the relevant AE modules in the CRF and on the Overdose CRF module
- An overdose without associated symptoms is only reported on the Overdose CRF module

If an overdose on an AstraZeneca study drug occurs in the course of the study, then investigators or other site personnel inform appropriate AstraZeneca representatives **within one day**, i.e., immediately but no later than **the end of the next business day** of when he or she becomes aware of it.

The designated AstraZeneca representative works with the investigator to ensure that all relevant information is provided to the AstraZeneca Patient Safety data entry site.

For overdoses associated with SAE, standard reporting timelines apply, see Section 6.3.4. For other overdoses, reporting should be done within 30 days.

13.3 Pregnancy

All pregnancies and their subsequent outcome (spontaneous miscarriage, elective termination, ectopic pregnancy, normal birth or congenital abnormality) should be reported to AstraZeneca using the appropriate forms.

13.3.1 Maternal exposure

Women of childbearing potential are not allowed to be included in this study. Should a pregnancy still occur, the investigational product should be discontinued immediately and the pregnancy reported to AstraZeneca. All outcomes of pregnancy must be reported to AstraZeneca on the pregnancy outcomes report form in the eCRF.

13.3.2 Paternal exposure

Male patients will be advised not to plan active procreation during the study period and for 3 months after last dose of IP.

Pregnancy of a subject's partner is not considered to be an adverse event. However, any conception occurring from the date of dosing until three months after dosing should be reported to AstraZeneca and followed up for its outcome.

14. LIST OF REFERENCES

Chang et al 2003
Chang AM, Jakobsen G, Sturis J, Smith MJ, Bloem CJ, An B et al. The GLP-1 Derivative NN2211 restores β-cell sensitivity to glucose in Type 2 Diabetic Patients after a single dose. Diabetes 2003;52(7):1786-91

Lindskog et al 2012
Lindskog C, Korsgren O, Pontén F, Eriksson JW, Johansson L, Danielsson A. Novel pancreatic beta cell-specific proteins: antibody-based proteomics for identification of new biomarker candidates. J Proteomics 2012;75(9):2611-20

Malloy et al 2009
Malloy J, Capparelli E, Gottschalk M, Guan X, Kothare P, Fineman M. Pharmacology and Tolerability of a Single Dose of Exenatide in Adolescent Patients With Type 2 Diabetes Mellitus Being Treated With Metformin: A Randomised, Placebo-Controlled, Single-Blind, Dose-Escalation, Crossover Study. Clinical Ther.2009;31(4):806-15

Norman 2014
Norman P. Update on the status of DP2 receptor antagonists; from proof of concept through clinical failures to promising new drugs. Expert Opin Invest Drugs 2014;23(1):55-66

Schmidt et al 2013
Schmidt JA, Bell FM, Akam E, Marshall C, Dainty IA, Heinemann A et al. Biochemical and pharmacological characterization of AZD1981, an orally available selective DP2 antagonist in clinical development for asthma. Br J Pharmacol.2013;168(7):1626-38
